# Evolutionarily conserved gene expression patterns for affective disorders revealed using cross-species brain transcriptomic analyses in humans, rats and zebrafish

**DOI:** 10.1038/s41598-022-22688-x

**Published:** 2022-12-02

**Authors:** Konstantin A. Demin, Nataliya A. Krotova, Nikita P. Ilyin, David S. Galstyan, Tatyana O. Kolesnikova, Tatyana Strekalova, Murilo S. de Abreu, Elena V. Petersen, Konstantin N. Zabegalov, Allan V. Kalueff

**Affiliations:** 1 Almazov National Medical Research Centre, Ministry of Healthcare of Russian Federation, St. Petersburg, Russia; 2 Institute of Translational Biomedicine, St. Petersburg State University, St. Petersburg, Russia; 3 Laboratory of Preclinical Bioscreening, Granov Russian Research Center of Radiology and Surgical Technologies, Ministry of Healthcare of Russian Federation, Pesochny, Russia; 4 Neurobiology Program, Sirius University of Science and Technology, Sochi, Russia; 5 University of Maastricht, Maastricht, The Netherlands; 6 Moscow Institute of Physics and Technologies, Moscow, Russia; 7 Institute of Neurosciences and Medicine, Novosibirsk, Russia; 8 Ural Federal University, Ekaterinburg, Russia

**Keywords:** Neuroscience, Translational research, Experimental models of disease

## Abstract

Widespread, debilitating and often treatment-resistant, depression and other stress-related neuropsychiatric disorders represent an urgent unmet biomedical and societal problem. Although animal models of these disorders are commonly used to study stress pathogenesis, they are often difficult to translate across species into valuable and meaningful clinically relevant data. To address this problem, here we utilized several cross-species/cross-taxon approaches to identify potential evolutionarily conserved differentially expressed genes and their sets. We also assessed enrichment of these genes for transcription factors DNA-binding sites down- and up- stream from their genetic sequences. For this, we compared our own RNA-seq brain transcriptomic data obtained from chronically stressed rats and zebrafish with publicly available human transcriptomic data for patients with major depression and their respective healthy control groups. Utilizing these data from the three species, we next analyzed their differential gene expression, gene set enrichment and protein–protein interaction networks, combined with validated tools for data pooling. This approach allowed us to identify several key brain proteins (GRIA1, DLG1, CDH1, THRB, PLCG2, NGEF, IKZF1 and FEZF2) as promising, evolutionarily conserved and shared affective ‘hub’ protein targets, as well as to propose a novel gene set that may be used to further study affective pathogenesis. Overall, these approaches may advance cross-species brain transcriptomic analyses, and call for further cross-species studies into putative shared molecular mechanisms of affective pathogenesis.

## Introduction

Stress evokes a wide range of behavioral, molecular and physiological responses^1–7^ in vivo, also triggering various affective disorders, including anxiety, depression and post-traumatic stress disorder (PTSD) clinically^8–11^. While these neuropsychiatric disorders are widespread, debilitating and often treatment-resistant^12–14^, their understanding is complicated by heterogeneity and unclear pathological mechanisms and risk factors^15,16^. To address these problems, animal (experimental) models are widely used for studying stress pathogenesis and recapitulating clinical affective disorders^17–19^.

Commonly utilizing various chronic unpredictable stress (CUS) protocols^20–24^, these experimental models typically involve rodents exposed to varying stressors for several weeks^22,24–27^, to evoke anxiety- and/or depression-like ‘affective’ behavioral and physiological alterations^28–30^ that resemble those observed clinically^31,32^. Recognized as an important novel model organism in the central nervous system (CNS) disease modeling, the zebrafish (*Danio rerio*) is widely used in translational biomedicine^33–36^. Complementing rodent neurobehavioral evidence, zebrafish are also becoming popular in stress research^37,38^. Their growing utility in this field is supported by the fact that zebrafish are highly homologous to humans both genetically and physiologically^39,40^, and have evolutionarily conserved neurotransmitter systems^41,42^ and neuromorphology^43,44^. Like rodents, zebrafish are currently widely used in modeling stress-related affective disorders^45–47^, typically utilizing various aquatic protocols, assays and tests adapted from those in rodents^48–52^.

However, all animal models are rather difficult to fully parallel in humans, necessitating novel methods of translating experimental modeling results into clinical setting. Aiming to target ‘core’, evolutionarily conserved pathogenesis, and recognizing the importance of cross-species analyses in CNS research^53,54^, here we performed an in-depth pilot cross-species/cross-taxon analysis of brain transcriptomic data in zebrafish, rats and humans, in order to identify putative novel ‘shared’ molecular targets for affective CNS disorders evoked by chronic stress.

## Results

**Figure 1:**
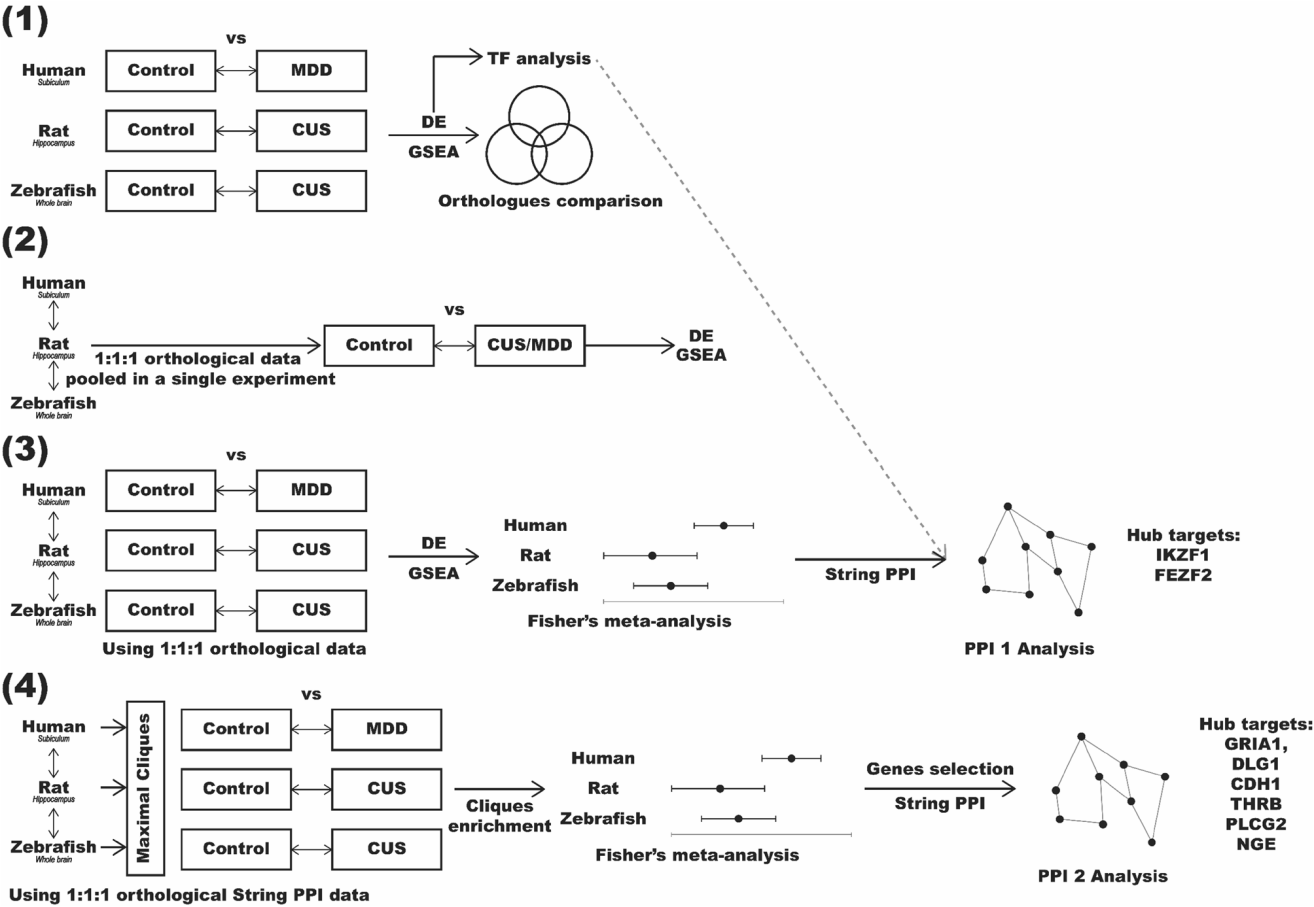
Schematic summary of the study design and analyses.

In general, our study aimed to identify common differentially expressed (DE) genes and/or enriched gene sets in contrasts between (1) human subiculum data from patients with major depressive disorder (MDD, based on DSM-IV criteria) vs. healthy controls available from NCBI's Gene Expression Omnibus (GEO) database^55^, (2) rat hippocampus samples following chronic unpredictable stress (CUS) vs. unexposed controls, and (3) zebrafish whole-brain CUS-exposed samples versus unexposed controls (see the “Methods” section for details, as well as Supplementary Table S1 and Fig. 1). The study utilized several different methods to establish potential relationships between the species-specific and cross-species data.

Briefly, Experiment 1 aimed to directly search for common patterns in the differential gene expression data (i.e., identifying DE genes for each species) and in gene set enrichment results (i.e., identifying the enriched sets for each species by analyzing the expression of sets of genes instead of individual genes), searching for similar genes (using orthologues) or gene sets (using Kyoto Encyclopedia of Genes and Genomes, KEGG^56^) in these data. Additionally, Experiment 1 analyzed the enrichment of DE genes for transcription factors DNA-binding sites (TFBSs) down- and up- stream from their genetic sequences.

Experiment 2 pooled all individual species' raw RNA sequencing (RNA-seq) data by their orthologs, generating combined ‘interspecies' data that were further analyzed for comparison between affective pathogenesis (MDD humans + CUS rats + CUS zebrafish) vs. controls (healthy humans + control rat + control zebrafish data). These data were further processed using the DE and gene set enrichment analyses, similar to Experiment 1.

Experiment 3 applied the Fisher’s meta-analysis approach^57^ to combine interspecies data. Briefly, the individual species-specific data were first processed using the DE or gene set enrichment analyses (using only the genes that have orthologues in all three species), and then the p-values obtained for each species were further included in Fisher’s meta-analysis^57^. Finally, Experiment 4 utilized another approach, as we generated protein–protein interaction network for selected identified common/shared genes that have orthologous in all three species. We next analyzed the maximal cliques (MCs, subgraphs in which all nodes are connected to each other, as in^58^) for this network, and used them as gene sets for further enrichment analysis (similar to other enrichment analyses described above, but using MCs instead of Kyoto Encyclopedia of Genes and Genomes (KEGG)^56^ sets). These sets were analyzed for enrichment for individual species data first and then further combined into interspecies data using the Fisher’s meta-analysis^57^.

**Figure 2:**
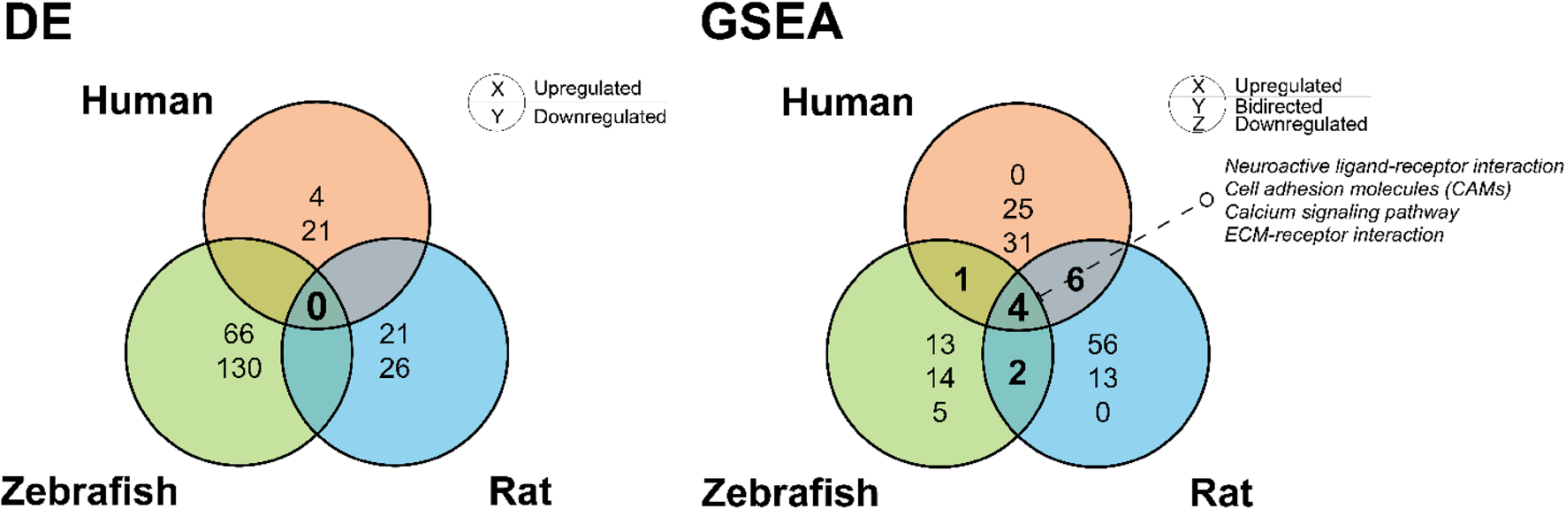
Venn diagrams for differentially expressed (DE) genes or enriched gene sets (GSEA, gene set enrichment analysis) using similar raw RNA-seq data counts comparing subiculum in human major depressive disorder (MDD) vs. control, hippocampi in rats exposed to chronic unpredictable stress (CUS) vs. unexposed controls, and in zebrafish CUS-exposed vs. unexposed control whole brain samples, using 1:1:1 orthologues from the HomoloGene Map^59^ (Experiment 1, see the “Methods” section for details). Overall, these direct species-to-species comparisons revealed no common DE genes and very few common DE gene sets.

Overall, Experiment 1 focused on direct species-to-species comparisons of brain gene expression data. Specifically, if some genes were DE in the experiment, we next evaluated whether their respective orthologues appear in another animal species or in clinical data. Utilizing traditional direct two-species comparisons of lists of gene orthologues using Venn diagrams, Experiment 1 revealed 25 DE genes for human subiculum, 47 for rat CUS hippocampus and 196 for zebrafish CUS whole brain samples (Supplementary Tables S1–S3). No orthologous DE genes were identified as shared/common between the species using the HomoloGene database^59^ (www.ncbi.nlm.nih.gov/homologene, see Fig. 2 and Supplementary Material 2 online). Generally Applicable Gene Set Enrichment (GAGE) analyses^60^, performed on raw data counts similarly to the DE gene analysis, identified 56 altered KEGG sets in human, 69 in rat, and 32 in zebrafish data (Fig. 2 and Supplementary Tables S4–S6). Sets that were simultaneously altered in all three species include *calcium signaling*, *extracellular matrix-receptor (ECM-receptor) interaction*, *cell adhesion molecules (CAMs)*, and *neuroactive ligand-receptor interaction* KEGG pathways (Fig. 2). Notably, one upregulated set (*oxidative phosphorylation*) was affected in both rat and zebrafish samples, and two downregulated pathways (*spliceosomes* and *RNA transport*) were common between humans and rats.

**Figure 3:**
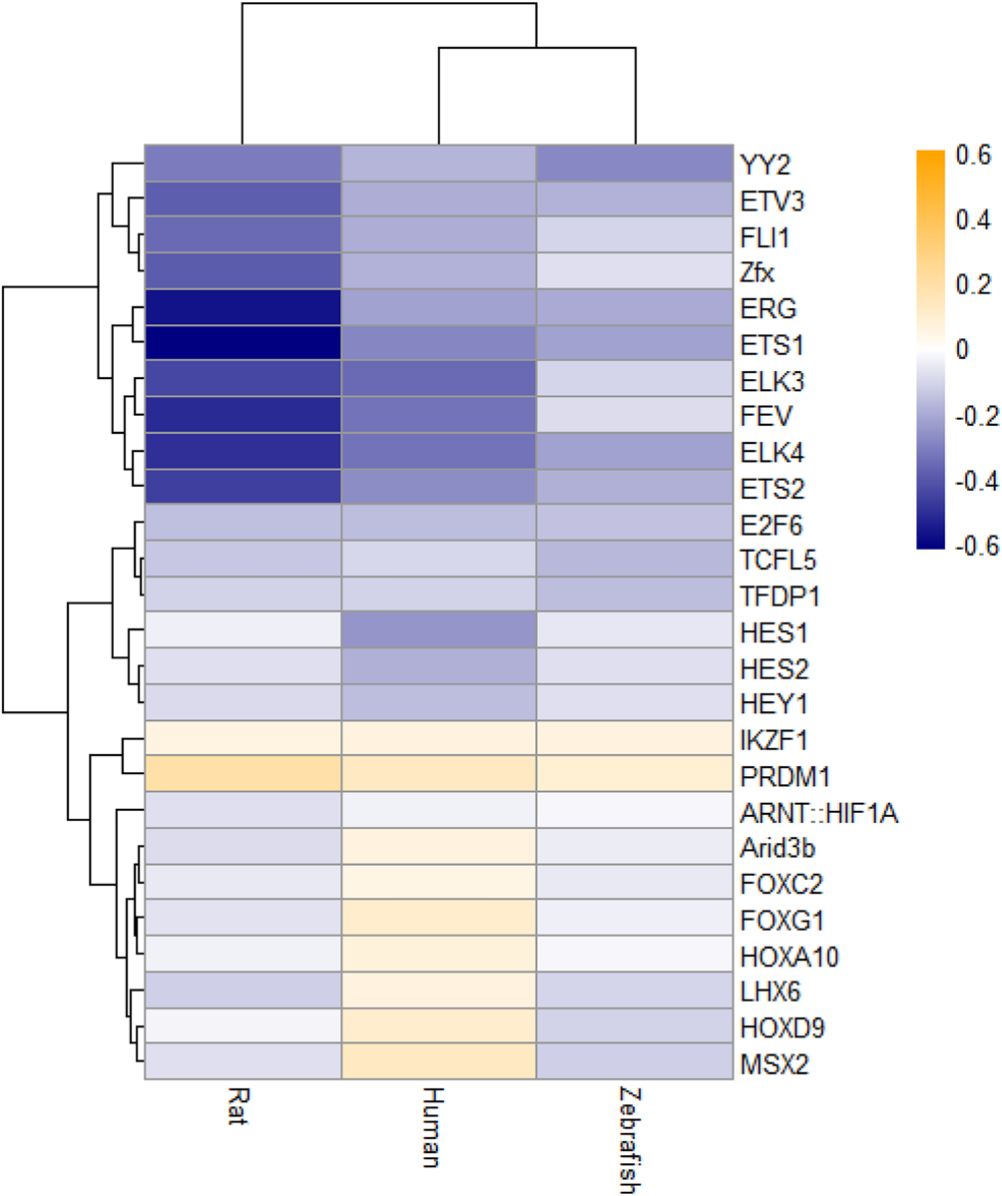
Summary of common/shared over- and under-represented transcription factors DNA binding sites (TFBSs) among differentially expressed (DE) genes with high (*p* < 0.01) vs. low (*p* > 0.7) statistical variability in human, rat, and zebrafish data using the CiiiDer TFMs software^61^ (Experiment 1). Only TFBSs with both *p* < 0.05 for gene coverage p-value and *p < 0.05* for the distribution of the number of TFBS, were considered significantly altered. TFBSs were sorted by their gene coverage p-value. Data are represented as log2-enrichment values, calculated according to the CiiiDer TFMs manual^61^.

While traditional DE analyses here yielded no common genes between the three species, studying enrichment of significantly altered genes for transcription factors DNA-binding sites (TFBSs) down- and up- stream from their genetic sequences (see the “Methods” section and^61^ for details) revealed 291 differentially represented (DR) human, 249 rat and 80 zebrafish TFBSs, with 19 DR TFBSs shared by all three species (Fig. 3 and Supplementary Tables S7–S9).

In general, the results of Experiment 1 show that direct comparisons of gene orthologues may not be an efficient approach to find commonalities in RNA-seq data between species-specific samples. Furthermore, given that TFBSs and GSEA analyses were similar to traditional DE gene analyses, utilizing data for a wide range of genes organized within specific established molecular pathways may be more informative to compare gene expression patterns between species.

**Figure 4:**
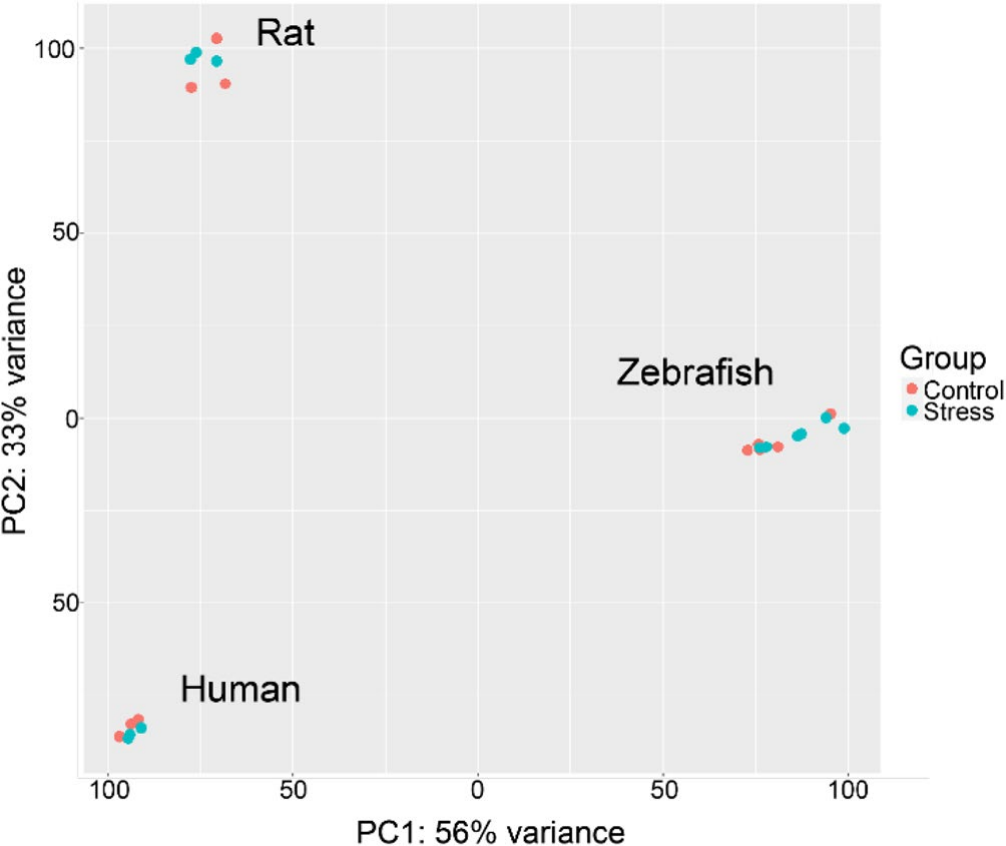
The principal component analysis **(**PCA) of Experiment 2 data, studying cross-species samples mapped using 1:1:1 orthologous map pooled into stress (major depressive disorder, MDD/chronic unpredictable stress, CUS) or control groups. Note high data heterogeneity that prevented direct combining cross-species data here, and the lack of clear separation between the groups in other principal components (PC) studied (data not shown). PC 1—principal component 1, PC 2—principal component 2.

Instead of comparing gene expression profiles as a post-hoc analysis, Experiment 2 pooled raw RNA-seq data counts, thus combining the species-specific data prior to any differential expression analysis, aiming to achieve better inter-species data compliance. To globally analyze gene expression data in all three species, we compiled a pooled interspecies list of counts for all their common orthologous genes, using the HomoloGene database map^59^ (see Supplementary Material 2 and www.ncbi.nlm.nih.gov/homologene), thus generating the combined ‘human MDD + rat CUS + zebrafish CUS' dataset of genes to be compared with the pooled control dataset consisting of control groups from all three species. Using such gene list, Experiment 2 yielded no differences in DE human orthologue genes by comparing stressed vs. normal controls for all three species (NS; *p* adjusted > 0.05). However, this lack of significant effects was rather unsurprising, given high heterogeneity of species-specific data, and the fact that their Principal Component Analysis (PCA) revealed most main effects as species-specific (Fig. 4). In contrast, our GAGE analysis^60^ of these data was more sensitive, yielding 91 altered molecular pathways (Supplementary Table S10). Importantly, these findings closely parallel data obtained earlier in Experiment 1 (Fig. 2, Supplementary Tables S4–S6), since all 4 sets (found to be enriched in all three species in Experiment 1) were similarly enriched in Experiment 2 (Fig. 2, Supplementary Tables S4–S6 and S10). Furthermore, among these 4 sets, three sets (*Neuroactive ligand-receptor interaction*, *calcium signaling* and *ECM-receptor interaction*) were the most altered in Experiment 2, supporting their likely high impact on affective pathogenesis in all three species.

Overall, Experiment 2, similarly to Experiment 1, yielded poor DE gene profiling results, hence calling for other tools to be applied to better combine and analyze the brain gene expression data. In contrast, GSEA was efficient in both Experiments 1 and 2, further supporting high efficiency of this approach to detect commonalities in RNA-seq data.

However, a key methodological issue of GAGE analyses utilized in Experiments 1 and 2 here was its reliance on the pre-set KEGG pathways, that may have no direct connection to affective pathogenesis per se, and can only partially correspond to the observed phenotypes. For example, while the KEGG *calcium signaling* pathway may relate to some disturbances in depression, it can neither fully explain nor recapitulate the disorder, and some of the genes within this pathway may have no effect on depression pathogenesis. To account for these limitations, Experiment 3 probed the ability of Fisher’s meta-analysis to combine p-values from different experiments (in order to improve data combination). Likewise, Experiment 4 also applied the graph theory-based MC analysis (see further), to obtain set enrichment results for targets that are more functionally related to the affective pathogenesis.

**Figure 5:**
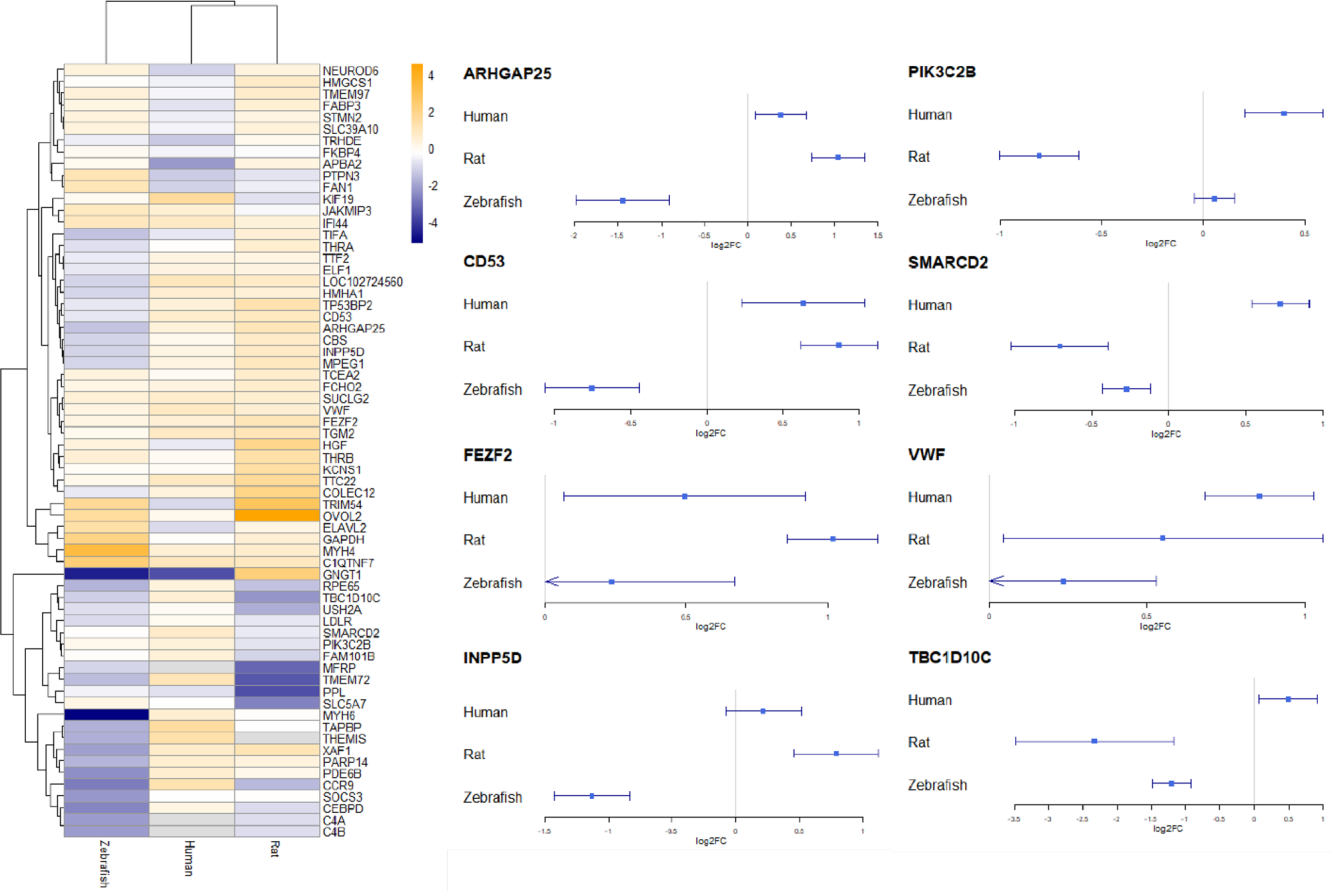
Heatmap representing log2 fold change of the genes significantly altered according to Fisher’s meta-analysis^57^ (left panel) or volcano plots for selected genes (right panel) in Experiment 3.

Fisher’s meta-analyses were efficient to compare combined interspecies DE genes' and gene sets enrichment* p*-values. In summary, examining the potential of Fisher’s meta-analysis^57^ to compare gene-orthologues data, Experiment 3 yielded 15 human, 29 rat, and 62 zebrafish DE orthologues (Supplementary Tables S11–S13). Fisher’s meta-analysis of these data identified 66 DE genes, including 15 DE genes altered in the same log2 fold change (l2fc) direction in all three species, supporting that they all changed their expression in a similar way across the species (Fig. 5 and Supplementary Table S14). While our GAGE analyses^60^ revealed 24 altered human, 42 rat and 109 zebrafish pathways (Supplementary Tables S15–S17), their Fisher’s meta-analysis^57^ identified 112 pathways shared by all three species (Supplementary Table S18).

**Figure 6:**
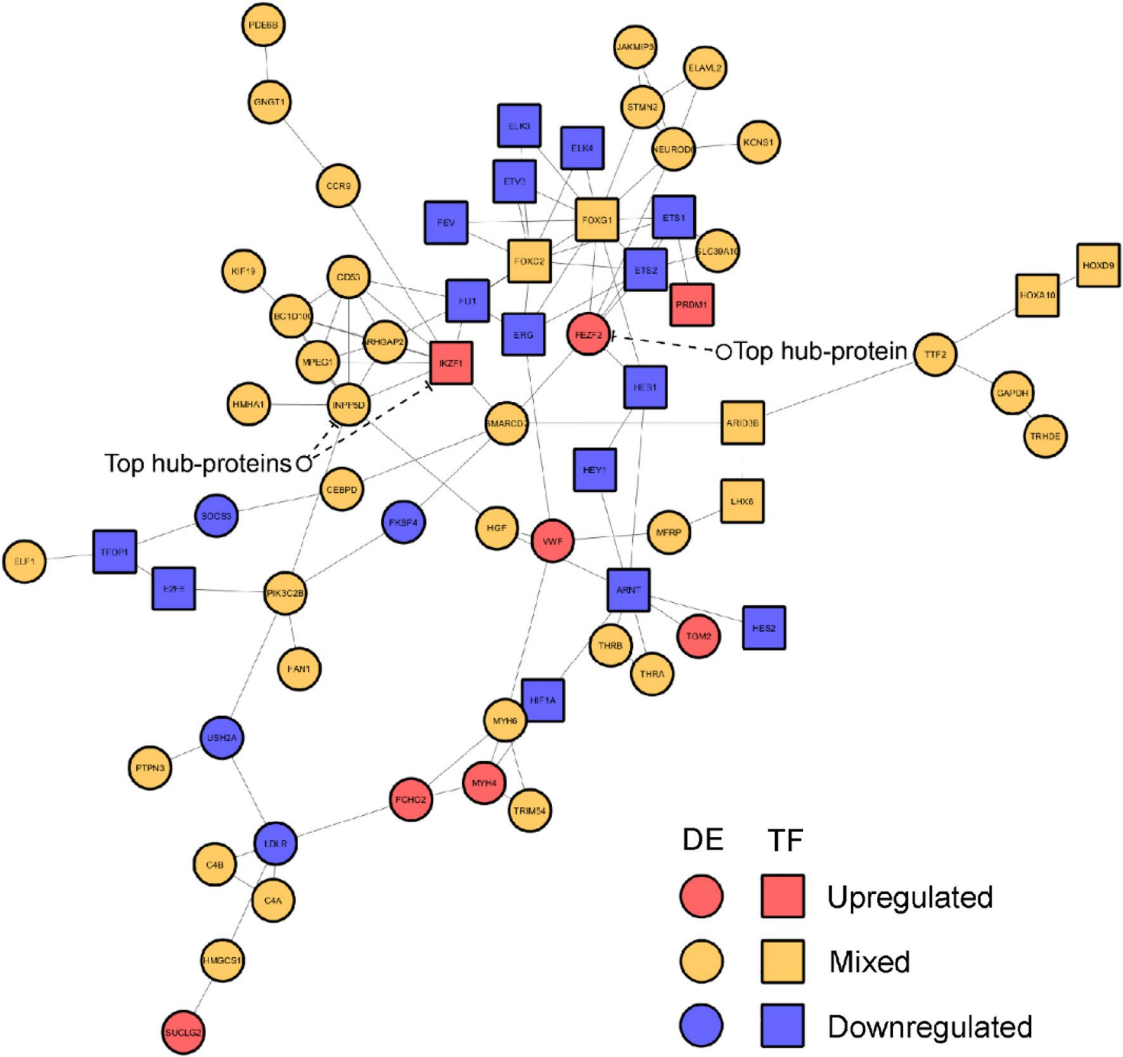
The network of protein–protein interactions (PPI) constructed for differentially expressed (DE) genes from Experiment 3 or differentially represented (DR) transcription factors (TF) binding sites (TFBSs) from Experiment 1, using the STRING online database^62^ (https://www.string-db.org/, see the “Methods” section and Fig. 3 and 5 for details).

To examine whether Fisher’s meta-analysis data at least partially correspond to direct differentially expressed (DE) gene analyses applied earlier (Experiment 1), we constructed the Protein–Protein Interaction (PPI) network for shared DE genes (identified in Experiment 3) and DR TFBSs (identified in Experiment 1) with high enrichment level (*p* = 8.31e − 09), using the Search Tool for the Retrieval of Interacting Genes/Proteins (STRING) online database^62^ (https://www.string-db.org/). Overall, these results support strong inter- and intra-connection between the TFBSs' Experiment 1 and DE meta-analyses' Experiment 3 data (Fig. 6). After ranking all vertices in the PPI networks by the Degree^63^, BottleNeck^64^, Betweenness and DMNC||MNC^65^ approaches (i.e., characterizing their overall ‘hubness’ within the network), we excluded genes with mixed direction (increased or decreased, compared to control) of expression changes in the stressed groups (see the “Methods” section for details), choosing only vertices that were highly ranked by at least two separate graph theory-based methods independently, thus yielding 4 proteins from the TFBS study and two proteins from the meta-analysis study as ‘hub’ vertices (Table 1), including 3 up-regulated or overrepresented (IKZF1, FEZF2, and VWF) and 3 down-regulated or underrepresented proteins (FLI1, ARNT and ERG, Fig. 5, 6).

**Table 1:** Top ten vertices analyzed using the Double Screening Scheme (DSS) analysis, combining the Density of Maximum Neighborhood Component (DMNC) and Maximum Neighborhood Component (MNC, see the “Methods” section for details; DMNC||MNC^65^), degree^63^ or bottleneck ^64^ approaches, for networks of constructed protein–protein interactions (PPI) from Experiments 1 and 3 (Fig. 6) and Experiment 4 (Fig. 10).

Proteins	DMNC	MNC	Proteins	Degree	Proteins	Bottle-neck	Proteins	Betweenness
**1st PPI experiment 1 (TFBSs) and experiment 3 (DE)**								
IKZF1	0.57	6	INPP5D	8	INPP5D	26	FEZF2	791.91
CD53	0.57	6	ARNT	8	HGF	16	INPP5D	756
ARHGAP25	0.57	6	IKZF1	8	FEZF2	15	PIK3C2B	746.78
INPP5D	0.65	5	TBC1D10C	6	PIK3C2B	15	SMARCD2	639.68
TBC1D10C	0.65	5	CD53	6	SMARCD2	15	ARNT	626.43
MPEG1	0.65	5	ARHGAP25	6	IKZF1	10	IKZF1	519.36
FLI1	0.46	3	FEZF2	5	MYH6	10	LDLR	479.63
NEUROD6	0.31	3	PIK3C2B	5	ERG	10	HGF	455.06
STMN2	0.31	3	LDLR	5	ARNT	9	USH2A	436.33
FEZF2	0.31	2	NEUROD6	5	USH2A	8	NEUROD6	399
**2nd PPI experiment 4**								
WNT8B	0.67	8	SRC	15	PRKACA	31	PRKACA	907.62
WNT3A	0.67	8	CTNNB1	12	SRC	11	CTNNB1	544.30
WNT2	0.67	8	PRKACA	11	CTNNB1	10	CACNA1C	456
WNT7A	0.77	7	CBL	9	CACNA1C	7	OXTR	390
WNT7B	0.77	7	WNT8B	8	OXTR	6	SRC	353.57
WNT10A	0.77	7	WNT5A	8	GRIA1	6	CBL	323.20
SFRP5	0.77	7	WNT3A	8	WNT8B	5	GRIA1	269.11
WNT5A	0.77	7	WNT2	8	CBL	5	COL7A1	168
MAPK11	0.41	7	MAPK14	8	GAB1	4	DLG1	161.54
TRHR	0.64	5	MAPK1	8	RARA	3	WNT5A	106.75

**Figure 7:**
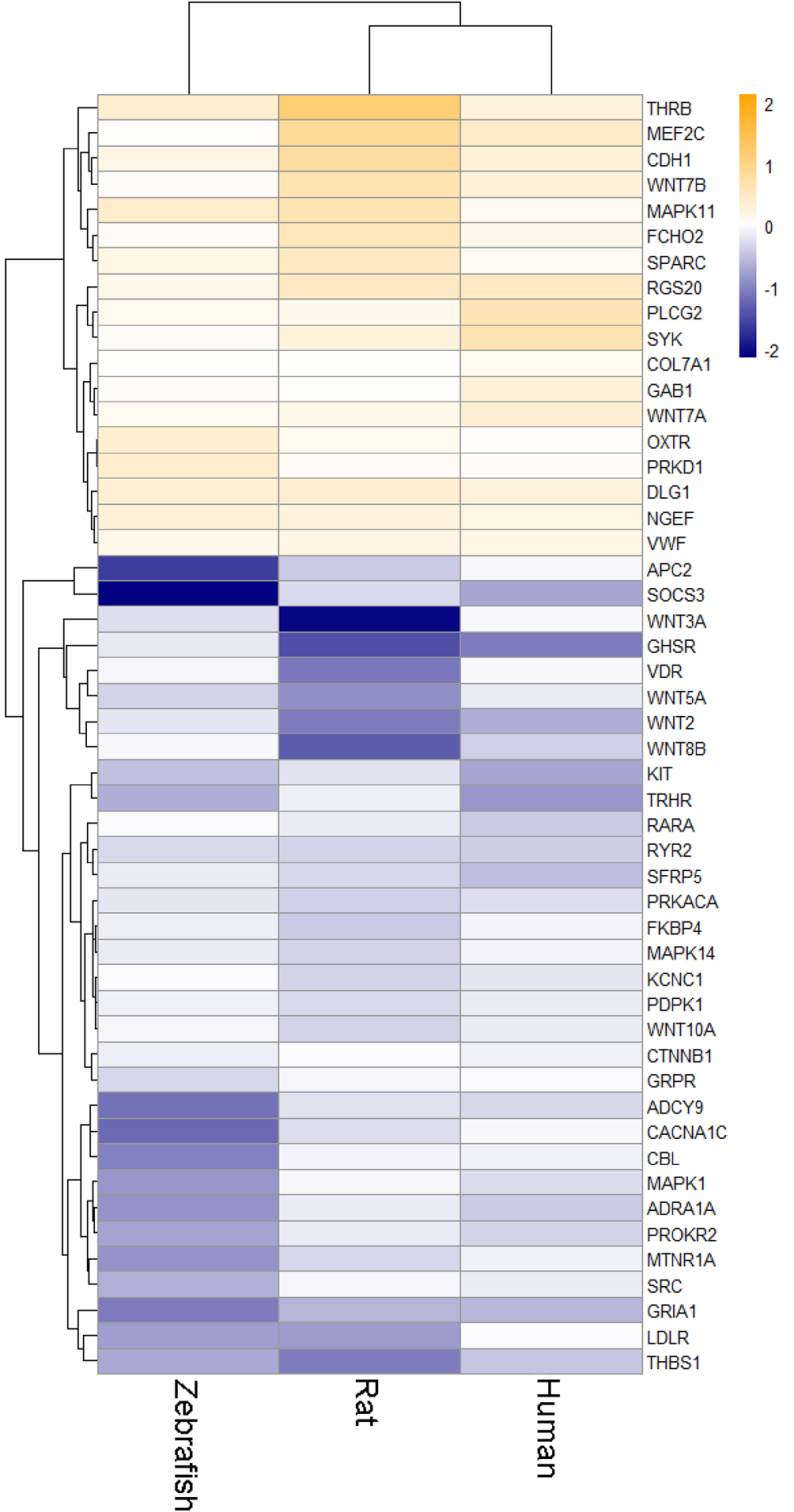
Genes expressed in a similar direction in the enriched sets using Fisher’s meta-analysis ^57^ on maximal cliques (MCs) Gene Set Enrichment Analysis (GSEA) data of major depressive disorder (MDD) vs. control human or chronic unpredictable stress (CUS) vs. control rat and zebrafish data, mapped to 1:1:1 human orthologue using the HomoloGene database^59^ (Experiment 4; www.ncbi.nlm.nih.gov/homologene). Data are presented as mean log2 fold change.

Experiment 4 aimed to establish novel, functionally more valid (than using the KEGG approach) sets of genes that may better correspond to the pathogenesis of affective disorders. In the graph theory, a clique is a subset of vertices of a graph such that every two distinct vertices in the clique are adjacent (connected), thus forming a complete subgraph^66^. MCs represent cliques that cannot be extended by including one more adjacent vertex, meaning that it is not a subset of a larger clique^58^. Applying the clique analyses here, Experiment 4 identified 121219 MCs for the PPI network constructed using the entire set of orthologous genes, including 21938 non-ribosomal MCs (with a maximum size of N = 29) that were further analyzed as gene sets using GAGE^60^, to compare their enrichment (bidirectionally altered, i.e., including both under- and over-representation in absolute values, regardless the direction of individual gene expression changes) in each species in affective (CUS/MDD) samples vs. control (Fig. 7 and Supplementary Material 3). Fisher’s meta-analysis of these data identified a total of 257 enriched MCs containing 253 unique proteins (Supplementary Table S20 and Fig. S2).

**Figure 8:**
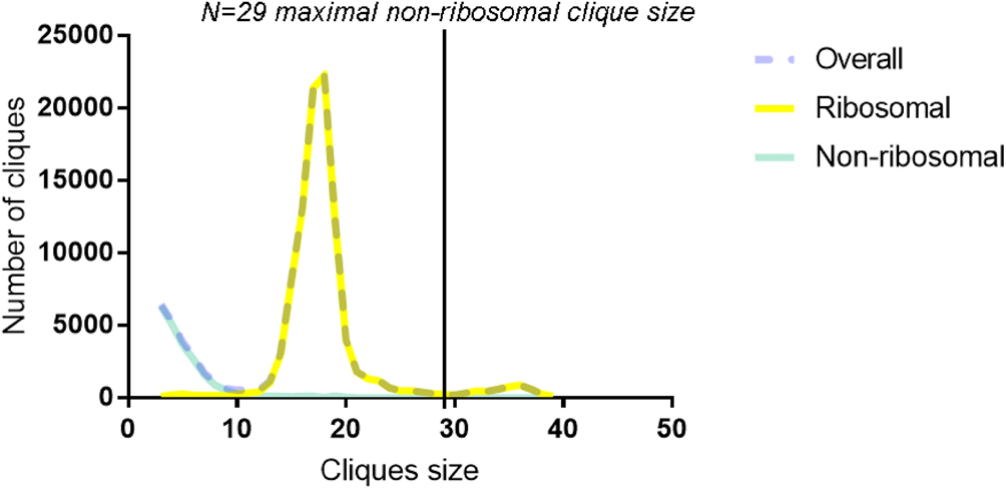
The number of maximal cliques (MCs) in the protein–protein interaction (PPI) network constructed from all orthologous genes mapped using 1:1:1 human:rat:zebrafish HomoloGene^59^ (www.ncbi.nlm.nih.gov/homologene) map in STRING^62^ (https://www.string-db.org/) and OmicsNet (www.omicsnet.ca/)^67^ databases.

As a proof-of-concept approach, we also applied GAGE analysis^60^ to the set containing these 253 genes, finding it to be significantly enriched in stress vs. control groups in all three species (Fig. 8 and Supplementary Table S21). Notably, this set was not only enriched in depressed human prefrontal cortex (PFC) data, but was also less enriched following antidepressant treatment in stressed animals, based on both zebrafish and rat data (drug vs. stress groups; Fig. 8 and Supplementary Table S21). Overall, this collectively indicates that this set was enriched in the original datasets even when analyzed using traditional methods (without any data combination), and may also have some predictive validity as well, given its responsivity to antidepressant treatment.

**Figure 9:**
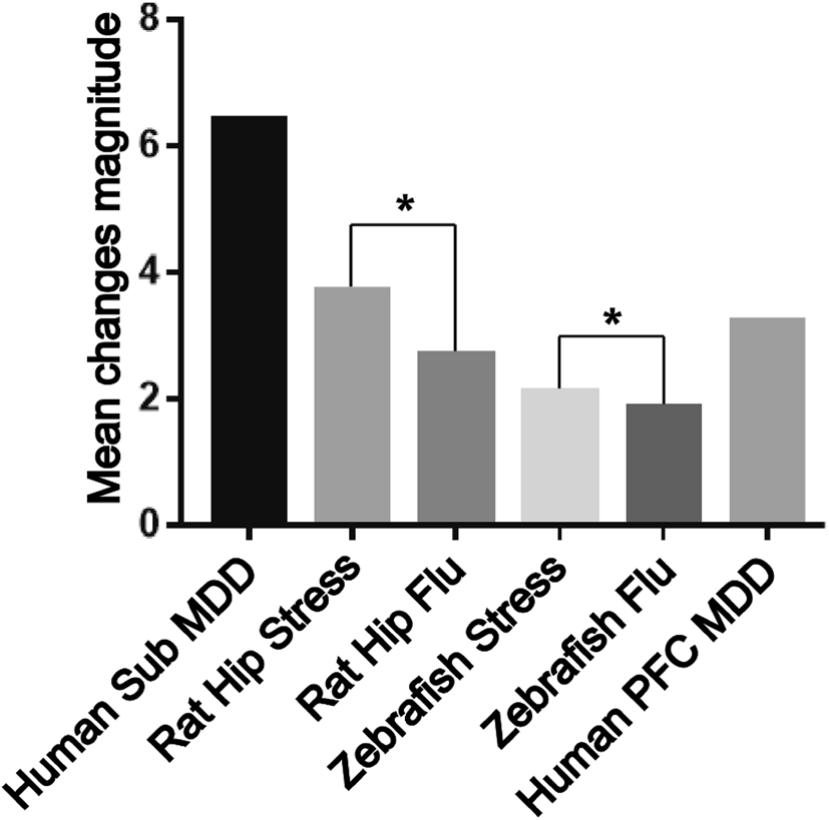
Results of Gene Set Enrichment Analyses (GSEA) comparing the set of 253 genes suggested using maximal cliques (MCs) and significantly altered in Fisher’s meta-analysis ^57^ (Experiment 4). All data significantly differed from their corresponding controls (*p* < 0.05). Asterisks denote additional significant differences between the groups (*p* < 0.05). MDD—major depressive disorder, Sub—subiculum, Hip—hippocampus, Flu—fluoxetine, PFC—prefrontal cortex.

**Figure 10:**
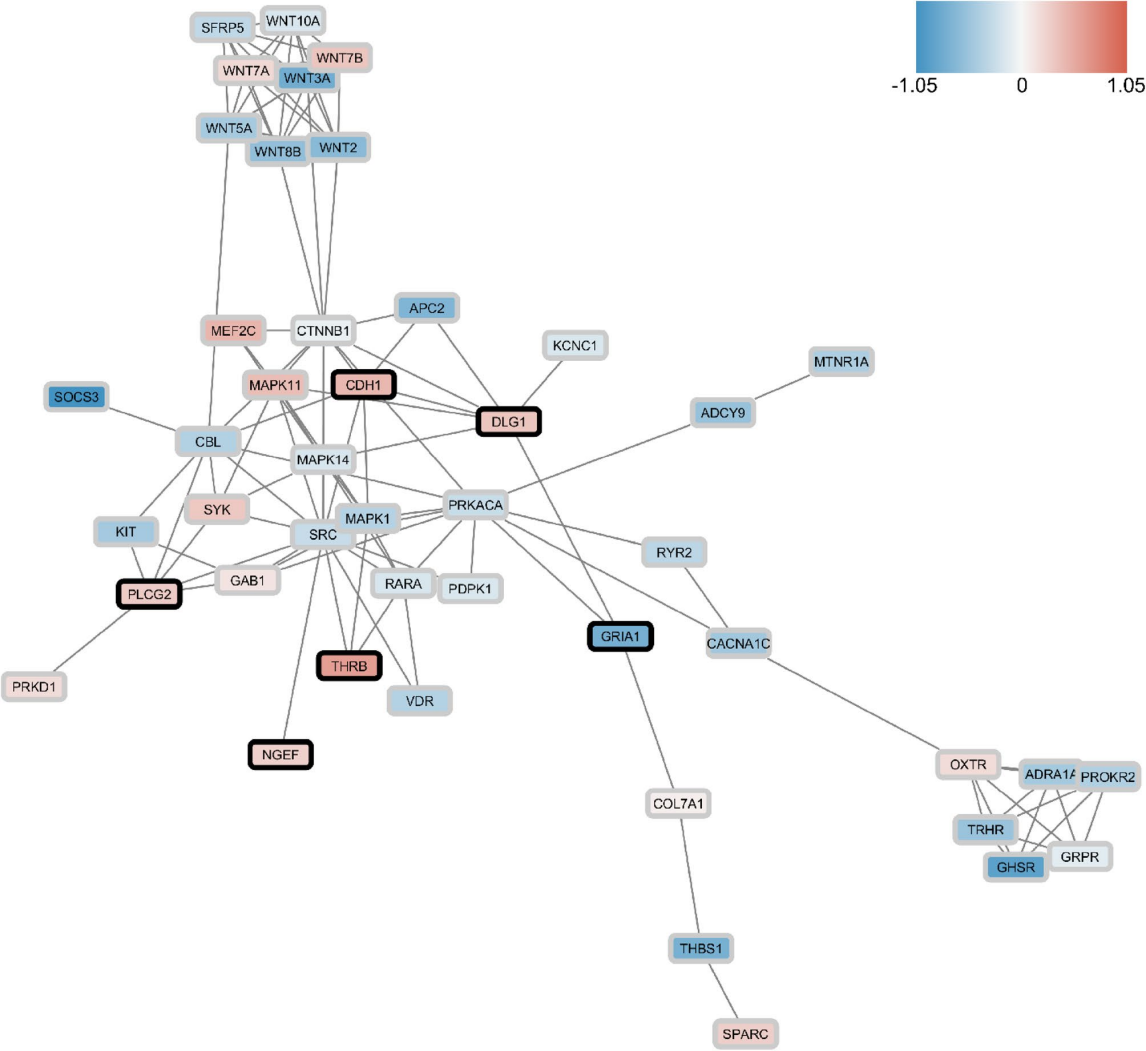
The network of protein–protein interactions (PPI) constructed for genes expressed in a similar direction in the enriched sets using Fisher’s meta-analysis^57^ on the maximal cliques (MCs) Gene Set Enrichment Analysis (GSEA) data of major depressive disorder (MDD) vs. human control or chronic unpredictable stress (CUS) vs. rat or zebrafish control data, mapped to 1:1:1 human orthologue using HomoloGene database^59^ (Experiment 4; www.ncbi.nlm.nih.gov/homologene). PPIs were constructed using the STRING online database^62^ (see the “Methods” section and Figs. 3, 5 for details; www.string-db.org). Data are presented as mean log2 fold change between the stress groups from three species, compared to their respective controls. Black frames denote genes most highly-ranked as differentially expressed (DE) in all three species (see the “Methods” section for details).

**Figure 11:**
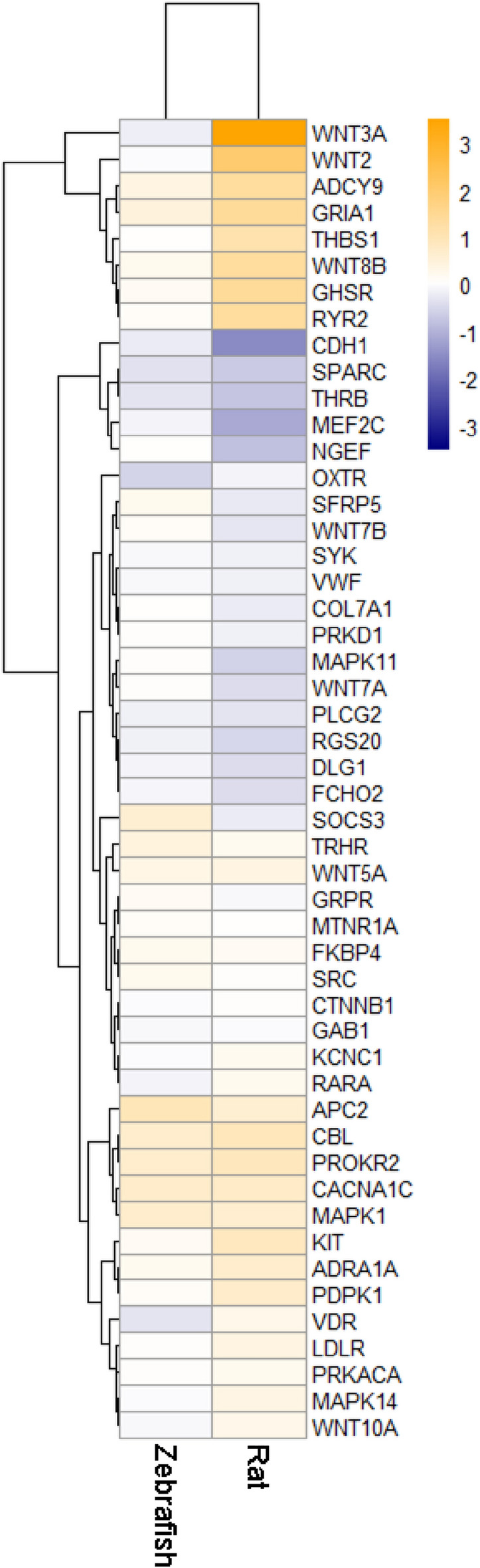
Heatmap representing fluoxetine effects in animals on brain genes expressed in a similar direction in the sets deemed ‘enriched' using Fisher’s meta-analysis^57^ on maximal cliques (MCs) Gene Set Enrichment Analysis (GSEA) data for major depressive disorder (MDD) vs. human control or chronic unpredictable stress (CUS) vs. control rat and zebrafish data, mapped to 1:1:1 human orthologue using the HomoloGene database^59^ (Experiment 4; www.ncbi.nlm.nih.gov/homologene). Data are presented as mean log2 fold change.

To identify the most consistent/stable expression changes across the three species, our further analyses focused on genes from the set with similar changes in expression (increased or decreased) for each species (Fig. 9). In silico Experiment 4 generated a PPI network consisting of a total of 45 proteins, including those encoded by 6 genes (*GRIA1, DLG1, CDH1, THRB, PLCG2,* and *NGEF*) most highly altered (assessed by average l2fc absolute values) in all three species (Fig. 10). Five of these genes (except *NGEF*) were also highly ranked by their l2fc absolute values in fluoxetine-treated zebrafish and rats, further implicating them in both stress pathogenesis and antidepressant effects (Fig. 11). Finally, our graph theory-based analyses of protein-protein interaction (PPI) networks, performed similarly to Experiment 3, helped establish multiple ‘hub’ proteins (Table 1) that may also represent promising targets due to their high impact on the PPI. Overall, these PPI analyses reveal the potential role of the Wnt-signaling pathway, involving multiple wnt proteins (e.g., WNT2, WNT3A, WNT7A and B, WNT8B, WNT10A), a protein with highly altered expression of the corresponding gene among all three species found in Experiment 4 (GRIA1), key hormone receptors (TRHR and OXTR), and some other important cellular proteins (Table 1).

## Discussion

The present study was the first cross-species/cross-taxon in-depth analysis of stress-related CNS transcriptomic data from three important organisms (humans, rats and zebrafish), in order to probe their putative shared genomic mechanisms in affective pathogenesis. This study also combined several innovative methods of analyses (Experiments 2–4) to tackle this problem, contrasting these approaches with more common and traditional, *direct* species-to-species comparisons (Experiment 1). Refining such analyses, Experiments 3 and 4 focused on mapping shared orthologous human, rat, and zebrafish genes before differential expression analyses, and were also further reinforced by meta-analytical methods, becoming more sensitive among all approaches used here. In summary, the results of our analyses are as follows: (1) The use of gene sets-related analysis is highly beneficial to study commonalities in interspecies RNA-seq results relevant to stress-related CNS disorders. (2) Data from different species cannot be directly pooled together due to high heterogeneity of their gene expression. (3) The meta-analytical approach is highly efficient in combining the interspecies RNA-seq results. (4) The value of analyzing gene sets may be increased by using functionally meaningful ways of data extraction, such as finding MCs in the targeted PPI networks.

Interestingly, some potential protein targets that were up-regulated and had high connectedness in PPI networks generated by Experiment 3, include the FEZ Family Zinc Finger 2 (FEZF2) and IKAROS Family Zinc Finger 1 (IKZF1), both having zing-finger Cys_2_His_2_-like fold group (zf-C2H2)^68^ and 36.67-% homology to each other, based on protein sequences assessed by the Basic Local Alignment Search Tool (BLAST) database^69^. In neurons, both FEZF2 and IKZF1 are important TFs that determine neuroprogenitor cell fate^70^. FEZF2 also promotes neuroplasticity and neuronal signaling that involves neuroactive ligand-receptor interaction, cell adhesion molecules, and calcium signaling pathways^71^, thereby strikingly paralleling our KEGG ^56^ pathways enrichment findings here (Fig. 2 and Supplementary Tables S4–S6 and S10). FEZF2 also controls the expression of Helix-loop-helix (HLH) DNA-binding domain-containing proteins^72,73^, such as neurogenins, neurogenic differentiation (NEUROD), and ASCL1 orthologues that represent closely related HLH proteins controlling neuronal fate (e.g., temporal switch from neuro- to gliogenesis)^68,70,74^. Interestingly, *NEUROD6* was a DE gene found by meta-analysis in the present study, albeit altered in different directions across species (Fig. 6). Similarly, IKZF1 participates in neuronal differentiation, including the differentiation of the growth hormone releasing hormone (GHRH) cells in mammalian hypothalamus^70^, and its genetic knockout in mice evokes pronounced antidepressant-like behavior and poorer acoustic startle^75^. Collectively, this corroborates our present findings identifying this gene as a potential critical ‘shared', evolutionarily conserved candidate CNS ‘affective’ gene (Fig. 6). Notably, *IKZF1* is more robustly expressed in microglia than in neurons, oligodendrocytes or astrocytes, as assessed by mean DE TFs in human cortex^76^. Furthermore, comparison of meta- and TFBS- analyses with this patterned expression data^76^ suggests that most DE genes and DR TFBSs (Experiments 1 and 3) after stress exposure may be associated with primarily microglial (e.g., *IKZF1, PRDM1, ELF1, ELK3, ETS2,* and *FLI1*), and with only a few neuronal (e.g., NEUROD6) and astrocytic (e.g., FEZF2), genes^76^.

Overall, the observed diversity of the cell types implicated in depression is not surprising, as microglia, astrocytes, endothelial cells and oligodendrocytes have been extensively studied for their association with affective pathogenesis. For example, microglial cells emerge as promising novel targets for depression treatment, representing important modulators of inflammatory activity in the brain, also key for supporting healthy neuronal connectivity^24^. Moreover, endothelial dysfunction biomarkers are associated with depression pathogenesis clinically, and are normalized following antidepressant treatment^77^. Similarly, astrocytes regulate glucose metabolism, neurotransmitter uptake (especially glutamate), synaptic development/maturation or the blood brain barrier function, and their role in depression pathogenesis is well supported by clinical and preclinical studies^78,79^. Finally, postmortem reduction of glial density in amygdala (an important regulator of emotional responses) may be primarily due to oligodendrocyte cell death^80^, whereas of the down-regulation of oligodendrocytal genes is observed in both depressed patients and animal chronic stress models^81^. Taken together, the present findings are in line with recent views on depressive pathogenesis implicating multiple brain cell types, and supporting the value of studying whole-brain tissue in addition to cell type-specific samples.

The present study also compared all candidate genes found in meta- and TFBS- analyses with publicly available meta-analyses of cell-specific expression patterns in human and mouse brain^82^. Several observations can be made based on these analyses. First, as shown in Supplementary Fig. 1, all brain cell types were involved in the development of pathological affective states in all three species here. Second, all neuron-specific genes were altered bidirectionally between these species, confirming some distinct effects of chronic stress on neuronal tissue in various vertebrates reported previously^83^. Finally, some key microglial (e.g., IKZF1) and astrocytal (e.g., FEZF2) proteins may play an integrative, ‘hub’ role in stress-related ‘affective’ PPI networks generated here (Table 1).

However, the present study also has several clear limitations. First, the Fisher’s meta-analysis^57^ was oversensitive to highly-DE genes in individual species (e.g., *FEZF2* in rats), thus possibly not properly reflecting its potential evolutional conservation, as suggested by other methods used here. Furthermore, our study utilized zebrafish whole brain samples, rat hippocampal samples and online human subiculum data, hence possibly complicating direct inter-species comparisons. Likewise, only male rat and human data, but mixed-sex zebrafish data, were analyzed. However, rat and human data were as close to each other as to zebrafish data in terms of the number of identified conservative genes, sets, TFBS or principal components (Experiment 2), thus supporting the validity of using zebrafish whole-brain and mixed-sex samples in the pilot analysis here.

Importantly, as a proof of concept, in a separate study we also utilized depression patient PFC data to assess the enrichment of the set of 253 genes found here to be altered in all three species (hippocampus in rats and human and whole brain in zebrafish) in Experiment 4. Overall, these analyses yielded pronounced enrichment in depressed vs. healthy patients (Fig. 9), thereby supporting the idea of targeting the effects common in other brain regions. Similarly, an antidepressant exherted opposite effects on the gene set enrichment (Fig. 9) and expression of genes of interest (Fig. 11) in Experiment 4, thus providing further pharmacological validity for the study. However, as already noted, variation in brain regions may also affect the study results, for example, contributing to the lack of common DE genes between species using traditional species-to-species analyses in in Experiment 1. Thus, further follow-up studies may be needed to generate more nuanced insights by focusing on sex- and brain area-specific samples.

Finally, using the HomoloGene database^59^ (www.ncbi.nlm.nih.gov/homologene) to identify gene orthologues across species also presents some limitations because it is currently incomplete, and not all existing gene orthologues are registered there. For example, the *IKZF1* orthologue was not identified in the rat genome, albeit the *Ikzf1* gene exists in rats and is highly homological to the respective human and zebrafish genes, as assessed by the BLAST database^69^. As such, using a curated database of gene orthologues to simplify their identification may also lead to false negatives (e.g., yielding fewer genes due to data deficiency), hence necessitating further manual data curation and updating. Moreover, because teleost fish underwent an additional round of whole-genome duplication, many of the mammalian genes have additional orthologues in zebrafish, resulting in over-representation of zebrafish over human and rat genomes. To mitigate this potential confound, the present study used only one (most homological) zebrafish ortholog for each such duplicated gene. However, this strategy may also impact the results of the study since some of the two gene orthologues may hypothetically have divergent CNS functions, brain localization and/or expression patterns.

Another important aspect to consider is the overall validity of animal modelling for human brain disorders. The translational relevance of animal models of depression has traditionally been evaluated based on their predictive, construct and face validity^84^. It is generally accepted that CUS paradigms in rodents fulfill all these criteria, since they replicate many symptoms of depression seen in humans (good face validity), show specific and selective responses to antidepressants (good predictive validity), and have sound theoretical basis (good construct validity)^85,86^. However, as with other animal models of depression, CUS-based paradigms themselves have several important conceptual and methodological limitations. In fact, while stress is one of the key predictors for depression development in humans^87^, the exact cause-effect relationships between stress and depression are poorly understood. For instance, it is still unclear how stressful events cause pathological changes in the brain of depressed patients, and why some other individuals remain stress-resistant or stress-resilient^88^. Furthermore, some symptoms of depression cannot in principle be modeled in animals, either due to their high cognitive complexity (e.g., suicidality) or inability of animals to objectively report their internal states (e.g., feelings of worthlessness and guilt).

Depression is also a highly heterogeneous disorder in terms of its clinical manifestations^89,90^, which further complicates adequate studying its symptoms and their modeling in animals. For example, while dysregulated neuroendocrine axis is often the most consistent physiological sign of depression, it actually occurs only in ~ 50% depressed patients^91^. Given the high comorbidity between depression and various other affective disorders (especially anxiety)^92^, it is also unclear whether they represent trully distinct brain disorders or diverse manifestations of some common overlapping pathological process.

As already mentioned, Experiments 1–3 were more sensitive in identifying similar changes in pathways expression than analyses of expression changes in individual genes. However, there are no pathways in the KEGG database^56^ that have a direct pathological association with CNS affective pathogenesis, since exact molecular pathways that contribute to these disorders remain poorly understood. Therefore, it is logical to specifically focus on the most affected gene sets in all three species observed in the study independently of curated pathways. To address this problem, the present study utilized a novel approach, comparing differences in expression data of MCs identified in orthologous PPI networks. Although MC analyses have already been used in various transcriptomic studies^93–96^, here we not only applied this approach to CNS transcriptomic data across the three common model species, but also enhanced its sensitivity, successfully identifying multiple enriched MCs by combining this approach with meta-analytical methods.

Assessing genes that formed significantly altered MCs, we also identified a novel gene set that may be potentially useful for comparing animal and human affective pathological states (used as a curated pathway in databases). For example, this set includes several well-known stress- and affective disorder-related genes, such as FOS-, JUN-, MAPK-, Wnt- and adhesion-related genes, thereby further supporting the validity of our approach. Importantly, we also identified the most conservative ‘stress’ subnetwork consisting of CNS genes that were not only inter-connected (within PPIs), but also changed their expression in the same direction in all three species studied here (Fig. 9, 10, Supplementary Table S14). For example, this subnetwork includes several similarly expressed “core” genes in all three stressed groups (*GRIA1, DLG1, CDH1, THRB, NGEF,* and *PLCG2*), likely serving as potential ‘hub’ genes within the subnetwork (Tables 1, 2).

**Table 2:** A brief summary of genes identified as ‘core’ (‘hub’) in the present study.

Genes	Link to affective pathogenesis	Cellular localization	Brief description
*CDH1*	+ + +	Cell membrane	E-cadherin, involved in adhesion junction and wnt-signaling. Interacts with beta-catenin that is highly associated with affective pathology and anxiety clinically and in animal models. Expression is modulated with ketamine exposure
*GRIA1*	+ + +	Postsynaptic membrane	Glutamate ionotropic receptor α-amino-3-hydroxy-5-methyl-4-isoxazolepropionic acid (AMPA) type subunit 1, associated with affective pathology and anxiety both clinically and in animal models
*DLG1*	+ + +	Cytosol	Scaffold protein regulating glutamate receptors activity
*IKZF1*	+ +	Nucleus	Transcription factor regulating neuronal progenitors’ fate. Is associated with depressive phenotype in animal models
*FEZF2*	+ +	Nucleus	Transcription factor regulating neurogenesis and neuroplasticity
*THRB*	+ +	Nucleus	Nuclear receptor to thyroid hormone whose dysfunction is associated with affective pathology
*PLCG2*	+	Cytosol and extracellular plasma	Important metabolic protein involved in transmembrane signaling, associated with inflammation and neurological diseases
*NGEF*	+	Cytosol, nucleus	Understudied protein, associated with mitogen-activated protein kinase (MAPK) signaling and cell junction

Evidence corresponds to subjective authors’ relative evaluation of genes involved in affective pathology using study results and literature analysis (see the “Discussion” section for details).

Thus, our analyses successfully identified shared genes that were involved in affective pathology in all three species, hence representing likely evolutionarily conserved biomarkers of affective pathology. Furthermore, the expression of all genes similarly expressed across all three species, except *NGEF*, was rescued in the respective rat- and zebrafish fluoxetine-treated groups, further implicating their importance not only for CNS stress responses, but also for antidepressant treatment, again strongly supporting the validity of the present study’s approach and findings.

*PLCG2* belongs to the phospholipase C gamma (PLC) family, encoding the enzyme 1-phosphatidylinositol-4,5-bisphosphate phosphodiesterase gamma-2 (PLCG2) that cleaves the membrane phospholipid PIP2 (1-phosphatidyl-1D-myo-inositol 4,5-bisphosphate) to the second messengers IP_3_ (myoinositol 1,4, 5-triphosphate) and DAG (diacylglycerol) playing a key role in signal transduction^97–99^. Another PLC family member, *PLCG1* is expressed widely within the brain, especially in the cortex and the hippocampus^100^, and has been implicated in CNS disorders, such as epilepsy, Huntington's disease, and bipolar and unipolar depression^101–103^. In contrast, *PLCG2* is predominantly expressed in the bone marrow and lymphoid organs^104^ and is responsible for hereditary immune and autoimmune disorders^105,106^. However, recent mouse studies found *PLCG2* expression in the granular cell layer of the dentate gyrus and microglia^107^, and its mutations are also implicated in Alzheimer's disease^107^. Together with our present findings, thus implicates both PLCG1 and PLCG2 as potential factors in both neurodegenerative and affective disorders.

The *THRB* gene generates two alternatively spliced isoforms of the thyroid hormone receptor beta, TRβ1, and TRβ2^108^. Together with another receptor, TRα, these nuclear receptors act as transcription factors that mediate the genomic effects of thyroid hormone in various tissues^109,110^. Importantly, while thyroid receptors are highly expressed in the brain^111,112^, thyroid hormones (e.g., acting via brain TRα and TRβ receptors) may modulate monoaminergic neurotransmission, thereby affecting mood and behavior^113–116^, including strong co-morbidity of thyroid dysfunctions with mood disorders^117–119^.

The *NGEF* (Neuronal Guanine Nucleotide Exchange Factor) gene^120^ is expressed in the caudate nucleus and is involved in the activation of RhoA, Rac1, and Cdc42 (the Ras superfamily-associated proteins), hence modulating mitogen-activated protein kinase (MAPK) signaling and cell junction^121^. NGEF is also a downstream signaling component of the ephrin-A (EphA4) tyrosine kinase receptor, responsible for the formation of neural networks, nerve growth, and changes in cell morphology involved in cell motility^122–126^. Some genetic studies also implicate NGEF in schizophrenia^127,128^. However, the CNS functions of *NGEF* remain poorly understood, and its putative role in stress-related affective pathogenesis (as suggested in the present study) merits further study.

The *GRIA1* gene encodes the GluR1 (GluA1) protein, a subunit of glutamate α-amino-3-hydroxy-5-methyl-4-isoxazolepropionic acid (AMPA) receptor, critical for synaptic plasticity, learning, and memory^129–131^. *GRIA1* is ubiquitously expressed throughout the rat and human brain, with the highest expression in the hippocampus^132,133^. Interestingly, *GRIA1*-/-knockout mice display hyperlocomotion, increased anxiety, exacerbated novelty response, and impaired spatial working memory and object recognition^134–138^. Mounting evidence implicates GluR1 dysregulation in uni- and bipolar depression, and schizophrenia ^139–141^, hence calling for further studies of its role in stress in vivo. Rodent GluR1 activity alters in various brain areas following both acute^142–145^ and chronic stress^143,146–154^, with usually downregulated *GRIA1*/GluR1 expression/protein content. However, patterns of GluR1 regulation depend on both the duration of stress and the specific brain area examined. For example, hippocampal GluR1 is upregulated after shorter-term (< 21 days)^143,149,150^, but downregulated under longer-term (> 28 days), chronic stress^151,153,154^. Furthermore, treatment with classical antidepressants (e.g., fluoxetine, desipramine, and maprotiline), as well as with atypical fast-acting antidepressant ketamine, elevates *GRIA1* expression and restores GluR1 level in chronically stressed rodents^155–159^. Together with our present cross-species genomic findings (Figs. 9, 10, and Supplementary Table S14), this strongly suggests GluR1 as a ‘core’, an evolutionarily conserved gene involved in affective pathogenesis.

Discs large homolog 1 (DLG1) is a scaffolding protein from the membrane-associated guanylate kinase (MAGUK) family that regulates the activation of both B- and T-lymphocytes, encoded in humans by the *DLG1* gene^160,161^. Rat Dlg1 localizes in the presynaptic nerve endings of excitatory synapses, as well as in (and along with) the bundles of unmyelinated axons^162^. In addition to mammalian DLG1, there are several other types of DLGs (DLG2 (PSD-93), DLG3 (NE-dlg), and DLG4 (PSD-95)) expressed almost exclusively in the nervous system^163^ and likely contributing to rodent affective pathogenesis (e.g., Dlg4, but not Dlg1 or Dlg2, decreases in mouse hippocampus two days after the forces swim test^164^). DLG1 also regulates the activity of the glutamate N-methyl-D-aspartic acid (NMDA) and AMPA receptors^165,166^. NMDA receptor antagonists like ketamine exert both anxiolytic and antidepressant properties^167^, whereas antagonism of AMPA receptors is linked to depression^168^.

Perhaps the most interesting finding here (Table 2) in the context of affective pathology involves Epithelial-cadherin (E-cadherin; *CDH1*), a Ca^2+^-dependent cell adhesion molecule^169^, whose extracellular region acts as an adhesion anchor binding to cadherins on other cells^170^, and intracellular region interacts with catenins (e.g., α- and β-catenins) and other regulatory proteins^171^. Since β-catenin is an important signaling protein in the Wnt-signaling pathway, the cadherin/catenin complex modulates cellular signal transduction^172^. Wnt signaling has long been associated with affective pathogenesis^173^ and downregulated E-cadherin expression has been reported in vitro by ketamine, a novel rapid-acting antidepressant^174^. Similarly, a classical antidepressant, fluoxetine, impairs CDH1-mediated cell adhesion^175^. Unlike other genes, *CDH1* is overexpressed in PFC of human MDD patients vs. controls (assessed using protocol similar to Experiment 4 here), supporting its important interspecies and inter-tissue (including different brain regions) conservation.

In conclusion, translational multidisciplinary approaches remain a cornerstone for innovative CNS research. Here, we applied several novel analyses aiming to reveal evolutionarily conserved transcriptomic phenotypes across three different vertebrate animal models (zebrafish, rat and human clinical data). Using these approaches, we identified GRIA1, DLG1, CDH1, THRB, PLCG2, NGEF, IKZF1, FEZF2 as promising and shared affective ‘hub’ targets (Table 2), whose further experimental studies may markedly foster translational research of affective disorders.

## Methods

### Animals, housing, and chronic unpredictable stress modeling

Wild-type adult zebrafish (n = 6, 1:1 sex ratio) and Wistar male rats (n = 3; NCBI's Gene Expression Omnibus (GEO)^55^ accession number GSE205325 www.ncbi.nlm.nih.gov/geo/query/acc.cgi?acc=GSE205325) were subjected to the CUS protocols reported elsewhere^32,176^, utilizing the 5-week (zebrafish) or 12-week (rats) protocols. Behavioral studies reconfirmed the evoked anxiety- and depression-like phenotypes in both species^32,176^ induced by CUS. All animals were kept in standard conditions, according to national and institutional guidelines^32,176^. Additional statements regarding ethical data use are available in Ethical Confirmation statements section and in the original published studies^32,176^.

### Human subjects

Human transcriptomic data were obtained from the open source^177^ (NCBI's Gene Expression Omnibus (GEO)^55^ accession number GSE102556 www.ncbi.nlm.nih.gov/geo/query/acc.cgi?acc=GSE102556), the Douglas Bell Canada Brain Bank (DBCBQ, Douglas Mental Health Institute, Verdun, Québec). The subjects (males, n = 3 for subiculum, n = 4 for PFC study, average age 45) were of European ancestry and French-Canadian descent who died suddenly, without prolonged agony^177^. Diagnoses were obtained using Diagnostic and Statistical Manual of Mental Disorders-IV (DSM-IV) criteria using the Structured Clinical Interview for DSM-IV Axis I Disorders (SCID-I) interviews^178^ adapted for psychological autopsies^177^. While the original study^177^ included numerous suicide attempters in the control group, and many samples were from patients treated with antidepressants, we excluded these samples from the present in-silico analyses (see Statistical analyses for details), aiming at more homogenous groups. All methods involving human subjects in the cited study were carried out in accordance with relevant guidelines and regulations, as well as in accordance with the Declaration of Helsinki. Additional statements regarding ethical data use are available in Ethical Confirmation statements section and in the original study^177^.

### RNA-sequencing

RNA-sequencing procedures were performed as reported previously^32,176,177^. Briefly, animal brains were dissected on ice following standard procedures, and hippocampus were dissected from the whole rat brains using Waxholm Space atlas^179^. RNA isolation was performed using the TRI-reagent (Evrogen JSC, Moscow, Russia), according to manufacturer instructions. RNA quality was examined using Quantus (Promega Corporation, Madison, USA), electrophoresis, and QIAxcel (QIAGEN, Venlo, Netherlands). Sequencing was performed on Illumina HiSeq2500 (Illumina Inc., San Diego, USA) with 140 bp paired-read (zebrafish) and Illumina HiSeq4000 (Illumina Inc., San Diego, USA) with 151 bp paired-read (rat), with at least 20 million reads generated for each sample. Human samples from the referenced study^177^ involved dorsolateral PFC (BA8/9; dlPFC) and ventral subiculum (vSUB) carefully dissected at 4 °C after being flash-frozen in isopentane at − 80 °C by highly trained histopathologists using reference neuroanatomical maps^177,180,181^. Similar to our rat and fish experiments, RNA isolation in clinical samples was performed with TRI-reagent, according to manufacturer instructions^177^. Samples were sequenced at 50 bp paired-read on Illumina HiSeq2500 with at least 50 million reads per sample after two sequential sequencing^177^.

### Statistical analyses and data handling

Data on human RNA-seq postmortem subiculum and PFC expression were obtained from the NCBI's Gene Expression Omnibus (GEO)^55^ accession number GSE102556 http://www.ncbi.nlm.nih.gov/geo/query/acc.cgi?acc=GSE102556. Only male control and MDD patients that did not receive any treatment and died from natural or accident causes were included in the present analyses, resulting in n = 3 for subiculum and n = 4 for PFC. Reads were mapped to zebrafish GRCz11, rat Rnor_6.0, and human GRCh38 using RNA STAR^182^ and further processed in featureCounts^183^ (https://usegalaxy.org/)^184^. For inter-species transcriptomic data comparison, we applied four different approaches (summarized in Fig. 1) utilizing the R software^185^, Bioconductor software^186^, and the DESeq2 package^187^. The DESeq2 was chosen as a tool efficient for experiments with 12 or fewer replicates, stable within 0.5 fold-change thresholds, and as an approach consistent with other tools, such as EdgeR (when using exact test), Limma, and EBSeq^32,188^. All genes with less than 10 counts per all samples were removed from the analysis. DE gene data analyses were next performed using the DESeq function. The p-values were adjusted using the Benjamini–Hochberg correction^189^. *P*-value and false discovery rate (FDR) were set at 0.05 in all analyses here.

GSEA is a widely used method to study gene expression data in terms of molecular sets, allowing for better detection of expression changes^190–193^. However, classical GSEA has some limitations, including the inability to handle datasets of different sizes and some experimental designs^60^. A sub-type of GSEA, GAGE for the set analysis addresses these limitations^60^, also enabling to choose independent databases to be analyzed depending on research goals, and consistently outperforming classical GSEA methods^60^. The KEGG^56^ (www.genome.jp/kegg/) pathway enrichment analyses were performed on normalized and log2-transformed counts by the GAGE package^60^, using two-sample Student’s t-test for group comparison of differential expression of gene sets. Both up- and downregulated, as well as bidirectionally (using absolute values) altered pathways were analyzed here. The p-values were adjusted using the Benjamini–Hochberg correction^189^, with the FDR cut-off set at 0.05.

In Experiment 1, we compared DE and enriched gene sets for each species separately. Briefly, the resultant gene expression counts mapped to zebrafish GRCz11 (n = 6), rat Rnor_6.0 (n = 3) and human GRCh38 (n = 3) were analyzed independently using DESeq2 and GAGE functions comparing stress or MDD to control groups for each species (*p* adjusted < 0.05, Fig. 1). Lists of significantly DE genes in these species were further compared by searching orthologues using the HomoloGene function^59^ (http://www.ncbi.nlm.nih.gov/homologene) and Venn diagrams. Lists of significantly altered GAGE sets were also compared using their KEGG^56^ names. Finally, we studied TFBS over- and under-represented in genes with high variability (p values < 0.01 in DE analysis) vs. low variability (*p* > 0.7) using the CiiiDer TFMs software (http://www.ciiider.com/) for each species^61^ (only TFBSs with *p* < 0.05 simultaneously for gene coverage *p*-value and for the distribution of the number of TFBS were considered statistically significant). The potential binding sites were established using position frequencies matrices from the Jaspar 2020 core vertebrates matrix^194^ (https://jaspar.genereg.net/) and searched in the genomes of the respective species targeting 1500 bp upstream and 500 bp downstream of the specific genes ^61^. The resulting lists of DR binding sites were similarly compared between the species.

In Experiment 2, we performed interspecies comparison of stress (CUS/MDD) versus control effects using raw RNA-seq data counts mapped to human orthologues pooled in combined affective disorder groups (CUS + MDD) or in combined control group (animal control + healthy patients) prior to any differential analysis, aiming to achieve better inter-species data compliance (Fig. 1). Because all hypotheses in this experiment closely resembled each other (i.e., probing the effects of affective pathology on CNS transcriptome in vertebrates), we combined all RNA-seq data in one experiment, designing it as a study of affective pathology effects on human transcriptome orthologues in vertebrates. Briefly, all counts were mapped 1:1:1 to human orthologues using the HomoloGene^59^ database (http://www.ncbi.nlm.nih.gov/homologene), resulting in 10353 genes and 24 samples (n = 12) which were next assessed for stress (and MDD) vs. control group effects using the DESeq2 and GAGE analyses (*p* adjusted < 0.05).

Experiment 3 used cross-species comparison of stress/MDD vs. control samples using counts mapped to human orthologues in separate DE and GSEA analyses for each species, that were further compared using the meta-analysis (Fig. 1). Again, because hypotheses from DE and GSEA analyses in different species were similar (i.e., probing the effects of affective pathology on transcriptome in human orthologues), it was possible to compare different species using meta-analysis. Briefly, DESEq2 analysis was conducted for each species data separately, using only the genes successfully mapped 1:1:1 to human orthologues (similar to Experiment 2). The resulting 3 DESeq2 analyses were next meta-analyzed using the Fisher’s method (that utilizes one-sided p-values combination)^57^ and the metaRNASeq package in R^195^. Significantly altered genes (Benjamini–Hochberg correction *p* adjusted value < 0.05), as assessed by meta-analyses, were further selected based on their consistent unidirectional expression changes across all three species. Overall, the Experiment 3 design closely resembled RNA-seq meta-analysis in other biological studies (e.g., salt stress–responsive genes and pathways in microalga *Dunaliella*^196^), hence supporting the efficiency of cross-species meta-analyses of orthologues expression to identify evolutionarily conserved “core” changes.

We also generated a human PPI network in the STRING database (https://www.string-db.org/) for *Homo* *sapiens*^62^ using TFBSs enriched in Experiment 1 and DE genes from Experiment 3, resulting in 74 proteins connected to any other protein, with the largest network consisting of 67 vertices. We used a minimal interaction score of 0.15 and use all active interaction sources, except text-mining, to construct the network in the STRING database. The network was further processed in the CytoScape software^197^ (https://cytoscape.org/) and the CytoHubba^198^ packages to target ‘hub’ genes, using vertices’ degree^63^, bottleneck^64^, and DMNC||MNC^65^ approaches. Among proteins and TFBSs ranked as top 10 hub vertices by any of these approaches, we excluded those showing different l2fc directions in meta-analysis, to focus only on conserved hubs that cause similar effects in all species studied.

The MC data in transcriptomic analyses is widely used in biomedicine^93–96^. Here, we performed an interspecies comparison of MC enrichment in stress/MDD versus control PPI networks using the OmicsNet (https://www.omicsnet.ca/) ^67^ and STRING databases^62^ from counts mapped to human orthologues in three different experiments for each species (10,353 proteins, resulting in the largest PPI network of 7933 proteins; Fig. 1). In the resultant PPI network, we identified all MCs using the Cytoscape plugin MClique^197^, finding a total of 121219 MCs (Supplementary Material 3). The majority (82%) of these MCs consisted of nodes (genes) that all originated from the same few ribosomal genes that were further excluded from analyses as non-specific, thus yielding 21938 (18%) non-ribosomal MCs of interest (Fig. 7). Ribosomal MCs were identified as cliques containing S or L Ribosomal Proteins genes (*RPS* or *RPL*) as vertices. The final 21,938 non-ribosomal MCs were further analyzed as gene sets using GAGE for each species, comparing the bidirectional expression of MC in stress versus control.

Resulting DE MCs were further compared using a meta-analysis to identify MCs with conserved expression among species, like DE genes and enriched gene sets in Experiment 3. We then used all the genes composing MCs that were significantly enriched in meta-analysis, to generate a single novel gene set (containing 253 genes) and, as a proof-of-concept, compared its expression changes as a whole using the GAGE approach in the individual species-specific (non-pooled) stress sample groups. Additionally, we also compared the expression of the same 253-gene set in human PFC, in rat CUS + fluoxetine hippocampal , and in zebrafish CUS + fluoxetine whole-brain samples vs. their corresponding stress-free controls (Fig. 8 and Supplementary Table S21). To further process these data, we excluded from the set the genes with different expression directions in the species-specific groups, compared to their respective control groups. Finally, we built a PPI network for the remaining 45 genes (45 proteins) and attempted to identify core pathology-related proteins using two additional approaches. One was similar to Experiment 3 techniques of graph analyses, identifying vertices degree^63^, bottleneck^64^, and DMNC||MNC^65^ of the vertex. Another approach ranked all vertices using l2fc of each original group, taking top 10 over- and top 10 under-expressed genes in each group, and then analyzed the lists of genes, identifying 6 genes that are stably highly over- or under-expressed across all 3 species. We further compared these gene lists for fluoxetine versus stress effects, identifying pronounced antidepressant effects on the expression of 5 of these genes, thus further corroborating our findings.

Analyses of all in vivo data in this study were performed online and offline without blinding the analysts to the treatments, since all animals and samples were included in analyses, data were analyzed in a fully unbiased automated method, and the analysts had no ability to influence the results of the experiments. The study experimental design and its description here, as well as data analysis and presenting, adhered to the Animal Research: Reporting of In Vivo Experiments (ARRIVE) guidelines for reporting animal research and the Planning Research^199^ and Experimental Procedures on Animals: Recommendations for Excellence (PREPARE) guidelines for planning animal research and testing^200^.

### The graph theory-based analyses

The graph theory-based analyses of gene expression data were performed using the Cytoscape software for biomolecular interaction networks construction and analyses version 3.8.0^197^ (https://cytoscape.org/). The PPI networks were constructed using the STRING (Search Tool for the Retrieval of Interacting Genes/Proteins) database^62^ (https://www.string-db.org/). The resultant PPI networks were analyzed by the Cytoscape application cytoHubba^198^ to probe essential vertices/hubs in PPI networks for top 10-degree^63^ vertices, top 10 bottleneck^64^ vertices, or top 10 vertices by the Double Screening Scheme (DSS), combining Density of Maximum Neighborhood Component (DMNC) and Maximum Neighborhood Component (MNC)^198^, as in^32^. The degree of the vertex *v* was defined as the number of edges of vertex *v*, thus representing the number of a protein’s connections to other proteins^63^, similar to^32^. The bottleneck vertices were determined using the betweenness centrality of the vertex, based on the measuring of the number of shortest passes going through the vertex^64^, similar to^32^. Bottleneck proteins likely represent essential ‘hubs’ in the network functioning as connectors bridge-like proteins^201^. MNC of the vertex *v* was defined as a size of the maximum connected component of subnetwork *N*(*v*) constructed by vertices adjacent to *v*^65^, similar to^32^. DMNC of a vertex v was defined as *E/N*^*ε*^ where *N* is vertex number and* E* is the edge number of MNC(*v*), and *ε* is defined as 1.7^65^. DSS was further calculated as follows: for *n* most possible essential proteins that were expected in the output (*n* is an empirical value), *2n* top-ranked proteins were selected by DMNC method^65^, similar to^32^. The selected proteins were then ranked by the MNC values, selecting top *n* proteins for analyses. The DSS (DMNC||MNC) method was chosen here as an effective tool to identify essential proteins within molecular networks^65^.

### Ethics approval

The study does not include any direct animal or human experimentation and uses previously published datasets^32,176^ from animal experiments approved by the Institutional animal care and use committee (IACUC) of St. Petersburg State University and/or the Institute of Experimental animal of Almazov National Medical Research Center, that fully adhered to the National and Institutional guidelines and regulations on animal experimentation, as well as to the 3Rs principles of humane animal experimentation. The human data used here resulted from publicly available datasets in published studies (approved by the research ethics boards of McGill University and UT Southwestern), with necessary written informed consents obtained from all participants^177^.

## Supplementary Information

Supplementary Information 1.

Supplementary Information 2.

Supplementary Information 3.

## Data Availability

The datasets generated and/or analyzed during the current study are available from the corresponding authors upon reasonable request.
