# Supplementary material for: Evolutionarily conserved gene expression patterns for affective disorders revealed using cross-species brain transcriptomic analyses in humans, rats and zebrafish
Source: Sci Rep. 2022 Dec 2;12:20836. doi: 10.1038/s41598-022-22688-x (PMC9718822; doi:10.1038/s41598-022-22688-x)
Supplement: Supplementary file 1 — Supplementary Information 1. [file 41598_2022_22688_MOESM1_ESM.pdf]

SUPPLEMENTARY ONLINE MATERIALS

Revealing translational, evolutionarily conserved molecular biomarkers of affective disorders using brain transcriptomic data from humans, rats and zebrafish

Konstantin A. Demin*[1,2], Nataliya A. Krotova[1,2], Nikita P. Ilyin[1,2,4], David S. Galstyan[2,3], Tatyana O. Kolesnikova[4], Tatyana Strekalova[5], Murilo S. de Abreu[6], Elena V. Petersen[6], Konstantin N. Zabegalov[4], and Allan V. Kalueff*[3,7,8]

[1]Almazov National Medical Research Centre, St. Petersburg, Russia

[2]Institute of Translational Biomedicine, St. Petersburg State University, St. Petersburg, Russia

[3]Laboratory of Preclinical Bioscreening, Granov Russian Research Center of Radiology and Surgical Technologies, Ministry of Healthcare of Russian Federation, Pesochny, Russia

[4]Neurobiology Program, Sirius University of Science and Technology, Sochi, Russia

[5]University of Maastricht, Maastricht, Netherlands

[6]Moscow Institute of Physics and Technologies, Moscow, Russia

[7]School of Pharmacy, Southwest University, Chongqing, China

[8]Ural Federal University, Ekaterinburg, Russia

[*]Co-corresponding authors:

Allan V. Kalueff, Ph.D., School of Pharmacy, Southwest University, Chongqing, China.

Tel/Fax: +1-240-899-9571 E-mail: avkalueff@gmail.com

Konstantin A. Demin, Institute of Translational Biomedicine, St. Petersburg State University, St. Petersburg, Russia. E-mail: deminkasci@gmail.com and k.demin@spbu.ru

Data availability

The datasets generated and/or analyzed in the present study are available from the original article, supplementary materials, or the corresponding author (upon reasonable requests, for use in collaborative research projects and/or for joint publications resulting from such projects).

Supplementary Figure S1. The network of protein–protein interactions (PPI) constructed for differentially expressed genes found in Experiment 3 or differentially represented transcription factors found in Experiment 1 using the STRING online database [S1] (see "Methods" section and Figures 2 and 4 for details). Color corresponds to a cell type with a most significant expression pattern of this gene among other cell types based on [S2] data. Green – neuron, Blue – microglia, Yellow – astrocyte, Pink – endothelium, Red – oligodendrocyte, Grey – no specificity.

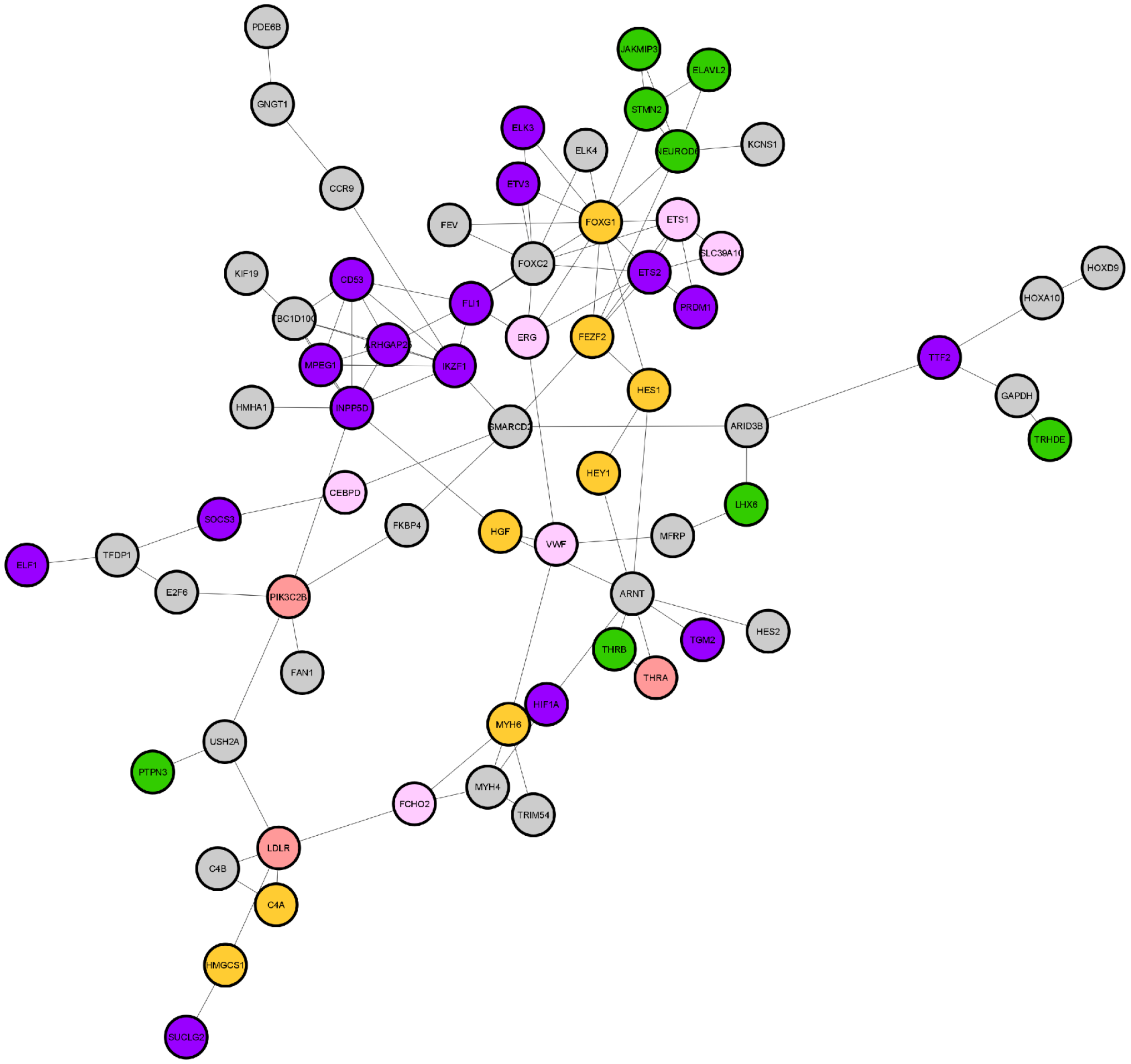

Supplementary Figure S2. All 253 genes that made up all 257 enriched sets (cliques) using Fisher's meta-analysis on maximal cliques GSEA data of MDD vs. control for human or CUS vs. control for rat and zebrafish data, mapped to 1:1:1 human orthologue using HomoloGene database (Experiment 4). Data are presented as mean log 2-fold change.

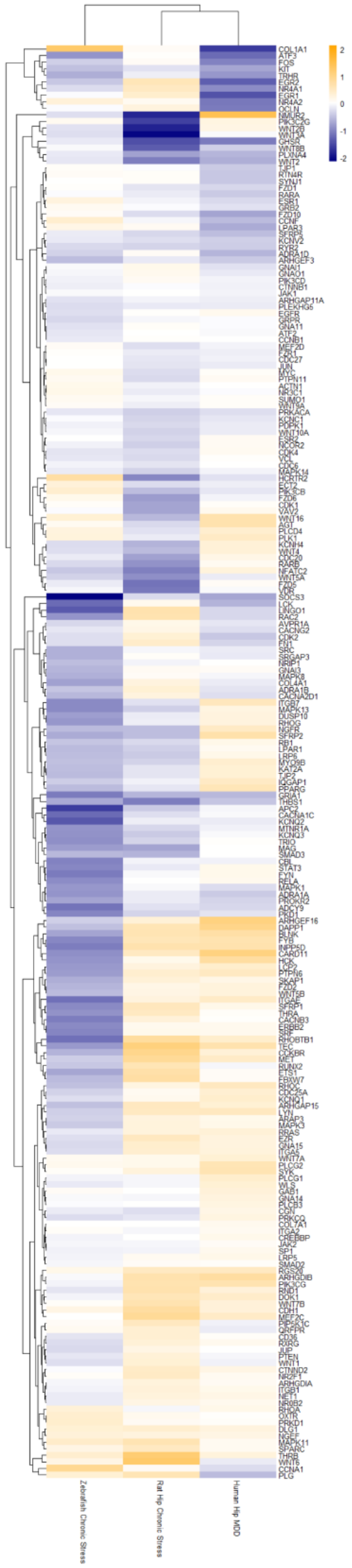

Supplementary Table S1. Summary of statistical analyses for all differentially expressed genes in subiculum of mdd vs. control groups, on the Negative Binomial (Gamma-Poisson) distribution performed by estimation of size factors, estimation of dispersion and negative binomial generalized linear models and Wald statistics using the DESeq function (Experiment 1) [S3]. P value and false discovery rate were set at 0.05. Genes in this table are listed as sorted by their p-adjusted value.

ensembl_gene_id	external_gene_name	baseMean	log2FoldChange	lfcSE	stat	pvalue	padj
ENSG00000228971	LINC02607	583.1221	-1.70525	0.296063	-5.75973	8.42E-09	0.000129
ENSG00000072657	TRHDE	2478.091	-1.39391	0.254948	-5.46741	4.57E-08	0.000351
ENSG00000206306	HLA-DRB1	119.9883	-3.13795	0.584804	-5.36581	8.06E-08	0.000413
ENSG00000228080	HLA-DRB1	119.9883	-3.13795	0.584804	-5.36581	8.06E-08	0.000413
ENSG00000196126	HLA-DRB1	119.9883	-3.13795	0.584804	-5.36581	8.06E-08	0.000413
ENSG00000229074	HLA-DRB1	119.9883	-3.13795	0.584804	-5.36581	8.06E-08	0.000413
ENSG00000206240	HLA-DRB1	119.9883	-3.13795	0.584804	-5.36581	8.06E-08	0.000413
ENSG00000236884	HLA-DRB1	119.9883	-3.13795	0.584804	-5.36581	8.06E-08	0.000413
ENSG00000107105	ELAVL2	4363.99	-1.06935	0.216867	-4.93089	8.19E-07	0.002959
ENSG00000276495	APBA2	139.1171	-2.32477	0.474534	-4.89907	9.63E-07	0.002959
ENSG00000034053	APBA2	139.1171	-2.32477	0.474534	-4.89907	9.63E-07	0.002959
ENSG00000110799	VWF	831.6272	0.799513	0.174939	4.570246	4.87E-06	0.010692
ENSG00000164600	NEUROD6	4755.992	-1.22826	0.276449	-4.44301	8.87E-06	0.017036
ENSG00000121769	FABP3	3162.147	-0.91524	0.214055	-4.27572	1.91E-05	0.032522
ENSG00000145335	SNCA	8612.551	-0.79021	0.185892	-4.25093	2.13E-05	0.032706
ENSG00000104435	STMN2	23949.2	-0.68666	0.163157	-4.20858	2.57E-05	0.0329
ENSG00000145428	RNF175	728.6949	-0.71876	0.170753	-4.20935	2.56E-05	0.0329
ENSG00000162636	FAM102B	4363.323	-0.68536	0.164664	-4.1622	3.15E-05	0.035557
ENSG00000183662	TAFA1	1539.503	-0.89774	0.216016	-4.15589	3.24E-05	0.035557
ENSG00000019505	SYT13	5645.472	-0.96998	0.234776	-4.1315	3.6E-05	0.036912
ENSG00000107295	SH3GL2	6528.436	-0.81692	0.198764	-4.11002	3.96E-05	0.037987
ENSG00000283486	FAM95C	263.9834	1.595475	0.391803	4.072141	4.66E-05	0.042097
ENSG00000196169	KIF19	86.21406	1.48732	0.371343	4.005246	6.2E-05	0.048559
ENSG00000180347	ITPRID1	133.1223	1.258958	0.314702	4.000475	6.32E-05	0.048559
ENSG00000196950	SLC39A10	8492.392	-0.59877	0.14907	-4.01672	5.9E-05	0.048559

Supplementary Table S2. Summary of statistical analyses for all differentially expressed genes in hippocampus of stressed rats vs. control groups, on the Negative Binomial (Gamma-Poisson) distribution performed by estimation of size factors, estimation of dispersion and negative binomial generalized linear models and Wald statistics using the DESeq function (Experiment 1) [S3]. P value and false discovery rate were set at 0.05. Genes in this table are listed as sorted by their p-adjusted value.

Lookup	external_gene_name	baseMean	log2FoldChange	lfcSE	stat	pvalue	padj
ENSRNOG00000007027	Hgf	402.9687	1.881954	0.277063	6.79252	1.1E-11	1.72E-07
ENSRNOG00000032063	Gfral	29.41118	-4.88717	0.771475	-6.33484	2.38E-10	1.86E-06
ENSRNOG00000009206	Fezf2	892.2903	1.00551	0.17355	5.793776	6.88E-09	3.59E-05
ENSRNOG00000007189	Ttc22	395.3506	1.654937	0.302629	5.468535	4.54E-08	0.000178
ENSRNOG00000008182	Htra3	555.3568	1.112187	0.218047	5.100679	3.38E-07	0.001059
ENSRNOG00000005565	Traf3ip3	240.6712	1.318955	0.263839	4.999088	5.76E-07	0.001502
ENSRNOG00000003237	Tp53bp2	5684.032	1.207942	0.245775	4.914829	8.89E-07	0.001809
ENSRNOG00000006850	Ovol2	48.68037	4.566898	0.931696	4.901701	9.5E-07	0.001809
ENSRNOG00000013681	Kcns1	296.1628	1.418264	0.290402	4.883797	1.04E-06	0.001809
ENSRNOG00000003738	Ush2a	53.62552	-1.88434	0.394877	-4.77197	1.82E-06	0.002855
ENSRNOG00000016553	Vwa5b1	179.2027	-2.17474	0.466476	-4.66206	3.13E-06	0.004082
ENSRNOG00000021663	Vxn	16025.48	1.219877	0.261061	4.672773	2.97E-06	0.004082
ENSRNOG00000010597	Slc5a7	297.3285	-2.71896	0.588485	-4.62027	3.83E-06	0.004613
ENSRNOG00000002930	Ppl	557.1387	-3.70883	0.81792	-4.53447	5.77E-06	0.006455
ENSRNOG00000029165	Stx1a	6547.811	1.773803	0.399162	4.443819	8.84E-06	0.009219
ENSRNOG00000001036	Rsph10b	858.3706	-1.71746	0.397489	-4.32077	1.55E-05	0.014942
ENSRNOG00000009582	Rpe65	171.7668	-1.47984	0.343251	-4.31125	1.62E-05	0.014942
ENSRNOG00000026087	Igfn1	64.18883	2.060646	0.482999	4.26636	1.99E-05	0.017273
ENSRNOG00000006674	Rflnb	162.487	-1.13819	0.267591	-4.25349	2.1E-05	0.017333
ENSRNOG00000046001	AABR07030823.1	361.3258	-1.92709	0.454677	-4.23837	2.25E-05	0.017615
ENSRNOG00000016069	Cd3e	355.1246	-7.42458	1.806011	-4.11104	3.94E-05	0.028016
ENSRNOG00000046276	Myh3	45.12269	2.764341	0.672209	4.112322	3.92E-05	0.028016
ENSRNOG00000037196	Spag17	35.65181	-3.14672	0.767816	-4.09827	4.16E-05	0.028319
ENSRNOG00000055858	Myb	46.15645	-1.99927	0.489529	-4.08407	4.43E-05	0.028853
ENSRNOG00000016552	Hmgcs1	19742.62	0.758725	0.186912	4.059259	4.92E-05	0.030813
ENSRNOG00000019317	Cplx3	290.6864	2.425395	0.603178	4.021028	5.79E-05	0.032712
ENSRNOG00000025848	Sspo	118.0201	-4.26712	1.06183	-4.01865	5.85E-05	0.032712
ENSRNOG00000030016	Robo3	786.2084	-3.018	0.747903	-4.03529	5.45E-05	0.032712
ENSRNOG00000009946	Ldlr	1374.697	-0.76938	0.192215	-4.0027	6.26E-05	0.033574
ENSRNOG00000029938	Pik3c2b	911.4091	-0.82297	0.205937	-3.9962	6.44E-05	0.033574
ENSRNOG00000039107	Mfrp	139.0656	-3.26553	0.821798	-3.97364	7.08E-05	0.035729
ENSRNOG00000001346	Cops6	9958.779	0.636455	0.162577	3.914801	9.05E-05	0.041554
ENSRNOG00000006649	Thrb	2444.081	1.317001	0.335973	3.919956	8.86E-05	0.041554
ENSRNOG00000011824	Trh	247.3715	-2.67136	0.683507	-3.90831	9.29E-05	0.041554
ENSRNOG00000020485	Vav3	1317.465	-1.86878	0.475956	-3.92637	8.62E-05	0.041554
ENSRNOG00000026091	Slc10a4	98.70734	-2.86075	0.733925	-3.89787	9.7E-05	0.04218
ENSRNOG00000004889	Vangl2	360.6807	-1.16871	0.304594	-3.83695	0.000125	0.044303
ENSRNOG00000006146	Trim54	167.1473	2.737984	0.709031	3.861584	0.000113	0.044303
ENSRNOG00000006970	Ntn3	269.1672	0.939737	0.244728	3.839927	0.000123	0.044303

ENSRNOG00000017659	Hs3st2	401.2136	1.785493	0.465072	3.839176	0.000123	0.044303
ENSRNOG00000033261	Fam107a	39655.42	0.959723	0.250035	3.838351	0.000124	0.044303
ENSRNOG00000034228	Pik3c2g	34.04206	-1.96952	0.513004	-3.83918	0.000123	0.044303
ENSRNOG00000043410	Cfap300	172.7566	-1.25588	0.325085	-3.86323	0.000112	0.044303
ENSRNOG00000047393	Krt18	80.52144	-2.28045	0.59312	-3.84484	0.000121	0.044303
ENSRNOG00000047393	Krt18	80.52144	-2.28045	0.59312	-3.84484	0.000121	0.044303
ENSRNOG00000054157	Nadk2	6214.314	0.548425	0.143574	3.819792	0.000134	0.046445
ENSRNOG00000011503	Shb	315.9821	-0.78997	0.207439	-3.8082	0.00014	0.047618

Supplementary Table S3. Summary of statistical analyses for all differentially expressed genes in whole brain of stressed zebrafish vs. control groups, on the Negative Binomial (Gamma-Poisson) distribution performed by estimation of size factors, estimation of dispersion and negative binomial generalized linear models and Wald statistics using the DESeq function (Experiment 1) [S3]. P value and false discovery rate were set at 0.05. Genes in this table are listed as sorted by their p-adjusted value.

Lookup	external_gene_name	baseMean	log2FoldChange	lfcSE	stat	pvalue	padj
ENSDARG00000093998	si:ch73-7i4.2	164.8442	5.686818	0.620534	9.164391	4.98E-20	1.21E-15
ENSDARG00000102639	si:dkey-29d5.2	41.03763	6.214117	0.924045	6.724906	1.76E-11	2.14E-07
ENSDARG00000079402	tapbp.1	144.5193	-1.78864	0.284994	-6.27605	3.47E-10	2.82E-06
ENSDARG00000094554	si:ch73-7i4.1	37.6995	4.12461	0.750041	5.499177	3.82E-08	0.000232
ENSDARG00000076586	csf2rb	50.70728	-1.85704	0.341433	-5.43895	5.36E-08	0.000248
ENSDARG00000090890	cmklr1	55.55607	-1.7606	0.325129	-5.41508	6.13E-08	0.000248
ENSDARG00000100302	si:dkey-237g15.2	108.2872	-3.08287	0.601631	-5.12419	2.99E-07	0.000891
ENSDARG00000079034	zpax4	15.31746	2.711004	0.529375	5.121144	3.04E-07	0.000891
ENSDARG00000102835	CABZ01021435.1	26.96331	-1.9449	0.380925	-5.10574	3.3E-07	0.000891
ENSDARG00000069839	CU571382.1	29.63909	-3.33594	0.680015	-4.90569	9.31E-07	0.002138
ENSDARG00000090637	myh6	13.27576	-5.20513	1.062621	-4.89839	9.66E-07	0.002138
ENSDARG00000053973	fetub	6.957216	3.670704	0.752827	4.875896	1.08E-06	0.002197
ENSDARG00000086374	isg15	261.0382	-3.83891	0.793292	-4.83921	1.3E-06	0.002263
ENSDARG00000104120	LO018260.1	67.36825	-1.21762	0.252712	-4.8182	1.45E-06	0.002263
ENSDARG00000002988	tnnt2d	40.15867	2.050141	0.426229	4.809951	1.51E-06	0.002263
ENSDARG00000026979	krt1-c5	299.3362	7.251248	1.510097	4.801841	1.57E-06	0.002263
ENSDARG00000077249	gmip	54.58877	-1.06096	0.221001	-4.80072	1.58E-06	0.002263
ENSDARG00000094719	CR318588.3	1227.035	-4.16004	0.869977	-4.78178	1.74E-06	0.002306
ENSDARG00000078389	ifi46	158.1358	-2.49557	0.522744	-4.77398	1.81E-06	0.002306
ENSDARG00000074136	slc47a2.2	13.28693	-4.82162	1.01405	-4.75481	1.99E-06	0.002306
ENSDARG00000014427	mxe	68.81475	-4.23896	0.891577	-4.75445	1.99E-06	0.002306
ENSDARG00000079997	si:zfos-223e1.2	33.87397	1.598868	0.3406	4.694269	2.68E-06	0.002872
ENSDARG00000059294	marco	174.3955	-2.23722	0.476887	-4.6913	2.71E-06	0.002872
ENSDARG00000087102	si:ch1073-164k15.3	54.19928	-4.9841	1.067979	-4.66685	3.06E-06	0.003101
ENSDARG00000055186	ccr9a	44.12408	-2.80143	0.603577	-4.64138	3.46E-06	0.003369
ENSDARG00000094386	adgrg4b	49.28134	8.419412	1.829425	4.602218	4.18E-06	0.003913
ENSDARG00000088251	lgals17	195.6001	-4.19997	0.922056	-4.55501	5.24E-06	0.004605
ENSDARG00000100732	si:dkeyp-93h6.1	24.14235	1.992828	0.437833	4.551574	5.32E-06	0.004605
ENSDARG00000039490	pitpnaa	65.80556	-1.27998	0.281611	-4.54521	5.49E-06	0.004605
ENSDARG00000029720	si:dkeyp-9d4.2	118.4261	-2.75882	0.609126	-4.52914	5.92E-06	0.004758
ENSDARG00000010946	cbsb	1229.743	-1.11274	0.24621	-4.51949	6.2E-06	0.004758
ENSDARG00000101585	tecra	76.98249	6.34775	1.405135	4.517538	6.26E-06	0.004758
ENSDARG00000067990	myhz1.1	64.67868	2.700507	0.601157	4.492184	7.05E-06	0.005175
ENSDARG00000010317	gpr183a	18.51935	-1.57612	0.351277	-4.48682	7.23E-06	0.005175
ENSDARG00000114894	znf1026	19.7091	-1.38193	0.310862	-4.44548	8.77E-06	0.005937
ENSDARG00000010619	themis	14.19646	-1.81447	0.40819	-4.44516	8.78E-06	0.005937
ENSDARG00000090600	si:ch211-213a13.1	323.8362	-3.22941	0.728271	-4.43435	9.24E-06	0.006074
ENSDARG00000117491	CU855688.1	14.46722	1.922018	0.434193	4.426639	9.57E-06	0.00613
ENSDARG00000092233	vtg1	176.6857	2.944982	0.6664	4.419243	9.9E-06	0.006181

ENSDARG00000028731	stat4	34.94947	-1.58575	0.36035	-4.40058	1.08E-05	0.006568
ENSDARG00000007467	ifit10	18.52404	-4.94187	1.137183	-4.34571	1.39E-05	0.007947
ENSDARG00000069961	il21r.1	63.55553	-1.29313	0.297719	-4.34348	1.4E-05	0.007947
ENSDARG00000032885	si:ch211-226m7.4	33.89089	1.547943	0.356405	4.343211	1.4E-05	0.007947
ENSDARG00000093214	si:ch211-284e13.9	23.50924	-1.66773	0.388949	-4.2878	1.8E-05	0.009981
ENSDARG00000095673	si:dkey-79f11.8	30.39832	-6.4794	1.529671	-4.23581	2.28E-05	0.012115
ENSDARG00000088831	si:dkey-81j8.6	22.07982	-1.99191	0.470394	-4.23455	2.29E-05	0.012115
ENSDARG00000101014	slco1f4	4.985412	5.932084	1.402738	4.228934	2.35E-05	0.012158
ENSDARG00000105266	si:dkey-16p6.1	14.8571	-2.59149	0.615033	-4.21357	2.51E-05	0.012737
ENSDARG00000075346	tmem129	255.4415	0.505023	0.119985	4.209047	2.56E-05	0.012737
ENSDARG00000074283	inpp5d	116.6038	-1.11091	0.264307	-4.2031	2.63E-05	0.012815
ENSDARG00000055809	vtg2	19.39724	5.828072	1.392504	4.185318	2.85E-05	0.013357
ENSDARG00000043475	si:ch211-122h15.4	113.3422	-1.67301	0.400194	-4.1805	2.91E-05	0.013357
ENSDARG00000095026	si:dkey-58f10.14	10.72728	-4.15513	0.994521	-4.17802	2.94E-05	0.013357
ENSDARG00000071543	si:dkey-42i9.7	28.76336	-2.9514	0.706858	-4.17538	2.97E-05	0.013357
ENSDARG00000062049	arhgap45b	99.35305	-0.92879	0.222678	-4.17098	3.03E-05	0.013357
ENSDARG00000101728	si:dkeyp-80d11.12	9.188694	-3.03997	0.729765	-4.16568	3.1E-05	0.013357
ENSDARG00000071437	ptprc	343.6064	-1.11075	0.266757	-4.1639	3.13E-05	0.013357
ENSDARG00000056791	pde6ga	21.88924	-3.64709	0.878431	-4.15182	3.3E-05	0.01384
ENSDARG00000032631	ltb4r	39.66947	-1.54528	0.373787	-4.13413	3.56E-05	0.014438
ENSDARG00000099949	FP236551.1	48.90707	-1.8141	0.439081	-4.13158	3.6E-05	0.014438
ENSDARG00000074052	si:ch211-281g13.5	61.93331	-3.65391	0.884609	-4.13054	3.62E-05	0.014438
ENSDARG00000089463	dhx58	63.537	-3.29948	0.802559	-4.1112	3.94E-05	0.01545
ENSDARG00000041379	si:ch211-245j22.3	53.2759	-5.22288	1.276876	-4.09036	4.31E-05	0.016638
ENSDARG00000111309	AL935126.2	37.11771	7.400761	1.812357	4.083502	4.44E-05	0.016869
ENSDARG00000077090	si:ch211-127b11.1	16.06137	5.489022	1.34639	4.076845	4.57E-05	0.017015
ENSDARG00000052288	zmp:0000000634	80.56334	-5.23374	1.284563	-4.07434	4.61E-05	0.017015
ENSDARG00000052165	si:ch211-236k19.2	75.20497	5.488341	1.349347	4.067406	4.75E-05	0.017016
ENSDARG00000094668	si:ch211-51a19.5	85.66699	2.73115	0.671709	4.065971	4.78E-05	0.017016
ENSDARG00000054418	ssuh2.2	16.49514	-2.77854	0.685146	-4.0554	5E-05	0.017016
ENSDARG00000102525	lck	17.0159	-1.98235	0.488948	-4.05431	5.03E-05	0.017016
ENSDARG00000004459	unc119.2	45.52201	-4.57894	1.130001	-4.05215	5.07E-05	0.017016
ENSDARG00000058256	draxin	1407.05	0.665238	0.164239	4.050415	5.11E-05	0.017016
ENSDARG00000098315	cyp1a	20.05002	3.458075	0.854267	4.048005	5.17E-05	0.017016
ENSDARG00000019728	bmpr1aa	439.3845	0.627031	0.154914	4.047613	5.17E-05	0.017016
ENSDARG00000074656	ctss2.1	46.2542	-1.63224	0.403898	-4.04123	5.32E-05	0.017253
ENSDARG00000015176	ror1	39.3239	-1.00205	0.248429	-4.03355	5.49E-05	0.017592
ENSDARG00000075389	si:ch211-281g13.4	30.43102	-3.7568	0.933473	-4.02454	5.71E-05	0.018042
ENSDARG00000007018	ms4a17a.6	23.82854	-1.55099	0.386569	-4.0122	6.02E-05	0.018768
ENSDARG00000004953	mxb	68.51614	-3.91733	0.979392	-3.99976	6.34E-05	0.019415
ENSDARG00000035798	gngt1	231.2607	-4.58871	1.148379	-3.99582	6.45E-05	0.019415
ENSDARG00000088366	zgc:77938	193.9505	1.411368	0.35339	3.993799	6.5E-05	0.019415
ENSDARG00000038095	socs1a	60.44625	-1.72096	0.431249	-3.99065	6.59E-05	0.019415
ENSDARG00000076182	stat1b	200.0531	-3.00857	0.754124	-3.98949	6.62E-05	0.019415
ENSDARG00000075757	gig2e	11.43792	-2.30678	0.578929	-3.98457	6.76E-05	0.019586
ENSDARG00000074546	si:ch211-213a13.2	207.1771	-4.26256	1.071408	-3.97847	6.94E-05	0.019801

ENSDARG00000104045	tlr22	9.832313	-2.26098	0.569042	-3.97331	7.09E-05	0.019801
ENSDARG00000037408	tbc1d10c	41.53559	-1.194	0.300553	-3.97268	7.11E-05	0.019801
ENSDARG00000015752	zap70	32.19613	-1.42027	0.357668	-3.9709	7.16E-05	0.019801
ENSDARG00000052396	pkz	35.78193	-2.68548	0.677851	-3.96175	7.44E-05	0.020344
ENSDARG00000010252	jak3	44.89497	-1.21599	0.307974	-3.94835	7.87E-05	0.021278
ENSDARG00000040942	pnp6	782.434	0.691913	0.175374	3.945363	7.97E-05	0.021308
ENSDARG00000075072	rpap1	258.9355	-0.62954	0.160114	-3.93184	8.43E-05	0.022002
ENSDARG00000092259	si:dkey-211g8.5	3.67091	-5.58819	1.422267	-3.92907	8.53E-05	0.022002
ENSDARG00000045822	tnnt2e	97.31019	2.923894	0.744396	3.927875	8.57E-05	0.022002
ENSDARG00000014196	myl1	22.47509	2.590033	0.659487	3.927348	8.59E-05	0.022002
ENSDARG00000101071	si:dkeyp-80d11.14	9.692705	-1.69541	0.43224	-3.92239	8.77E-05	0.022226
ENSDARG00000037358	polr3e	639.91	0.740317	0.189372	3.909328	9.26E-05	0.023042
ENSDARG00000093748	si:ch211-217k17.11	74.89433	-3.07385	0.786411	-3.90871	9.28E-05	0.023042
ENSDARG00000105364	si:ch73-223f5.1	249.9613	-2.25547	0.578322	-3.90002	9.62E-05	0.023602
ENSDARG00000055730	trim35-29	26.2314	2.224615	0.570704	3.89802	9.7E-05	0.023602
ENSDARG00000076158	zgc:165583	14.77398	-1.73106	0.444598	-3.89354	9.88E-05	0.023804
ENSDARG00000075504	si:dkey-40c23.2	39.2451	-1.77002	0.455386	-3.88687	0.000102	0.024005
ENSDARG00000098189	si:dkey-16p6.1	21.01967	-2.0213	0.520049	-3.88674	0.000102	0.024005
ENSDARG00000117508	CU459056.2	31.31827	-2.81318	0.726197	-3.87385	0.000107	0.025048
ENSDARG00000104450	CR855996.2	18.79987	2.993049	0.773054	3.871719	0.000108	0.025048
ENSDARG00000040582	C18H3orf33	96.04662	0.956905	0.247762	3.862198	0.000112	0.025602
ENSDARG00000092191	CR318588.1	1186.549	-3.8714	1.002492	-3.86178	0.000113	0.025602
ENSDARG00000058774	CABZ01053221.1	6.751038	-2.13749	0.554449	-3.85516	0.000116	0.02606
ENSDARG00000077130	bcl10	19.27022	-1.44581	0.375445	-3.85093	0.000118	0.026272
ENSDARG00000025428	socs3a	319.8547	-2.28392	0.593862	-3.84587	0.00012	0.026576
ENSDARG00000022183	gsto1	561.16	0.791405	0.206245	3.837212	0.000124	0.027056
ENSDARG00000061177	mov10b.1	67.60348	-2.23373	0.582393	-3.83543	0.000125	0.027056
ENSDARG00000044235	BX005421.1	11.77669	-1.87999	0.490235	-3.83487	0.000126	0.027056
ENSDARG00000070028	tmem41aa	294.3783	-0.52064	0.136132	-3.82454	0.000131	0.027969
ENSDARG00000086391	cald1b	29.47612	1.741375	0.455576	3.822362	0.000132	0.027971
ENSDARG00000013855	slc12a3	25.84152	2.833511	0.741834	3.819602	0.000134	0.028042
ENSDARG00000100968	si:ch211-1a19.3	127.6878	-1.3544	0.355749	-3.80717	0.000141	0.029237
ENSDARG00000097559	cyp8b3	23.46184	-3.1911	0.83928	-3.80219	0.000143	0.02958
ENSDARG00000093753	BX004774.2	193.7659	-3.35418	0.884297	-3.79305	0.000149	0.030432
ENSDARG00000099860	pkmb	19.15483	1.985092	0.52447	3.784949	0.000154	0.031102
ENSDARG00000013655	tpd52l2b	1755.256	0.396047	0.104678	3.783498	0.000155	0.031102
ENSDARG00000032584	thnsl2	58.5869	1.312257	0.347211	3.77942	0.000157	0.031356
ENSDARG00000112454	FO744833.2	250.7418	-1.06081	0.281449	-3.7691	0.000164	0.032415
ENSDARG00000107526	smkr1	91.23716	1.22454	0.325166	3.765891	0.000166	0.032449
ENSDARG00000039243	zgc:152791	379.5849	-4.08968	1.08629	-3.76482	0.000167	0.032449
ENSDARG00000038288	cd151	53.99511	1.22684	0.326066	3.762555	0.000168	0.032484
ENSDARG00000101169	grap2b	69.45649	-1.34499	0.357704	-3.76006	0.00017	0.032551
ENSDARG00000068939	xaf1	80.99448	-2.10235	0.5609	-3.74818	0.000178	0.033866
ENSDARG00000043483	otx5	21.80288	-4.75563	1.270297	-3.74372	0.000181	0.033915
ENSDARG00000078731	si:dkey-85k7.12	46.23873	-3.24038	0.865637	-3.74335	0.000182	0.033915
ENSDARG00000092653	si:dkey-1h24.6	10.56826	-1.46668	0.391992	-3.7416	0.000183	0.033915

ENSDARG00000100900	si:ch211-183d5.2	90.99566	-2.64398	0.70701	-3.73967	0.000184	0.033915
ENSDARG00000002682	tbcela	1466.567	0.437677	0.117083	3.738187	0.000185	0.033915
ENSDARG00000092126	vtg5	23.70013	3.134181	0.842453	3.720305	0.000199	0.036138
ENSDARG00000101272	si:dkey-29j8.1	6.759197	-1.94503	0.523401	-3.71615	0.000202	0.036465
ENSDARG00000055159	cyp27a1.4	191.0922	0.818813	0.220705	3.709983	0.000207	0.036863
ENSDARG00000097580	BX663519.1	55.41507	2.045346	0.55146	3.708969	0.000208	0.036863
ENSDARG00000053323	zgc:112285	19.59111	1.085756	0.292827	3.707837	0.000209	0.036863
ENSDARG00000089185	si:ch73-264i18.2	2.574693	5.23719	1.41348	3.705175	0.000211	0.036985
ENSDARG00000032932	cnksr1	19.13048	-1.15229	0.311167	-3.70313	0.000213	0.037018
ENSDARG00000034063	unm_sa911	124.8442	-1.01276	0.273941	-3.697	0.000218	0.037478
ENSDARG00000054610	coro1a	158.7542	-1.13353	0.306657	-3.69639	0.000219	0.037478
ENSDARG00000004954	grna	435.3453	-0.97673	0.264828	-3.68817	0.000226	0.038276
ENSDARG00000089227	si:ch211-91p5.3	148.7863	-1.22865	0.333224	-3.68714	0.000227	0.038276
ENSDARG00000090730	cfbl	23.55705	-1.94248	0.527028	-3.68572	0.000228	0.038276
ENSDARG00000008592	mtmr8	211.84	-1.80008	0.489257	-3.67922	0.000234	0.038995
ENSDARG00000074501	tnip2	14.95865	-1.8318	0.498515	-3.67452	0.000238	0.039451
ENSDARG00000094451	cfp	291.775	-1.13416	0.309322	-3.66658	0.000246	0.040309
ENSDARG00000087641	cdh26.2	27.49433	2.062629	0.563345	3.661399	0.000251	0.040309
ENSDARG00000071558	fblim1	86.34791	0.791259	0.216127	3.661085	0.000251	0.040309
ENSDARG00000002165	psme1	196.5018	-1.24669	0.340793	-3.6582	0.000254	0.040309
ENSDARG00000093254	znf569l	22.27115	-1.74352	0.476823	-3.65653	0.000256	0.040309
ENSDARG00000112831	znf1116	5.616213	-3.68212	1.007437	-3.65494	0.000257	0.040309
ENSDARG00000096045	si:ch73-263o4.4	21.07568	1.864121	0.510054	3.654755	0.000257	0.040309
ENSDARG00000045592	tnni2a.1	41.53237	4.136382	1.131927	3.654282	0.000258	0.040309
ENSDARG00000113386	LO018432.1	13.70658	-3.40887	0.933373	-3.6522	0.00026	0.040309
ENSDARG00000094854	ms4a17a.9	23.93016	-1.12734	0.308705	-3.65183	0.00026	0.040309
ENSDARG00000034095	si:dkey-59l11.10	4.273366	-3.45926	0.947606	-3.65053	0.000262	0.040309
ENSDARG00000092159	CR788249.1	20.92327	-4.21296	1.157248	-3.6405	0.000272	0.041537
ENSDARG00000039512	ap1m3	51.50422	-1.22567	0.336763	-3.63957	0.000273	0.041537
ENSDARG00000096355	ighv1-4	116.5287	-4.07637	1.120754	-3.63717	0.000276	0.041666
ENSDARG00000100434	znf1091	28.42767	-3.03531	0.83643	-3.62889	0.000285	0.04275
ENSDARG00000008306	rdh5	41.4861	-3.83153	1.056286	-3.62736	0.000286	0.04275
ENSDARG00000094901	abcc6b.2	17.71361	1.093126	0.301822	3.621754	0.000293	0.043175
ENSDARG00000114991	tmem14cb	32.63878	-1.22506	0.338416	-3.61999	0.000295	0.043175
ENSDARG00000015482	zgc:113518	197.057	0.514276	0.142076	3.619716	0.000295	0.043175
ENSDARG00000095024	si:dkeyp-68b7.10	5.475087	2.323612	0.642833	3.614645	0.000301	0.043175
ENSDARG00000069774	flot2b	1183.238	0.363745	0.100632	3.614625	0.000301	0.043175
ENSDARG00000075599	cdc25d	51.14493	0.687367	0.190245	3.613054	0.000303	0.043175
ENSDARG00000100669	si:dkey-61p9.9	18.42742	-1.47296	0.408158	-3.60879	0.000308	0.043175
ENSDARG00000019128	tpm4b	69.61131	1.403176	0.38894	3.607694	0.000309	0.043175
ENSDARG00000002696	gnb3b	50.1038	-2.72007	0.754059	-3.60724	0.000309	0.043175
ENSDARG00000004952	rsad2	151.1438	-2.29699	0.636882	-3.60662	0.00031	0.043175
ENSDARG00000098515	si:dkey-16p6.1	54.17549	-1.69489	0.469968	-3.60639	0.00031	0.043175
ENSDARG00000100568	si:ch211-209j12.3	5.64605	-3.59469	0.996871	-3.60598	0.000311	0.043175
ENSDARG00000052223	rcvrna	24.89004	-4.98773	1.38359	-3.60492	0.000312	0.043175
ENSDARG00000044612	c1qb	41.35639	-1.48744	0.412887	-3.60255	0.000315	0.043325

ENSDARG00000101309	si:ch73-100l22.3	227.2183	0.752427	0.209258	3.595699	0.000324	0.044232
ENSDARG00000020610	tnnt2a	13.70478	2.009186	0.559411	3.59161	0.000329	0.044433
ENSDARG00000101675	zgc:123107	60.63829	-1.22681	0.341577	-3.5916	0.000329	0.044433
ENSDARG00000098811	LO018021.1	38.82198	0.879947	0.245746	3.580713	0.000343	0.046016
ENSDARG00000098046	FP017161.1	196.3582	0.871499	0.243536	3.578517	0.000346	0.046016
ENSDARG00000005713	ethe1	180.6208	0.715788	0.200044	3.578154	0.000346	0.046016
ENSDARG00000054340	sla1a	12.8555	-1.84624	0.517082	-3.5705	0.000356	0.047124
ENSDARG00000078618	inpp5kb	54.3152	-2.24734	0.629697	-3.56892	0.000358	0.047153
ENSDARG00000089930	iqce	451.2363	0.846778	0.237392	3.567004	0.000361	0.047244
ENSDARG00000103551	rnf213b	78.05034	-2.76741	0.777137	-3.56103	0.000369	0.048014
ENSDARG00000044199	gnat1	271.0448	-4.22288	1.186217	-3.55995	0.000371	0.048014
ENSDARG00000078130	CR391986.1	68.27406	0.555449	0.156091	3.558495	0.000373	0.048026
ENSDARG00000091627	si:dkey-271j15.3	18.75689	-2.85533	0.802833	-3.55657	0.000376	0.048046
ENSDARG00000100420	tpcn2	38.46233	-2.2763	0.640198	-3.55562	0.000377	0.048046
ENSDARG00000103720	zgc:162730	428.5616	-1.05731	0.297686	-3.55178	0.000383	0.048499
ENSDARG00000038941	ppp1r1c	914.9612	0.947675	0.26721	3.546548	0.00039	0.049026
ENSDARG00000034160	slc4a1ap	852.5459	0.356273	0.100466	3.546204	0.000391	0.049026
ENSDARG00000090977	ifit12	8.278224	-2.10441	0.593758	-3.54422	0.000394	0.049144
ENSDARG00000086848	atad3	94.54343	-0.72335	0.204181	-3.5427	0.000396	0.049174

Supplementary Table S4. Statistical data for pathways enriched in subiculum of mdd vs. control groups (Experiment 1). The KEGG- enrichment analysis was performed on normalized and log2-transformed counts by general applicable gene set enrichment for pathway analysis (GAGE) package [S4], using two-tailed t-test for group comparison of differential expression of gene sets. False discovery rate cutoff (q.val) was set at 0.05. p.geomean – geometric mean of the individual p-values from multiple single array based gene set tests, stat.mean - mean of the individual statistics from multiple single array based gene set tests. Pathways are listed as sorted by q value.

**Pathway code and name**	**p.geomean**	**stat.mean**	**p.val**	**q.val**	**set.size**
Down					
hsa05010 Alzheimer's disease	3.49E-06	-4.11165	9.03E-13	2.75E-10	354
hsa05012 Parkinson's disease	3.84E-06	-4.01005	4.17E-12	6.34E-10	239
hsa05014 Amyotrophic lateral sclerosis (ALS)	2.34E-05	-3.81672	2.77E-11	2.71E-09	352
hsa05016 Huntington's disease	1.53E-05	-3.80574	3.57E-11	2.71E-09	296
hsa05020 Prion diseases	1.33E-05	-3.72127	1.02E-10	6.22E-09	258
hsa00190 Oxidative phosphorylation	0.000249	-2.85921	6.67E-07	3.38E-05	129
hsa04141 Protein processing in endoplasmic reticulum	0.002934	-2.54964	6.37E-06	0.000277	162
hsa04932 Non-alcoholic fatty liver disease (NAFLD)	0.00234	-2.50714	9.08E-06	0.00031	144
hsa04723 Retrograde endocannabinoid signaling	0.00216	-2.50317	9.18E-06	0.00031	147
hsa04144 Endocytosis	0.007204	-2.42309	1.45E-05	0.000442	247
hsa03050 Proteasome	0.013337	-2.20299	9.94E-05	0.002748	43
hsa05132 Salmonella infection	0.014806	-1.97907	0.000322	0.008158	242
hsa05203 Viral carcinogenesis	0.018831	-1.91241	0.00049	0.011113	191
hsa04720 Long-term potentiation	0.024275	-1.91014	0.000542	0.011113	66
hsa04120 Ubiquitin mediated proteolysis	0.017009	-1.90206	0.000548	0.011113	140
hsa04961 Endocrine and other factor-regulated calcium reabsorption	0.03347	-1.79956	0.001047	0.019895	50
hsa04070 Phosphatidylinositol signaling system	0.036375	-1.75836	0.001247	0.021288	96
hsa03040 Spliceosome	0.027918	-1.75562	0.00126	0.021288	140
hsa03013 RNA transport	0.033042	-1.70589	0.001635	0.026161	176
hsa04140 Autophagy - animal	0.031896	-1.67827	0.001943	0.029152	135
hsa04730 Long-term depression	0.042124	-1.67146	0.002097	0.029152	57
hsa04921 Oxytocin signaling pathway	0.038339	-1.65288	0.002189	0.029152	151
hsa05032 Morphine addiction	0.045436	-1.65123	0.002239	0.029152	90
hsa04728 Dopaminergic synapse	0.048628	-1.64251	0.002302	0.029152	127
hsa04150 mTOR signaling pathway	0.044761	-1.61578	0.002661	0.031557	150
hsa04725 Cholinergic synapse	0.030497	-1.6201	0.002699	0.031557	113
hsa04145 Phagosome	0.048536	-1.58089	0.003217	0.035162	133
hsa03020 RNA polymerase	0.04288	-1.62021	0.003239	0.035162	31
hsa04721 Synaptic vesicle cycle	0.056264	-1.5736	0.003387	0.035502	76
hsa04137 Mitophagy - animal	0.052555	-1.51325	0.004776	0.048395	66
hsa04724 Glutamatergic synapse	0.061127	-1.49279	0.00503	0.049327	112
Bidirected					
hsa04080 Neuroactive ligand-receptor interaction	5.47E-07	4.815058	8.46E-17	2.57E-14	316
hsa05150 Staphylococcus aureus infection	0.004399	2.638553	4.39E-06	0.000533	52
hsa05332 Graft-versus-host disease	0.005939	2.688021	7.12E-06	0.000533	23
hsa04640 Hematopoietic cell lineage	0.005856	2.5234	8.34E-06	0.000533	81
hsa04610 Complement and coagulation cascades	0.005084	2.510456	9.25E-06	0.000533	76

hsa05323 Rheumatoid arthritis	0.007059	2.479163	1.18E-05	0.000533	73
hsa04514 Cell adhesion molecules (CAMs)	0.007239	2.456054	1.23E-05	0.000533	135
hsa05330 Allograft rejection	0.007763	2.562593	1.4E-05	0.000534	25
hsa05144 Malaria	0.009431	2.357339	3.17E-05	0.000991	49
hsa04060 Cytokine-cytokine receptor interaction	0.010933	2.29777	3.64E-05	0.000991	255
hsa04940 Type I diabetes mellitus	0.010599	2.392429	3.9E-05	0.000991	29
hsa04020 Calcium signaling pathway	0.004718	2.296864	3.91E-05	0.000991	233
hsa04974 Protein digestion and absorption	0.011532	2.284009	4.39E-05	0.001027	99
hsa04672 Intestinal immune network for IgA production	0.012509	2.285432	5.88E-05	0.001277	38
hsa04512 ECM-receptor interaction	0.013643	2.217787	7.11E-05	0.001441	85
hsa04742 Taste transduction	0.017317	2.105414	0.000157	0.002976	67
hsa05140 Leishmaniasis	0.020341	2.038571	0.000246	0.004234	65
hsa05320 Autoimmune thyroid disease	0.021985	2.058808	0.000251	0.004234	41
hsa05322 Systemic lupus erythematosus	0.02584	1.934068	0.000438	0.007005	109
hsa05033 Nicotine addiction	0.029429	1.827891	0.000957	0.014083	40
hsa04145 Phagosome	0.035331	1.798504	0.000973	0.014083	133
hsa05143 African trypanosomiasis	0.03454	1.772496	0.001354	0.018706	34
hsa04950 Maturity onset diabetes of the young	0.04713	1.689918	0.002263	0.029905	20
hsa04612 Antigen processing and presentation	0.049501	1.63078	0.002755	0.034902	48
hsa05416 Viral myocarditis	0.054312	1.59989	0.00319	0.03879	48

Supplementary Table S5. Statistical data for pathways enriched in hippocampus of stressed rats vs. control groups (Experiment 1). The KEGG- enrichment analysis was performed on normalized and log2-transformed counts by general applicable gene set enrichment for pathway analysis (GAGE) package [S4], using two-tailed t-test for group comparison of differential expression of gene sets. False discovery rate cutoff (q.val) was set at 0.05. p.geomean – geometric mean of the individual p-values from multiple single array based gene set tests, stat.mean - mean of the individual statistics from multiple single array based gene set tests. Pathways are listed as sorted by q value.

**Pathway code and name**	**p.geomean**	**stat.mean**	**p.val**	**q.val**	**set.size**
Bidirected					
rno04080 Neuroactive ligand-receptor interaction	5.28E-10	6.156581	7.78E-26	2.35E-23	308
rno04020 Calcium signaling pathway	1.25E-05	4.202168	3.23E-13	4.88E-11	228
rno04024 cAMP signaling pathway	0.001263	3.029862	9.48E-08	9.54E-06	204
rno04512 ECM-receptor interaction	0.001888	2.937281	2.72E-07	2.06E-05	83
rno04974 Protein digestion and absorption	0.003102	2.667433	2.68E-06	0.000162	88
rno04725 Cholinergic synapse	0.006011	2.517783	7.74E-06	0.00039	111
rno05205 Proteoglycans in cancer	0.007612	2.421101	1.5E-05	0.000647	196
rno05033 Nicotine addiction	0.007803	2.446016	1.88E-05	0.00071	40
rno04916 Melanogenesis	0.008593	2.376201	2.34E-05	0.000786	94
rno04514 Cell adhesion molecules (CAMs)	0.010273	2.284123	4.24E-05	0.001279	152
rno04360 Axon guidance	0.011027	2.25213	5.22E-05	0.001433	177
rno05217 Basal cell carcinoma	0.01534	2.175383	0.000103	0.002589	60
rno05144 Malaria	0.015083	2.163642	0.000116	0.002589	49
rno05032 Morphine addiction	0.012979	2.142934	0.00012	0.002589	91
rno04510 Focal adhesion	0.017356	2.07428	0.000173	0.003479	195
rno05332 Graft-versus-host disease	0.019409	2.084472	0.000198	0.00355	45
rno04151 PI3K-Akt signaling pathway	0.019848	2.043605	0.000206	0.00355	318
rno04713 Circadian entrainment	0.019103	2.05153	0.000212	0.00355	94
rno04550 Signaling pathways regulating pluripotency of stem cells	0.01962	2.031009	0.000236	0.003751	132
rno04911 Insulin secretion	0.022237	2.022297	0.000257	0.003877	79
rno04390 Hippo signaling pathway	0.021422	2.002419	0.000279	0.004011	152
rno05414 Dilated cardiomyopathy	0.023994	1.986874	0.000318	0.00437	92
rno04310 Wnt signaling pathway	0.025012	1.963281	0.000354	0.004654	156
rno04060 Cytokine-cytokine receptor interaction	0.025925	1.933765	0.000418	0.005081	232
rno05320 Autoimmune thyroid disease	0.02198	1.968404	0.000421	0.005081	51
rno04022 cGMP-PKG signaling pathway	0.026785	1.908573	0.000497	0.005768	161
rno04925 Aldosterone synthesis and secretion	0.028509	1.889699	0.000577	0.006458	91
rno05330 Allograft rejection	0.027437	1.89411	0.000639	0.006896	45
rno04726 Serotonergic synapse	0.031746	1.860567	0.000674	0.007018	109
rno04727 GABAergic synapse	0.031528	1.838824	0.000781	0.00785	90
rno04940 Type I diabetes mellitus	0.032171	1.846487	0.000806	0.00785	52
rno05416 Viral myocarditis	0.031327	1.831201	0.000845	0.007976	74
rno05030 Cocaine addiction	0.03501	1.810879	0.00102	0.009338	47
rno05224 Breast cancer	0.041262	1.739239	0.001347	0.011962	140
rno04724 Glutamatergic synapse	0.037539	1.726134	0.001475	0.012726	111

rno05031 Amphetamine addiction	0.040831	1.72049	0.001584	0.013286	66
rno04261 Adrenergic signaling in cardiomyocytes	0.04121	1.678254	0.0019	0.01551	144
rno04970 Salivary secretion	0.042116	1.676596	0.001989	0.015808	73
rno05150 Staphylococcus aureus infection	0.048764	1.665001	0.002084	0.016138	79
rno05410 Hypertrophic cardiomyopathy (HCM)	0.048405	1.652762	0.002221	0.016771	89
rno04270 Vascular smooth muscle contraction	0.047929	1.643108	0.002303	0.016962	128
rno04742 Taste transduction	0.051158	1.615624	0.002779	0.019985	59
rno04921 Oxytocin signaling pathway	0.056456	1.577427	0.003232	0.022696	149
rno04640 Hematopoietic cell lineage	0.056987	1.567807	0.003495	0.023981	80
rno04971 Gastric acid secretion	0.059906	1.562787	0.003573	0.023981	73
rno04750 Inflammatory mediator regulation of TRP channels	0.062593	1.539136	0.003975	0.025972	100
rno04371 Apelin signaling pathway	0.057528	1.534992	0.004042	0.025972	133
rno05143 African trypanosomiasis	0.055835	1.53999	0.004303	0.027074	37
rno04728 Dopaminergic synapse	0.050506	1.508809	0.004677	0.028825	129
rno00830 Retinol metabolism	0.062863	1.508464	0.00478	0.028872	64
rno04918 Thyroid hormone synthesis	0.066444	1.481413	0.00547	0.03239	71
rno04610 Complement and coagulation cascades	0.065628	1.461396	0.006011	0.034908	77
rno04015 Rap1 signaling pathway	0.075229	1.431529	0.006671	0.038014	204
rno04978 Mineral absorption	0.074575	1.429843	0.007033	0.039332	51
rno04014 Ras signaling pathway	0.080603	1.399161	0.007778	0.042707	218
rno05412 Arrhythmogenic right ventricular cardiomyopathy (ARVC)	0.085488	1.371483	0.009061	0.048863	75
Upregulated					
rno03010 Ribosome	3.37E-06	3.863552	4.09E-11	1.24E-08	177
rno05140 Leishmaniasis	0.003861	2.615486	4.37E-06	0.00066	67
rno05145 Toxoplasmosis	0.008595	2.271695	4.98E-05	0.004193	103
rno05152 Tuberculosis	0.010626	2.244867	5.55E-05	0.004193	155
rno04380 Osteoclast differentiation	0.01247	2.153308	0.000108	0.006521	118
rno05323 Rheumatoid arthritis	0.017972	2.08107	0.000178	0.008984	78
rno00190 Oxidative phosphorylation	0.001352	2.04485	0.000354	0.015273	121
rno04217 Necroptosis	0.026044	1.866219	0.000654	0.023023	138
rno04621 NOD-like receptor signaling pathway	0.028717	1.855761	0.000686	0.023023	156
rno04066 HIF-1 signaling pathway	0.033375	1.778905	0.001104	0.033335	102
rno05310 Asthma	0.032838	1.781298	0.001521	0.041757	20
rno05020 Prion diseases	0.007176	1.699032	0.001784	0.042494	255
rno04145 Phagosome	0.046467	1.683126	0.001829	0.042494	161

Supplementary Table S6. Statistical data for pathways enriched in whole brain of stressed zebrafish vs. control groups (Experiment 1). The KEGG- enrichment analysis was performed on normalized and log2-transformed counts by general applicable gene set enrichment for pathway analysis (GAGE) package [S4], using two-tailed t-test for group comparison of differential expression of gene sets. False discovery rate cutoff (q.val) was set at 0.05. p.geomean – geometric mean of the individual p-values from multiple single array based gene set tests, stat.mean - mean of the individual statistics from multiple single array based gene set tests. Pathways are listed as sorted by q value.

	**p.geomean**	**stat.mean**	**p.val**	**q.val**	**set.size**
Bidirected					
dre04020 Calcium signaling pathway	0.004314	2.588289	1.32E-10	1.99E-08	330
dre04261 Adrenergic signaling in cardiomyocytes	0.005299	2.443404	1.35E-09	1.02E-07	220
dre04010 MAPK signaling pathway	0.003181	2.395348	2.57E-09	1.29E-07	381
dre04512 ECM-receptor interaction	0.006457	2.386706	3.79E-09	1.43E-07	97
dre04510 Focal adhesion	0.021531	1.956686	8.82E-07	2.66E-05	257
dre04912 GnRH signaling pathway	0.018279	1.90655	1.82E-06	4.57E-05	121
dre04920 Adipocytokine signaling pathway	0.028215	1.759246	9.83E-06	0.000203	89
dre04260 Cardiac muscle contraction	0.021	1.755792	1.07E-05	0.000203	134
dre04514 Cell adhesion molecules (CAMs)	0.047469	1.635956	3.28E-05	0.00055	148
dre00230 Purine metabolism	0.05792	1.505363	0.000119	0.001804	158
dre04620 Toll-like receptor signaling pathway	0.06692	1.478278	0.000157	0.002158	99
dre04371 Apelin signaling pathway	0.062956	1.45027	0.0002	0.002514	176
dre04060 Cytokine-cytokine receptor interaction	0.05287	1.178655	0.002056	0.02388	193
dre04080 Neuroactive ligand-receptor interaction	0.106575	1.162578	0.00222	0.023941	468
Upregulated					
dre00190 Oxidative phosphorylation	2.42E-06	2.494845	2.63E-09	3.96E-07	140
dre01200 Carbon metabolism	0.010699	1.814885	5.59E-06	0.000422	136
dre00010 Glycolysis / Gluconeogenesis	0.022143	1.714605	1.72E-05	0.000864	80
dre03040 Spliceosome	0.001216	1.602476	5.5E-05	0.002075	140
dre04260 Cardiac muscle contraction	0.025117	1.466424	0.000203	0.006132	134
dre00071 Fatty acid degradation	0.044922	1.334714	0.000662	0.016652	52
dre00480 Glutathione metabolism	0.0483	1.190117	0.001936	0.041758	63
dre01212 Fatty acid metabolism	0.075775	1.1347	0.002967	0.047066	67
dre03410 Base excision repair	0.046291	1.161291	0.00311	0.047066	37
dre00983 Drug metabolism - other enzymes	0.068099	1.121606	0.003176	0.047066	83
dre00240 Pyrimidine metabolism	0.098205	1.101179	0.003751	0.047066	67
dre03460 Fanconi anemia pathway	0.097773	1.098324	0.004021	0.047066	49
dre00062 Fatty acid elongation	0.114173	1.096112	0.004052	0.047066	35
Downregulated					
dre04010 MAPK signaling pathway	0.000125	-2.07905	2.17E-07	3.28E-05	381
dre04620 Toll-like receptor signaling pathway	0.01601	-1.86209	3.23E-06	0.000225	99
dre04621 NOD-like receptor signaling pathway	0.017636	-1.82603	4.48E-06	0.000225	159
dre04060 Cytokine-cytokine receptor interaction	0.081285	-1.31752	0.000643	0.019971	193
dre04622 RIG-I-like receptor signaling pathway	0.062432	-1.32613	0.000661	0.019971	62

Supplementary Table S7. Summary of over- and under-represented DNA binding sites for transcription factors (TFs) in genes with high variability (p values <0.01) vs. low variability (p>0.7) found in subiculum of mdd vs. control groups using CiiiDer TFMs software [S4] (Experiment 1). Only TFs with p<0.05 simultaneously for gene coverage p-value and the number of the binding sites distributions were considered significant. TFs in the table are listed as sorted by their gene coverage p-value.

TF ID	TF Name	Deficit	N sites	N background sites	Direction	MW U	P-Value	N TF (of 1964)	N TF background genes (of 3948)	Direction	P-Value	Average Log2 Proportion Bound	Log2 Enrichment	Significance Score
MA1484.1	ETS2	0.12	1221	3052	Down	4243002	3.18E-09	817	1990	Down	1.65E-10	-1.12652	-0.2767	-9.78191
MA0475.2	FLI1	0.15	1956	4629	Down	4216407	3.97E-08	1083	2505	Down	8.40E-10	-0.75733	-0.20226	-9.07562
MA0079.4	SP1	0.1	777	2096	Down	4192084	3.42E-07	525	1362	Down	1.23E-09	-1.71872	-0.36735	-8.90979
MA0098.3	ETS1	0.1	1031	2583	Down	4204149	1.20E-07	710	1751	Down	1.68E-09	-1.31998	-0.29454	-8.77588
MA0474.2	ERG	0.11	1453	3564	Down	4221947	2.38E-08	908	2143	Down	5.50E-09	-0.99697	-0.23127	-8.25939
MA0156.2	FEV	0.08	741	1862	Down	4155534	6.57E-06	536	1368	Down	9.68E-09	-1.70061	-0.3438	-8.01409
MA1483.1	ELF2	0.15	884	2239	Down	4182607	7.61E-07	636	1578	Down	1.34E-08	-1.47434	-0.30318	-7.87351
MA0766.2	GATA5	0.07	4550	8250	Up	4150660	9.50E-06	1685	3154	Up	2.16E-08	-0.27244	0.102908	7.666159
MA0750.2	ZBTB7A	0.05	618	1622	Down	4151711	8.78E-06	482	1243	Down	2.65E-08	-1.84623	-0.35866	-7.57608
MA0036.3	GATA2	0.14	4736	8596	Up	4154144	7.30E-06	1703	3198	Up	2.89E-08	-0.25478	0.098249	7.53967
MA0037.3	GATA3	0.05	4743	8610	Up	4153010	7.96E-06	1703	3200	Up	3.50E-08	-0.25433	0.097347	7.45651
MA0076.2	ELK4	0.07	671	1727	Down	4154771	6.96E-06	512	1305	Down	3.58E-08	-1.76762	-0.34184	-7.44571
MA0740.1	KLF14	0.1	644	1711	Down	4157815	5.52E-06	505	1289	Down	3.82E-08	-1.78644	-0.34389	-7.41805
MA0685.1	SP4	0.09	382	1107	Down	4109319	1.70E-04	294	821	Down	5.20E-08	-2.5014	-0.47285	-7.28396
MA0913.2	HOXD9	0.14	4546	8044	Up	4253324	1.13E-09	1646	3079	Up	1.07E-07	-0.30668	0.103849	6.970905
MA0759.1	ELK3	0.07	575	1433	Down	4113353	1.31E-04	427	1108	Down	1.42E-07	-2.01643	-0.36747	-6.84805
MA0146.2	Zfx	0.03	1645	4091	Down	4241283	3.76E-09	1000	2296	Down	1.47E-07	-0.87765	-0.19157	-6.83296
MA1516.1	KLF3	0.06	1672	4038	Down	4204631	1.15E-07	936	2169	Down	1.47E-07	-0.96638	-0.20487	-6.83212
MA1513.1	KLF15	0.14	7057	15975	Down	4179187	1.01E-06	1543	3323	Down	1.49E-07	-0.29828	-0.09936	-6.82558
MA0815.1	TFAP2C(var.3)	0.1	1447	3428	Down	4154073	7.34E-06	747	1783	Down	1.79E-07	-1.27031	-0.24742	-6.74799
MA0028.2	ELK1	0.11	738	1834	Down	4135334	2.91E-05	543	1355	Down	1.92E-07	-1.69814	-0.31133	-6.7159
MA0701.2	LHX9	0.11	5509	9707	Up	4211245	6.36E-08	1587	2955	Up	2.70E-07	-0.36265	0.110497	6.56802
MA0810.1	TFAP2A(var.2)	0.11	6206	13931	Down	4150625	9.52E-06	1603	3425	Down	3.04E-07	-0.24896	-0.08795	-6.51718

MA0746.2	SP3	0.06	427	1197	Down	4108453	1.80E-04	361	950	Down	6.04E-07	-2.24832	-0.38755	-6.21928
MA0710.1	NOTO	0.08	4905	8592	Up	4196869	2.27E-07	1508	2792	Up	6.94E-07	-0.4404	0.1187	6.158608
MA0516.2	SP2	0.12	380	1064	Down	4090788	5.41E-04	311	837	Down	7.00E-07	-2.447	-0.41971	-6.15463
MA1564.1	SP9	0.11	7869	17660	Down	4164416	3.31E-06	1649	3499	Down	7.44E-07	-0.21314	-0.07798	-6.12865
MA1122.1	TFDP1	0.11	3492	7975	Down	4200933	1.59E-07	1401	3050	Down	8.14E-07	-0.42972	-0.11493	-6.08952
MA0811.1	TFAP2B	0.14	12237	27022	Down	4174764	1.45E-06	1825	3788	Down	1.07E-06	-0.08277	-0.04619	-5.96914
MA1481.1	DRGX	0.03	4957	8756	Up	4180668	8.93E-07	1516	2813	Up	1.13E-06	-0.43118	0.115522	5.948621
MA0742.1	Klf12	0.06	258	776	Down	4063520	0.00254	232	652	Down	1.51E-06	-2.83806	-0.4816	-5.82011
MA0524.2	TFAP2C	0.1	4272	9492	Down	4123498	6.64E-05	1383	3009	Down	1.82E-06	-0.44881	-0.11406	-5.73923
MA0877.2	BARHL1	0.09	4106	7666	Up	4081660	9.26E-04	1671	3161	Up	2.33E-06	-0.27686	0.087677	5.633376
MA1517.1	KLF6	0.08	1886	4467	Down	4188036	4.83E-07	1136	2533	Down	2.65E-06	-0.71487	-0.14939	-5.57625
MA0040.1	Foxq1	0.11	1693	2933	Up	4134138	3.17E-05	1044	1843	Up	3.01E-06	-1.0051	0.187508	5.52073
MA0763.1	ETV3	0.15	1220	3026	Down	4181595	8.27E-07	832	1926	Down	3.30E-06	-1.13698	-0.20332	-5.48132
MA0864.2	E2F2	0.15	8061	18239	Down	4202044	1.44E-07	1546	3305	Down	3.36E-06	-0.3008	-0.08872	-5.47305
MA0708.1	MSX2	0.08	3962	7039	Up	4133090	3.41E-05	1382	2543	Up	5.07E-06	-0.57069	0.127607	5.295404
MA1519.1	LHX5	0.08	4711	8555	Up	4122473	7.12E-05	1522	2842	Up	5.20E-06	-0.42093	0.106424	5.28388
MA1515.1	KLF2	0.03	3471	7830	Down	4131387	3.85E-05	1351	2939	Down	5.33E-06	-0.48267	-0.11387	-5.27298
MA0621.1	mix-a	0.15	4275	7630	Up	4126948	5.24E-05	1412	2609	Up	5.90E-06	-0.53672	0.12162	5.229317
MA0748.2	YY2	0.09	1257	3039	Down	4171971	1.81E-06	883	2022	Down	5.90E-06	-1.05901	-0.1877	-5.22881
MA0878.2	CDX1	0.12	1742	3023	Up	4154103	7.32E-06	1092	1951	Up	7.60E-06	-0.93163	0.170192	5.118975
MA0914.1	ISL2	0.11	6623	12190	Up	4163840	3.46E-06	1809	3491	Up	8.64E-06	-0.14801	0.058887	5.06339
MA0813.1	TFAP2B(var.3)	0.11	2062	4812	Down	4153896	7.44E-06	979	2210	Down	9.28E-06	-0.92049	-0.16711	-5.03266
MA0872.1	TFAP2A(var.3)	0.1	1588	3702	Down	4131842	3.73E-05	826	1902	Down	9.30E-06	-1.15124	-0.19567	-5.03147
MA0602.1	Arid5a	0.15	9060	16737	Up	4155949	6.36E-06	1605	3028	Up	9.41E-06	-0.33692	0.091562	5.026306
MA0075.3	PRRX2	0.08	6720	12133	Up	4178714	1.05E-06	1687	3211	Up	1.08E-05	-0.25867	0.078783	4.966863
MA1473.1	CDX4	0.11	2162	3829	Up	4140984	1.94E-05	1219	2214	Up	1.11E-05	-0.7611	0.146451	4.953407
MA1501.1	HOXB7	0.15	3618	6838	Up	4081747	9.22E-04	1510	2825	Up	1.22E-05	-0.43097	0.103663	4.912203
MA0635.1	BARHL2	0.05	2965	5389	Up	4112041	1.43E-04	1432	2659	Up	1.25E-05	-0.51289	0.114523	4.902386
MA0471.2	E2F6	0.08	1583	3697	Down	4150908	9.32E-06	966	2179	Down	1.39E-05	-0.94031	-0.16601	-4.85664
MA0893.2	GSX2	0.05	4649	8340	Up	4147799	1.18E-05	1540	2892	Up	1.45E-05	-0.39988	0.098224	4.838767

MA0024.3	E2F1	0.14	5047	11470	Down	4144735	1.47E-05	1208	2652	Down	1.76E-05	-0.63745	-0.12699	-4.75367
MA0702.2	LMX1A	0.13	3876	6894	Up	4114136	1.24E-04	1328	2447	Up	2.10E-05	-0.62718	0.125632	4.678362
MA1099.2	HES1	0.03	815	2052	Down	4094302	4.37E-04	542	1303	Down	2.31E-05	-1.72769	-0.25755	-4.63706
MA0792.1	POU5F1B	0.15	3571	6652	Up	4103842	2.42E-04	1474	2760	Up	3.53E-05	-0.46516	0.102439	4.451837
MA0632.2	TCFL5	0.1	5478	12500	Down	4126359	5.46E-05	1333	2884	Down	3.75E-05	-0.50596	-0.10596	-4.42621
MA0899.1	HOXA10	0.09	5575	10070	Up	4174758	1.45E-06	1679	3206	Up	3.77E-05	-0.26322	0.074175	4.423972
MA0849.1	FOXO6	0.15	3186	5763	Up	4127576	5.02E-05	1464	2740	Up	3.89E-05	-0.47531	0.103112	4.41008
MA1102.2	CTCFL	0.15	5649	12116	Down	4079015	0.001078	1702	3563	Down	4.58E-05	-0.17726	-0.0585	-4.33956
MA0125.1	Nobox	0.07	4015	7272	Up	4096058	3.93E-04	1397	2600	Up	4.64E-05	-0.54691	0.111202	4.333864
MA0867.2	SOX4	0.15	15997	31093	Up	4016636	0.023817	1963	3916	Up	4.64E-05	-0.00624	0.011005	4.333092
MA1512.1	KLF11	0.07	647	1595	Down	4080013	0.001018	517	1241	Down	5.14E-05	-1.79688	-0.25531	-4.2892
MA0700.2	LHX2	0.14	3864	6901	Up	4112185	1.41E-04	1355	2516	Up	6.11E-05	-0.59261	0.114549	4.214207
MA0608.1	Creb3l2	0.13	1696	4095	Down	4107253	1.94E-04	756	1736	Down	6.35E-05	-1.28094	-0.19163	-4.19746
MA1115.1	POU5F1	0.09	1295	2329	Up	4075439	0.001321	912	1617	Up	6.57E-05	-1.1969	0.181273	4.182557
MA0506.1	NRF1	0.12	1829	4163	Down	4077740	0.00116	741	1703	Down	6.86E-05	-1.30922	-0.19284	-4.16386
MA0720.1	Shox2	0.04	4865	8775	Up	4121807	7.45E-05	1506	2836	Up	7.11E-05	-0.43008	0.094231	4.148
MA0619.1	LIN54	0.05	3154	5547	Up	4140673	1.98E-05	1369	2547	Up	7.13E-05	-0.57637	0.11171	4.147077
MA0724.1	VENTX	0.07	1604	2833	Up	4106674	2.02E-04	1041	1877	Up	7.84E-05	-0.994	0.156992	4.105733
MA0641.1	ELF4	0.13	349	901	Down	4039189	0.008667	296	758	Down	8.36E-05	-2.55407	-0.34797	-4.07787
MA0790.1	POU4F1	0.15	1141	1907	Up	4076908	0.001216	649	1107	Up	8.51E-05	-1.71538	0.237237	4.070213
MA0786.1	POU3F1	0.15	2846	5209	Up	4096504	3.82E-04	1311	2429	Up	8.69E-05	-0.64179	0.117701	4.060864
MA0151.1	Arid3a	0.04	4754	8370	Up	4175324	1.38E-06	1595	3031	Up	8.97E-05	-0.34071	0.081119	4.047247
MA1478.1	DMRTA2	0.07	2929	5366	Up	4116027	1.10E-04	1448	2716	Up	9.43E-05	-0.48958	0.099953	4.025268
MA1569.1	TFAP2E	0.09	12930	28472	Down	4130447	4.11E-05	1754	3648	Down	9.87E-05	-0.13855	-0.0491	-4.00556
MA0610.1	DMRT3	0.06	1277	2223	Up	4096373	3.85E-04	901	1601	Up	1.02E-04	-1.21282	0.178118	3.990008
MA0523.1	TCF7L2	0.15	4992	9132	Up	4130128	4.20E-05	1727	3323	Up	1.06E-04	-0.21704	0.063125	3.97634
MA0754.1	CUX1	0.1	2315	4107	Up	4122038	7.33E-05	1188	2179	Up	1.06E-04	-0.79116	0.132287	3.97365
MA0068.2	PAX4	0.06	3110	5479	Up	4109161	1.72E-04	1181	2165	Up	1.08E-04	-0.80007	0.133062	3.966584
MA0259.1	ARNT::HIF1A	0.08	8404	18451	Down	4174901	1.43E-06	1826	3768	Down	1.10E-04	-0.0862	-0.03777	-3.96048
MA0479.1	FOXH1	0.15	2044	3626	Up	4109012	1.74E-04	1217	2238	Up	1.10E-04	-0.75451	0.128532	3.958108

MA0747.1	SP8	0.14	7416	16347	Down	4101696	2.77E-04	1713	3574	Down	1.32E-04	-0.17039	-0.05365	-3.87987
MA0108.2	TBP	0.15	14069	26054	Up	4155778	6.45E-06	1866	3648	Up	1.38E-04	-0.09391	0.040175	3.859873
MA0850.1	FOXP3	0.11	5777	10547	Up	4150753	9.43E-06	1770	3423	Up	1.41E-04	-0.17792	0.055827	3.851627
MA0616.2	HES2	0.1	1421	3438	Down	4093805	4.51E-04	690	1589	Down	1.44E-04	-1.41059	-0.19572	-3.84304
MA0032.2	FOXC1	0.14	6655	12438	Up	4087153	6.72E-04	1713	3295	Up	1.44E-04	-0.22901	0.063591	3.841767
MA0649.1	HEY2	0.14	1825	4370	Down	4113477	1.30E-04	850	1916	Down	1.50E-04	-1.1253	-0.16495	-3.82523
MA0672.1	NKX2-3	0.06	4998	9470	Up	4091541	5.17E-04	1812	3522	Up	1.64E-04	-0.14044	0.048524	3.784585
MA0791.1	POU4F3	0.15	831	1397	Up	4050719	0.004931	562	949	Up	1.64E-04	-1.93015	0.251828	3.783886
MA0033.2	FOXL1	0.14	9376	17567	Up	4102435	2.64E-04	1839	3584	Up	1.65E-04	-0.11719	0.044684	3.782956
MA0642.1	EN2	0.04	2068	3745	Up	4076856	0.001219	1081	1967	Up	1.71E-04	-0.93303	0.143813	3.766613
MA0695.1	ZBTB7C	0.12	3261	7099	Down	4108716	1.77E-04	1497	3177	Down	1.88E-04	-0.35251	-0.07819	-3.72645
MA0713.1	PHOX2A	0.1	677	1129	Up	4047174	0.005885	512	856	Up	1.89E-04	-2.07166	0.266241	3.724319
MA1511.1	KLF10	0.12	4709	10403	Down	4088056	6.37E-04	1598	3363	Down	1.97E-04	-0.26439	-0.0661	-3.70524
MA0507.1	POU2F2	0.12	1139	2092	Up	4053142	0.004363	837	1484	Up	2.12E-04	-1.32066	0.181327	3.674368
MA1113.2	PBX2	0.13	1650	3005	Up	4065084	0.002336	939	1687	Up	2.19E-04	-1.14531	0.16222	3.658683
MA0889.1	GBX1	0.06	3731	6700	Up	4092648	4.83E-04	1329	2478	Up	2.23E-04	-0.61756	0.108559	3.652538
MA0470.2	E2F4	0.15	1377	3137	Down	4078164	0.001132	825	1858	Down	2.51E-04	-1.16899	-0.16366	-3.60059
MA0468.1	DUX4	0.13	817	1404	Up	4060412	0.002995	633	1090	Up	2.65E-04	-1.74453	0.223569	3.576683
MA0084.1	SRY	0.06	6044	11254	Up	4108706	1.77E-04	1711	3297	Up	2.65E-04	-0.22942	0.061031	3.576399
MA0634.1	ALX3	0.04	3544	6391	Up	4074227	0.001414	1280	2380	Up	2.73E-04	-0.67375	0.112585	3.564281
MA0725.1	VSX1	0.08	3032	5333	Up	4093778	4.51E-04	1129	2073	Up	3.15E-04	-0.86386	0.130759	3.502128
MA0823.1	HEY1	0.13	1757	4199	Down	4099491	3.18E-04	800	1803	Down	3.33E-04	-1.21285	-0.16468	-3.47797
MA0726.1	VSX2	0.11	3355	5926	Up	4095736	4.00E-04	1203	2225	Up	3.42E-04	-0.76705	0.120249	3.465735
MA0880.1	Dlx3	0.11	10639	19557	Up	4157966	5.45E-06	1855	3629	Up	3.50E-04	-0.10194	0.03918	3.456343
MA0787.1	POU3F2	0.11	426	684	Up	4015618	0.024858	355	571	Up	3.55E-04	-2.62736	0.322239	3.449812
MA0017.2	NR2F1	0.05	896	2091	Down	4073361	0.001484	702	1601	Down	3.60E-04	-1.39274	-0.18171	-3.44371
MA0699.1	LBX2	0.05	3430	6147	Up	4080688	9.80E-04	1260	2343	Up	3.62E-04	-0.6964	0.112474	3.440886
MA0481.3	FOXP1	0.15	3935	7221	Up	4101899	2.73E-04	1546	2943	Up	3.82E-04	-0.38446	0.078617	3.418421
MA0905.1	HOXC10	0.15	5586	10304	Up	4117984	9.63E-05	1727	3336	Up	3.90E-04	-0.21422	0.057493	3.408917
MA0769.2	TCF7	0.01	439	708	Up	4022830	0.018261	382	621	Up	4.09E-04	-2.51405	0.306848	3.388221

MA0630.1	SHOX	0.02	3388	6060	Up	4079741	0.001034	1248	2320	Up	4.17E-04	-0.71042	0.112903	3.379349
MA1498.1	HOXA7	0.03	3447	6328	Up	4104172	2.37E-04	1456	2753	Up	4.52E-04	-0.47585	0.088382	3.344382
MA0715.1	PROP1	0.15	585	971	Up	4031112	0.012622	460	768	Up	4.53E-04	-2.22704	0.268298	3.343848
MA1640.1	MEIS2(var.2)	0.14	1956	3542	Up	4078603	0.001104	1044	1907	Up	4.53E-04	-0.98049	0.138272	3.343778
MA1596.1	ZNF460	0.11	1650	3993	Down	4106437	2.05E-04	811	1820	Down	4.65E-04	-1.19623	-0.15853	-3.33292
MA0614.1	Foxj2	0.12	4377	7927	Up	4146343	1.31E-05	1613	3089	Up	4.79E-04	-0.31895	0.069963	3.319584
MA0755.1	CUX2	0.13	2407	4376	Up	4083564	8.29E-04	1208	2241	Up	5.14E-04	-0.75889	0.115895	3.288934
MA0019.1	Ddit3::Cebpa	0.03	253	675	Down	4009569	0.031894	237	609	Down	5.15E-04	-2.87188	-0.35256	-3.28827
MA0601.1	Arid3b	0.09	7472	13322	Up	4177290	1.18E-06	1712	3307	Up	5.19E-04	-0.22681	0.057505	3.28466
MA0910.2	HOXD8	0.08	7003	12855	Up	4130757	4.02E-05	1739	3366	Up	5.36E-04	-0.20277	0.054566	3.270596
MA1637.1	EBF3	0.1	1835	4255	Down	4079603	0.001043	953	2105	Down	5.52E-04	-0.975	-0.13572	-3.25836
MA0833.2	ATF4	0.1	481	776	Up	4024842	0.016719	404	666	Up	5.73E-04	-2.42329	0.286677	3.242213
MA0890.1	GBX2	0.05	3172	5712	Up	4062734	0.002648	1196	2219	Up	5.86E-04	-0.77321	0.115728	3.232116
MA0846.1	FOXC2	0.15	8359	15687	Up	4108158	1.83E-04	1808	3524	Up	5.88E-04	-0.14163	0.044518	3.230772
MA0884.1	DUXA	0.14	2258	4071	Up	4087105	6.74E-04	1227	2283	Up	6.05E-04	-0.73425	0.111619	3.218119
MA0465.2	CDX2	0.14	1985	3624	Up	4093097	4.70E-04	1169	2164	Up	6.15E-04	-0.80777	0.119001	3.211216
MA0124.2	Nkx3-1	0.11	3458	6551	Up	4054062	0.004163	1622	3111	Up	6.20E-04	-0.30982	0.067751	3.207855
MA0031.1	FOXD1	0.15	3841	6948	Up	4135378	2.90E-05	1530	2915	Up	6.25E-04	-0.39886	0.077402	3.204177
MA1502.1	HOXB8	0.08	7058	12975	Up	4129035	4.53E-05	1741	3374	Up	6.80E-04	-0.20023	0.052799	3.16756
MA0902.2	HOXB2	0.03	2606	4670	Up	4054001	0.004176	1030	1884	Up	6.80E-04	-0.99897	0.136305	3.167373
MA0760.1	ERF	0.15	1216	2729	Down	4063350	0.002563	825	1843	Down	7.10E-04	-1.17484	-0.15196	-3.1485
MA0078.1	Sox17	0.05	3692	6944	Up	4058490	0.003312	1565	2991	Up	7.15E-04	-0.36399	0.072897	3.145399
MA1636.1	CEBPG(var.2)	0.14	740	1289	Up	4035769	0.010181	598	1037	Up	7.66E-04	-1.82147	0.213454	3.11588
MA1505.1	HOXC8	0.1	11984	21900	Up	4117529	9.93E-05	1660	3197	Up	7.93E-04	-0.27345	0.061816	3.100767
MA1476.1	DLX5	0.02	2868	5218	Up	4051811	0.004667	1153	2137	Up	8.54E-04	-0.82676	0.117236	3.068699
MA0599.1	KLF5	0.08	11324	24461	Down	4029252	0.013733	1813	3734	Down	8.54E-04	-0.09789	-0.035	-3.06833
MA1474.1	CREB3L4	0.14	1202	2762	Down	4080112	0.001013	821	1831	Down	8.66E-04	-1.18305	-0.14955	-3.06266
MA1463.1	ARGFX	0.12	4744	8744	Up	4086753	6.88E-04	1541	2943	Up	8.91E-04	-0.3868	0.073945	3.050212
MA0894.1	HESX1	0.02	3086	5552	Up	4057576	0.003473	1174	2181	Up	9.14E-04	-0.79905	0.113869	3.039086
MA0718.1	RAX	0.02	3099	5584	Up	4055061	0.003955	1174	2181	Up	9.14E-04	-0.79905	0.113869	3.039086

MA0886.1	EMX2	0.09	3099	5584	Up	4055061	0.003955	1174	2181	Up	9.14E-04	-0.79905	0.113869	3.039086
MA0662.1	MIXL1	0.02	3099	5584	Up	4055061	0.003955	1174	2181	Up	9.14E-04	-0.79905	0.113869	3.039086
MA0644.1	ESX1	0.02	3099	5584	Up	4055061	0.003955	1174	2181	Up	9.14E-04	-0.79905	0.113869	3.039086
MA0707.1	MNX1	0.07	3099	5584	Up	4055061	0.003955	1174	2181	Up	9.14E-04	-0.79905	0.113869	3.039086
MA0654.1	ISX	0.05	3288	5881	Up	4072367	0.001569	1219	2272	Up	9.21E-04	-0.74245	0.109152	3.035824
MA1656.1	ZNF449	0.15	1708	3653	Down	4023223	0.01795	1055	2300	Down	0.001005	-0.8378	-0.11688	-2.99781
MA0626.1	Npas2	0.14	1772	4187	Down	4087181	6.71E-04	824	1835	Down	0.001055	-1.17885	-0.14744	-2.97683
MA1479.1	DMRTC2	0.13	1161	2010	Up	4069610	0.001826	809	1452	Up	0.001085	-1.36091	0.163707	2.964681
MA0030.1	FOXF2	0.13	2134	3830	Up	4095153	4.15E-04	1184	2203	Up	0.00109	-0.7857	0.111624	2.962602
MA0612.2	EMX1	0.08	3102	5592	Up	4054165	0.004141	1175	2185	Up	0.001107	-0.79711	0.112454	2.955931
MA0881.1	Dlx4	0.02	3102	5592	Up	4054165	0.004141	1175	2185	Up	0.001107	-0.79711	0.112454	2.955931
MA0882.1	DLX6	0.01	3102	5592	Up	4054165	0.004141	1175	2185	Up	0.001107	-0.79711	0.112454	2.955931
MA0666.1	MSX1	0.05	3102	5592	Up	4054165	0.004141	1175	2185	Up	0.001107	-0.79711	0.112454	2.955931
MA0027.2	EN1	0.02	3102	5592	Up	4054165	0.004141	1175	2185	Up	0.001107	-0.79711	0.112454	2.955931
MA0716.1	PRRX1	0.01	3102	5592	Up	4054165	0.004141	1175	2185	Up	0.001107	-0.79711	0.112454	2.955931
MA0721.1	UNCX	0.02	3102	5592	Up	4054165	0.004141	1175	2185	Up	0.001107	-0.79711	0.112454	2.955931
MA0709.1	Msx3	0.04	3102	5592	Up	4054165	0.004141	1175	2185	Up	0.001107	-0.79711	0.112454	2.955931
MA1577.1	TLX2	0.01	3102	5592	Up	4054165	0.004141	1175	2185	Up	0.001107	-0.79711	0.112454	2.955931
MA0717.1	RAX2	0.01	3102	5592	Up	4054165	0.004141	1175	2185	Up	0.001107	-0.79711	0.112454	2.955931
MA1518.1	LHX1	0.03	3102	5592	Up	4054165	0.004141	1175	2185	Up	0.001107	-0.79711	0.112454	2.955931
MA0058.3	MAX	0.01	769	1930	Down	4044566	0.006689	451	1061	Down	0.001117	-2.00828	-0.22616	-2.95179
MA0603.1	Arntl	0.15	3013	6855	Down	4129271	4.46E-05	1281	2742	Down	0.001123	-0.57107	-0.09052	-2.9496
MA0848.1	FOXO4	0.11	2452	4436	Up	4092483	4.88E-04	1287	2415	Up	0.001139	-0.65929	0.099393	2.943411
MA0788.1	POU3F3	0.13	436	721	Up	4006086	0.036672	365	601	Up	0.001151	-2.57044	0.288449	2.938891
MA1578.1	VEZF1	0.08	7600	17429	Down	4071648	0.001632	1644	3430	Down	0.001161	-0.2297	-0.05363	-2.93512
MA1683.1	FOXA3	0.14	3708	6787	Up	4089922	5.70E-04	1507	2874	Up	0.001165	-0.42	0.075989	2.933555
MA0593.1	FOXP2	0.11	2342	4191	Up	4107691	1.89E-04	1242	2323	Up	0.001172	-0.71296	0.104089	2.930984
MA0903.1	HOXB3	0.02	3737	6714	Up	4078492	0.001111	1342	2530	Up	0.001262	-0.59556	0.092642	2.898778
MA0518.1	Stat4	0.14	5471	10622	Up	4040439	0.008165	1771	3447	Up	0.001281	-0.17247	0.046563	2.892341
MA1103.2	FOXK2	0.15	2995	5320	Up	4117937	9.66E-05	1385	2619	Up	0.001283	-0.54788	0.088258	2.89182

MA0876.1	BSX	0.03	5550	10101	Up	4112786	1.36E-04	1630	3138	Up	0.001296	-0.30004	0.062382	2.887247
MA0002.2	RUNX1	0.05	854	1519	Up	4035421	0.010348	692	1226	Up	0.001305	-1.59552	0.182481	2.884326
MA0673.1	NKX2-8	0.07	3993	7452	Up	4125180	5.92E-05	1686	3260	Up	0.001315	-0.24817	0.056082	2.881208
MA0722.1	VAX1	0.11	8170	14847	Up	4145139	1.43E-05	1726	3348	Up	0.001337	-0.21205	0.051478	2.873876
MA0789.1	POU3F4	0.11	644	1078	Up	4028178	0.014413	507	870	Up	0.001364	-2.06704	0.228706	2.86523
MA1496.1	HOXA4	0.11	8297	15244	Up	4126469	5.41E-05	1761	3426	Up	0.001371	-0.18096	0.047211	2.863001
MA0704.1	Lhx4	0.08	3797	6792	Up	4083528	8.31E-04	1342	2531	Up	0.001394	-0.59528	0.092072	2.855665
MA1639.1	MEIS1(var.2)	0.15	1249	2152	Up	4080469	9.92E-04	831	1500	Up	0.001413	-1.31812	0.155487	2.849963
MA0812.1	TFAP2B(var.2)	0.12	5294	11652	Down	4087004	6.78E-04	1466	3095	Down	0.001418	-0.38646	-0.07065	-2.84829
MA1604.1	Ebf2	0.15	2188	4962	Down	4068539	0.001937	1076	2336	Down	0.001439	-0.81238	-0.11086	-2.84179
MA0904.2	HOXB5	0.12	8610	15774	Up	4116106	1.09E-04	1679	3246	Up	0.001448	-0.25427	0.056289	2.839226
MA0627.2	POU2F3	0.15	1205	2248	Up	4031107	0.012625	865	1568	Up	0.001522	-1.25724	0.149363	2.817474
MA0613.1	FOXG1	0.08	2163	3883	Up	4091862	5.07E-04	1190	2222	Up	0.00159	-0.77586	0.106527	2.798676
MA0059.1	MAX::MYC	0.11	512	1316	Down	4023559	0.017688	344	828	Down	0.001623	-2.38216	-0.25886	-2.78961
MA1615.1	Plagl1	0.13	2971	6785	Down	4150778	9.41E-06	1332	2836	Down	0.001636	-0.51861	-0.08283	-2.78635
MA0104.4	MYCN	0.06	656	1662	Down	4007346	0.034877	296	724	Down	0.001672	-2.58715	-0.28181	-2.7768
MA0706.1	MEOX2	0.11	12239	22478	Up	4118754	9.15E-05	1684	3259	Up	0.001725	-0.24925	0.054812	2.763212
MA0622.1	Mlxip	0.01	617	1544	Down	4040072	0.008309	453	1060	Down	0.001729	-2.00577	-0.21843	-2.76209
MA0875.1	BARX1	0.04	4850	8909	Up	4112889	1.35E-04	1624	3129	Up	0.001742	-0.30477	0.061206	2.759047
MA0723.1	VAX2	0.1	6954	12725	Up	4112696	1.37E-04	1664	3215	Up	0.001756	-0.26767	0.057188	2.755379
MA0508.3	PRDM1	0.11	1486	2586	Up	4081917	9.13E-04	978	1795	Up	0.001769	-1.07122	0.131401	2.752362
MA0851.1	Foxj3	0.15	3832	7031	Up	4080419	9.95E-04	1480	2825	Up	0.001916	-0.44544	0.074721	2.717573
MA1606.1	Foxf1	0.04	2156	3877	Up	4089742	5.76E-04	1188	2221	Up	0.001926	-0.7774	0.10475	2.715245
MA0785.1	POU2F1	0.15	2192	4096	Up	4054393	0.004093	1159	2162	Up	0.002008	-0.81463	0.107945	2.697192
MA0674.1	NKX6-1	0.03	1117	1902	Up	4048869	0.00541	697	1242	Up	0.002014	-1.58098	0.174162	2.696034
MA0658.1	LHX6	0.15	7381	13478	Up	4106636	2.02E-04	1642	3169	Up	0.002021	-0.28766	0.058781	2.694445
MA1530.1	NKX6-3	0.02	445	719	Up	4004739	0.038677	371	619	Up	0.002135	-2.53743	0.2694	2.670629
MA0618.1	LBX1	0.08	1659	2939	Up	4070159	0.001772	1016	1874	Up	0.002167	-1.01268	0.124247	2.664066
MA1549.1	POU6F1(var.2)	0.04	597	1021	Up	4016226	0.024231	494	851	Up	0.002184	-2.10167	0.223107	2.660828
MA1630.1	Znf281	0.15	2496	5463	Down	4051775	0.004676	1282	2733	Down	0.002315	-0.57288	-0.08465	-2.63546

MA0705.1	Lhx8	0.09	6218	11316	Up	4079998	0.001019	1413	2688	Up	0.002482	-0.5147	0.079613	2.605188
MA0006.1	Ahr::Arnt	0.01	2480	5201	Down	4002200	0.042711	1209	2589	Down	0.00249	-0.65419	-0.09112	-2.60381
MA0694.1	ZBTB7B	0.11	1025	2309	Down	4048672	0.005464	770	1711	Down	0.002513	-1.27817	-0.14425	-2.59973
MA1608.1	Isl1	0.14	5645	10115	Up	4173047	1.66E-06	1708	3316	Up	0.002564	-0.22654	0.050211	2.591038
MA0077.1	SOX9	0.08	1651	3097	Up	4025135	0.016504	1064	1974	Up	0.002602	-0.9419	0.115831	2.584675
MA0901.2	HOXB13	0.14	2613	4958	Up	4027139	0.015099	1299	2452	Up	0.002605	-0.64163	0.090846	2.584201
MA0661.1	MEOX1	0.13	8318	15203	Up	4129454	4.40E-05	1715	3331	Up	0.002624	-0.22033	0.0496	2.580973
MA0814.2	TFAP2C(var.2)	0.15	6417	14237	Down	4205509	1.06E-07	1789	3684	Down	0.002648	-0.11722	-0.03477	-2.57716
MA1104.2	GATA6	0.15	2942	5437	Up	4051513	0.004738	1413	2690	Up	0.002737	-0.51416	0.07854	2.562804
MA1499.1	HOXB4	0.09	7148	13113	Up	4109339	1.70E-04	1685	3266	Up	0.002741	-0.24727	0.052574	2.562134
MA0480.1	Foxo1	0.11	3476	6361	Up	4092198	4.97E-04	1546	2969	Up	0.002783	-0.37812	0.065929	2.555532
MA0741.1	KLF16	0.04	470	1169	Down	4015957	0.024507	380	898	Down	0.002827	-2.25195	-0.23248	-2.54862
MA1495.1	HOXA1	0.12	12261	22536	Up	4117591	9.89E-05	1682	3260	Up	0.002844	-0.24988	0.052656	2.546128
MA0900.2	HOXA2	0.09	6894	12617	Up	4109904	1.64E-04	1658	3210	Up	0.002989	-0.27139	0.054224	2.524419
MA0895.1	HMBOX1	0.14	5531	10264	Up	4077530	0.001174	1701	3304	Up	0.00318	-0.23211	0.049518	2.497598
MA0004.1	Arnt	0.1	3432	7610	Down	4107927	1.86E-04	1415	2986	Down	0.003235	-0.43785	-0.07	-2.49019
MA0912.2	HOXD3	0.14	4182	7614	Up	4082698	8.72E-04	1430	2728	Up	0.003364	-0.49542	0.075554	2.473111
MA0606.1	NFAT5	0.13	9730	18460	Up	4092847	4.78E-04	1923	3811	Up	0.003503	-0.04069	0.020517	2.455605
MA0132.2	PDX1	0.04	4581	8310	Up	4078655	0.001101	1470	2813	Up	0.003669	-0.4534	0.071083	2.435485
MA0887.1	EVX1	0.04	6887	12601	Up	4110093	1.62E-04	1657	3210	Up	0.003771	-0.27183	0.053353	2.423572
MA0888.1	EVX2	0.04	6887	12601	Up	4110093	1.62E-04	1657	3210	Up	0.003771	-0.27183	0.053353	2.423572
MA0147.3	MYC	0.06	587	1472	Down	4024526	0.016953	418	974	Down	0.003779	-2.12472	-0.21229	-2.42266
MA0847.2	FOXD2	0.14	2581	4973	Up	4032055	0.012089	1217	2291	Up	0.003791	-0.73763	0.094772	2.421261
MA1548.1	PLAGL2	0.05	1514	3463	Down	4054026	0.00417	831	1827	Down	0.003903	-1.1759	-0.12894	-2.40862
MA0892.1	GSX1	0.04	4576	8301	Up	4078451	0.001114	1469	2812	Up	0.004059	-0.45414	0.070615	2.391547
MA1560.1	SOHLH2	0.12	1438	3474	Down	4051912	0.004643	635	1426	Down	0.004123	-1.54851	-0.15937	-2.38478
MA1504.1	HOXC4	0.08	8034	14745	Up	4119297	8.82E-05	1742	3396	Up	0.004144	-0.19513	0.044252	2.382539
MA0852.2	FOXK1	0.12	2224	4022	Up	4080905	9.67E-04	1209	2276	Up	0.004196	-0.74712	0.094736	2.377155
MA0003.4	TFAP2A	0.03	430	1007	Down	4000461	0.045674	366	862	Down	0.00425	-2.30849	-0.22757	-2.37159
MA1106.1	HIF1A	0.11	3371	7470	Down	4102206	2.68E-04	1399	2951	Down	0.004368	-0.45455	-0.06939	-2.35974

MA0527.1	ZBTB33	0.14	1036	2316	Down	4009211	0.032358	634	1423	Down	0.004495	-1.55117	-0.15861	-2.34724
MA1107.2	KLF9	0.12	990	2202	Down	4023634	0.01763	705	1568	Down	0.004539	-1.40468	-0.14553	-2.34305
MA1120.1	SOX13	0.12	3158	5924	Up	4040404	0.008179	1446	2766	Up	0.004548	-0.47742	0.071647	2.342172
MA0874.1	Arx	0.13	1712	2996	Up	4024612	0.016889	664	1191	Up	0.004704	-1.64617	0.164705	2.327504
MA1497.1	HOXA6	0.04	1863	3384	Up	4040596	0.008104	962	1779	Up	0.004738	-1.08957	0.120529	2.324442
MA0515.1	Sox6	0.07	1749	3340	Up	4011028	0.030057	1142	2143	Up	0.005009	-0.83165	0.099368	2.300221
MA1471.1	BARX2	0.11	3160	5796	Up	4086771	6.87E-04	1346	2560	Up	0.005112	-0.58491	0.079931	2.291381
MA0464.2	BHLHE40	0.06	1276	3102	Down	4034091	0.011007	533	1211	Down	0.005338	-1.79256	-0.17609	-2.27262
MA1464.1	ARNT2	0.06	1276	3102	Down	4034091	0.011007	533	1211	Down	0.005338	-1.79256	-0.17609	-2.27262
MA0825.1	MNT	0.01	1276	3102	Down	4034091	0.011007	533	1211	Down	0.005338	-1.79256	-0.17609	-2.27262
MA0664.1	MLXIPL	0.07	1276	3102	Down	4034091	0.011007	533	1211	Down	0.005338	-1.79256	-0.17609	-2.27262
MA0793.1	POU6F2	0.15	6350	11553	Up	4107731	1.89E-04	1610	3117	Up	0.006424	-0.31378	0.054262	2.192173
MA0143.4	SOX2	0.14	3074	5801	Up	4023717	0.017567	1430	2738	Up	0.006424	-0.49278	0.070276	2.192169
MA0898.1	Hmx3	0.15	762	1325	Up	4015590	0.024887	584	1042	Up	0.007141	-1.83507	0.17237	2.146262
MA0158.2	HOXA5	0.1	7697	14104	Up	4124545	6.18E-05	1737	3392	Up	0.007157	-0.19805	0.041807	2.14529
MA1580.1	ZBTB32	0.12	1169	2157	Up	4014491	0.026056	836	1536	Up	0.007443	-1.29669	0.129932	2.128236
MA0676.1	Nr2e1	0.06	1124	2050	Up	4014401	0.026154	837	1539	Up	0.007472	-1.29442	0.128842	2.126544
MA1650.1	ZBTB14	0.09	955	2219	Down	4030424	0.013023	667	1481	Down	0.007596	-1.48578	-0.14308	-2.11941
MA0831.2	TFE3	0.13	2962	6604	Down	4073025	0.001512	1304	2755	Down	0.008807	-0.55483	-0.07167	-2.05516
MA0879.1	Dlx1	0.04	7202	13341	Up	4090926	5.36E-04	1723	3366	Up	0.009536	-0.20944	0.041234	2.02063
MA0056.2	MZF1	0.11	2572	5451	Down	4001480	0.043918	1371	2884	Down	0.009804	-0.4857	-0.06542	-2.00861
MA0137.3	STAT1	0.15	2661	5148	Up	4001880	0.043243	1405	2695	Up	0.010028	-0.51692	0.067673	1.998775
MA0042.2	FOXI1	0.15	3020	5540	Up	4074726	0.001375	1400	2685	Up	0.010203	-0.52217	0.067894	1.991283
MA0148.4	FOXA1	0.15	2805	5137	Up	4043337	0.007101	1335	2554	Up	0.012337	-0.59252	0.071482	1.90878
MA0493.1	Klf1	0.08	1147	2564	Down	4025281	0.016398	822	1788	Down	0.012351	-1.19931	-0.11352	-1.90828
MA1152.1	SOX15	0.03	1222	2282	Up	4001246	0.044316	871	1616	Up	0.012805	-1.23051	0.115842	1.892631
MA1135.1	FOSB::JUNB	0.14	3992	7597	Up	4032528	0.01183	1469	2832	Up	0.013105	-0.44903	0.060392	1.882567
MA1507.1	HOXD4	0.07	4306	7845	Up	4067176	0.002086	1443	2778	Up	0.013323	-0.47579	0.062407	1.875389
MA0753.2	ZNF740	0.15	2055	5009	Down	4036966	0.009626	760	1661	Down	0.013479	-1.30898	-0.12032	-1.87035
MA1138.1	FOSL2::JUNB	0.15	4523	8678	Up	4032290	0.01196	1550	3002	Up	0.013858	-0.36828	0.053712	1.858313

MA0140.2	GATA1::TAL1	0.11	813	1489	Up	3998336	0.049529	651	1185	Up	0.014445	-1.66407	0.143484	1.840273
MA0157.2	FOXO3	0.14	1873	3359	Up	4051268	0.004797	1102	2082	Up	0.0148	-0.87819	0.089605	1.829737
MA0101.1	REL	0.08	2405	5115	Down	3999913	0.046642	1311	2758	Down	0.015768	-0.55018	-0.06552	-1.80224
MA0131.2	HINFP	0.14	1676	3614	Down	4013645	0.026988	986	2114	Down	0.016159	-0.94738	-0.09278	-1.79157
MA0853.1	Alx4	0.12	1547	2741	Up	3998753	0.048751	610	1107	Up	0.016301	-1.76005	0.147899	1.787778
MA0047.3	FOXA2	0.15	2833	5292	Up	4018820	0.021709	1372	2635	Up	0.016698	-0.55029	0.065872	1.777348
MA1525.1	NFATC4	0.05	3734	7120	Up	4028511	0.014199	1634	3183	Up	0.017229	-0.288	0.045378	1.763752
MA0476.1	FOS	0.13	2375	4443	Up	4012807	0.02794	1001	1882	Up	0.017529	-1.02033	0.096655	1.756237
MA0675.1	NKX6-2	0.02	2425	4243	Up	4032317	0.011945	1005	1891	Up	0.018886	-1.01401	0.095525	1.723851
MA0648.1	GSC	0.09	7464	14226	Up	4046307	0.006142	1834	3619	Up	0.020254	-0.11214	0.026739	1.693495
MA0482.2	GATA4	0.14	2435	4576	Up	4001906	0.0432	1315	2523	Up	0.020674	-0.61221	0.067328	1.684567
MA1649.1	ZBTB12	0.01	625	1425	Down	3999269	0.047803	534	1189	Down	0.020923	-1.80442	-0.14695	-1.67937
MA0162.4	EGR1	0.15	9042	19522	Down	4025420	0.016298	1712	3523	Down	0.021498	-0.18118	-0.03376	-1.66761
MA0624.1	NFATC1	0.01	3101	5834	Up	4041153	0.007891	1504	2914	Up	0.022147	-0.41147	0.053178	1.654676
MA0489.1	JUN(var.2)	0.15	3281	6190	Up	4032741	0.011715	1308	2510	Up	0.022585	-0.61979	0.067082	1.646175
MA1562.1	SOX14	0.15	1835	3453	Up	4019678	0.020928	1115	2117	Up	0.02295	-0.85771	0.082471	1.63922
MA1508.1	IKZF1	0.1	2534	4779	Up	4029019	0.013878	1382	2663	Up	0.023982	-0.53743	0.061099	1.620113
MA0845.1	FOXB1	0.14	1607	2970	Up	4012575	0.028208	993	1873	Up	0.025233	-1.02957	0.091998	1.598029
MA1607.1	Foxl2	0.15	2053	3798	Up	4038999	0.008745	1152	2194	Up	0.025835	-0.8084	0.078017	1.587792
MA0891.1	GSC2	0.1	7783	14922	Up	4023840	0.017472	1840	3635	Up	0.026595	-0.10661	0.025086	1.575202
MA0625.1	NFATC3	0.04	4947	9556	Up	4010229	0.03105	1771	3486	Up	0.031127	-0.16436	0.030335	1.506861
MA0087.1	Sox5	0.14	5437	10156	Up	4081062	9.59E-04	1707	3350	Up	0.034062	-0.2196	0.034652	1.467725
MA0751.1	ZIC4	0.15	2369	5340	Down	4070006	0.001787	1168	2460	Down	0.035888	-0.71593	-0.06715	-1.44505
MA1134.1	FOS::JUNB	0.14	2419	4511	Up	4008255	0.03363	1013	1923	Up	0.038383	-0.99619	0.082755	1.415859
MA1535.1	NR2C1	0.13	8919	18899	Down	4066052	0.002217	1922	3893	Down	0.038648	-0.02571	-0.01094	-1.41287
MA0633.1	Twist2	0.14	6097	11371	Up	4020716	0.020014	1602	3131	Up	0.041488	-0.31414	0.040613	1.382078
MA0152.1	NFATC2	0.07	5499	10587	Up	4014729	0.025799	1789	3529	Up	0.04323	-0.14822	0.027235	1.364215
MA1644.1	NFYC	0.11	1509	2737	Up	4016485	0.023968	969	1840	Up	0.049651	-1.06003	0.082358	1.304075

Supplementary Table S8. Summary of over- and under-represented DNA binding sites for transcription factors (TFs) in genes with high variability (p values <0.01) vs. low variability (p>0.7) found in hippocampus of stressed rats vs. control groups using CiiiDer TFMs software [S4] (Experiment 1). Only TFs with p<0.05 simultaneously for gene coverage p-value and the number of the binding sites distributions were considered significant. TFs in the table are listed as sorted by their gene coverage p-value.

TF ID	TF Name	Deficit	N sites	N background sites	Direction	MW U	P-Value	N TF (of 1964)	N TF background genes (of 3948)	Direction	P-Value	Average Log2 Proportion Bound	Log2 Enrichment	Significance Score
MA0076.2	ELK4	0.09	711	2974	Down	5018265	0.00E+00	532	1964	Down	4.10E-20	-1.54124	-0.50384	-19.3868
MA0759.1	ELK3	0.1	818	3342	Down	5030594	0.00E+00	602	2136	Down	7.65E-19	-1.39161	-0.44674	-18.1162
MA0028.2	ELK1	0.11	538	2267	Down	4933371	5.95E-13	429	1633	Down	7.45E-18	-1.8294	-0.54776	-17.128
MA0474.2	ERG	0.08	488	2110	Down	4914055	4.33E-12	393	1520	Down	2.03E-17	-1.94426	-0.57064	-16.6927
MA0750.2	ZBTB7A	0.05	456	1922	Down	4888902	5.14E-11	375	1455	Down	1.04E-16	-2.00955	-0.57516	-15.9822
MA0156.2	FEV	0.08	561	2330	Down	4919559	2.48E-12	445	1656	Down	1.41E-16	-1.79293	-0.51517	-15.8506
MA0098.3	ETS1	0.08	381	1673	Down	4860296	7.35E-10	323	1293	Down	2.50E-16	-2.20219	-0.61997	-15.6024
MA0765.2	ETV5	0.02	265	1284	Down	4813324	4.07E-08	237	1020	Down	8.96E-16	-2.59611	-0.7238	-15.0476
MA1483.1	ELF2	0.15	681	2730	Down	4941597	2.50E-13	531	1885	Down	1.09E-15	-1.5722	-0.44733	-14.9613
MA0645.1	ETV6	0.04	250	1218	Down	4801841	1.02E-07	223	973	Down	1.42E-15	-2.67395	-0.74343	-14.8479
MA0475.2	FLI1	0.12	963	3688	Down	4988165	1.44E-15	685	2299	Down	2.48E-15	-1.24547	-0.36662	-14.6062
MA0760.1	ERF	0.12	360	1629	Down	4843413	3.27E-09	313	1241	Down	5.29E-15	-2.25444	-0.60607	-14.2767
MA1484.1	ETS2	0.1	594	2330	Down	4893736	3.23E-11	474	1705	Down	1.19E-14	-1.72641	-0.46624	-13.9237
MA0763.1	ETV3	0.14	810	3165	Down	4953386	7.03E-14	604	2065	Down	1.42E-14	-1.4136	-0.3932	-13.8478
MA0641.1	ELF4	0.15	422	1767	Down	4837655	5.37E-09	359	1352	Down	1.61E-13	-2.0939	-0.53209	-12.794
MA0506.1	NRF1	0.06	183	846	Down	4689608	1.98E-04	130	622	Down	1.43E-12	-3.38461	-0.87455	-11.8436
MA0762.1	ETV2	0.13	290	1238	Down	4749076	4.86E-06	258	1008	Down	3.05E-11	-2.54352	-0.5845	-10.5159
MA0748.2	YY2	0.08	718	2432	Down	4786319	3.36E-07	563	1836	Down	3.47E-09	-1.54903	-0.325	-8.45967
MA0862.1	GMEB2	0.13	2825	9070	Down	4880307	1.16E-10	1120	3253	Down	7.68E-08	-0.64068	-0.15838	-7.11484
MA0740.1	KLF14	0.13	1040	3321	Down	4756633	2.89E-06	656	2052	Down	8.58E-08	-1.35862	-0.26504	-7.06644
MA0079.4	SP1	0.1	535	1807	Down	4696253	1.35E-04	371	1257	Down	2.03E-07	-2.12275	-0.37965	-6.6934
MA0685.1	SP4	0.09	279	995	Down	4639331	0.002666	212	782	Down	5.74E-07	-2.8679	-0.50115	-6.24137

MA0746.2	SP3	0.08	554	1914	Down	4700746	1.04E-04	403	1330	Down	9.48E-07	-2.02244	-0.34185	-6.02319
MA0057.1	MZF1(var.2)	0.06	4179	9802	Up	4757861	2.65E-06	1576	3863	Up	1.89E-06	-0.27043	0.08629	5.722496
MA0136.2	ELF5	0.04	487	1694	Down	4695259	1.43E-04	408	1334	Down	2.16E-06	-2.01139	-0.32842	-5.66486
MA1472.1	BHLHA15(var.2)	0.09	7838	18424	Up	4790830	2.38E-07	1639	4050	Up	2.27E-06	-0.20807	0.074629	5.643976
MA0497.1	MEF2C	0.13	2275	6578	Down	4713916	4.73E-05	1135	3247	Down	2.83E-06	-0.63242	-0.13653	-5.54867
MA0615.1	Gmeb1	0.08	2129	6874	Down	4778384	6.07E-07	834	2475	Down	3.65E-06	-1.0504	-0.18926	-5.43809
MA0895.1	HMBOX1	0.15	7184	20336	Down	4805714	7.48E-08	1739	4647	Down	3.88E-06	-0.06618	-0.03831	-5.41097
MA0131.2	HINFP	0.14	1017	3113	Down	4711921	5.34E-05	663	2023	Down	4.08E-06	-1.36124	-0.22921	-5.38983
MA0604.1	Atf1	0.03	311	1148	Down	4642966	0.002245	257	894	Down	4.96E-06	-2.63285	-0.41704	-5.30484
MA0710.1	NOTO	0.07	3251	9295	Down	4724371	2.47E-05	1209	3419	Down	7.81E-06	-0.54965	-0.1199	-5.10744
MA0516.2	SP2	0.1	116	484	Down	4572662	0.040165	104	426	Down	8.14E-06	-3.81765	-0.64957	-5.08942
MA0831.2	TFE3	0.1	689	2271	Down	4695299	1.43E-04	534	1664	Down	8.37E-06	-1.65807	-0.25935	-5.07737
MA0638.1	CREB3	0.15	339	1207	Down	4647955	0.001766	298	1005	Down	9.84E-06	-2.44189	-0.37264	-5.00694
MA0527.1	ZBTB33	0.15	818	2653	Down	4685053	2.56E-04	499	1565	Down	1.17E-05	-1.75116	-0.26859	-4.93224
MA1592.1	ZNF274	0.15	714	1549	Up	4669911	5.81E-04	589	1273	Up	1.28E-05	-1.78057	0.268238	4.891214
MA1475.1	CREB3L4(var.2)	0.12	414	1489	Down	4609735	0.009842	204	727	Down	1.38E-05	-2.94815	-0.45137	-4.86065
MA1615.1	Plagl1	0.04	499	1038	Up	4640677	0.002502	433	896	Up	1.57E-05	-2.25551	0.331202	4.804354
MA0742.1	Klf12	0.07	181	721	Down	4591359	0.020396	154	575	Down	1.68E-05	-3.31947	-0.51774	-4.77405
MA0864.2	E2F2	0.15	4292	11854	Down	4676273	4.14E-04	1110	3160	Down	1.81E-05	-0.66807	-0.12947	-4.74297
MA1564.1	SP9	0.07	1467	4387	Down	4699629	1.11E-04	793	2343	Down	2.10E-05	-1.12626	-0.18289	-4.67678
MA0713.1	PHOX2A	0.14	1997	5808	Down	4731526	1.56E-05	971	2798	Down	3.36E-05	-0.85228	-0.14689	-4.47315
MA0718.1	RAX	0.04	4472	12511	Down	4699072	1.15E-04	1385	3829	Down	4.12E-05	-0.36997	-0.08728	-4.38518
MA1125.1	ZNF384	0.03	11212	33810	Down	4733191	1.40E-05	1229	3446	Down	4.50E-05	-0.53215	-0.10759	-4.34713
MA1467.1	ATOH1(var.2)	0.14	2436	5591	Up	4691206	1.81E-04	1060	2491	Up	4.81E-05	-0.87287	0.147203	4.318091
MA1516.1	KLF3	0.09	1753	5084	Down	4672673	5.02E-04	823	2409	Down	4.98E-05	-1.07946	-0.16942	-4.30301
MA0618.1	LBX1	0.14	5110	14036	Down	4653919	0.001318	1543	4200	Down	5.33E-05	-0.22537	-0.06488	-4.27345
MA0632.2	TCFL5	0.1	3454	9705	Down	4645237	0.002014	961	2762	Down	7.05E-05	-0.86908	-0.14314	-4.15212
MA0135.1	Lhx3	0.15	1106	3156	Down	4634933	0.003271	595	1797	Down	8.17E-05	-1.52467	-0.21434	-4.08753
MA0052.4	MEF2A	0.15	2374	6755	Down	4664857	7.56E-04	1144	3223	Down	8.70E-05	-0.63208	-0.11444	-4.06061
MA1463.1	ARGFX	0.11	2016	5797	Down	4662175	8.69E-04	808	2360	Down	8.81E-05	-1.10754	-0.1663	-4.05511

MA0816.1	Ascl2	0.07	1929	4442	Up	4668934	6.12E-04	878	2029	Up	9.85E-05	-1.15663	0.171463	4.006395
MA0594.2	HOXA9	0.06	636	2019	Down	4658200	0.001063	518	1585	Down	1.01E-04	-1.71507	-0.23305	-3.9957
MA0621.1	mix-a	0.15	3413	9667	Down	4698579	1.18E-04	1209	3384	Down	1.03E-04	-0.55707	-0.10506	-3.98909
MA0095.2	YY1	0.09	778	2341	Down	4659044	0.001019	612	1837	Down	1.13E-04	-1.48849	-0.20549	-3.94862
MA1515.1	KLF2	0.02	448	1521	Down	4629699	0.004153	351	1124	Down	1.14E-04	-2.24331	-0.29821	-3.94355
MA1517.1	KLF6	0.03	270	957	Down	4594888	0.017819	226	766	Down	1.20E-04	-2.83678	-0.3793	-3.91906
MA0508.3	PRDM1	0.01	1042	2320	Up	4668020	6.42E-04	774	1768	Up	1.26E-04	-1.34682	0.188284	3.897915
MA0146.2	Zfx	0.02	233	840	Down	4588285	0.0229	202	695	Down	1.38E-04	-2.98769	-0.40066	-3.85929
MA1129.1	FOSL1::JUN(var.2)	0.15	343	1239	Down	4577769	0.033589	172	607	Down	1.48E-04	-3.20094	-0.43681	-3.83078
MA1117.1	RELB	0.13	5382	13150	Up	4678355	3.70E-04	1734	4378	Up	1.52E-04	-0.11127	0.043557	3.817766
MA0041.1	Foxd3	0.09	1298	4158	Down	4690630	1.87E-04	719	2117	Down	1.65E-04	-1.27003	-0.17782	-3.78225
MA0060.3	NFYA	0.1	624	1977	Down	4648172	0.001748	499	1524	Down	1.98E-04	-1.7703	-0.23031	-3.70437
MA0048.2	NHLH1	0.09	2133	4880	Up	4657739	0.001088	837	1940	Up	2.74E-04	-1.22345	0.167206	3.562567
MA1651.1	ZFP42	0.15	780	2305	Down	4640743	0.002495	613	1824	Down	3.06E-04	-1.49243	-0.19289	-3.51445
MA0644.1	ESX1	0.05	4445	12503	Down	4706470	7.42E-05	1382	3794	Down	3.22E-04	-0.37815	-0.07716	-3.49252
MA0725.1	VSX1	0.1	5501	15590	Down	4712467	5.17E-05	1421	3889	Down	3.31E-04	-0.34025	-0.0727	-3.4805
MA0880.1	Dlx3	0.05	4780	13575	Down	4730972	1.62E-05	1420	3887	Down	3.34E-04	-0.34113	-0.07297	-3.47662
MA0879.1	Dlx1	0.03	4778	13561	Down	4729338	1.80E-05	1419	3885	Down	3.37E-04	-0.34201	-0.07324	-3.47195
MA0700.2	LHX2	0.14	3167	8706	Down	4637146	0.002952	1169	3263	Down	3.92E-04	-0.60759	-0.10105	-3.40695
MA1109.1	NEUROD1	0.15	4657	11026	Up	4768808	1.22E-06	1664	4179	Up	4.09E-04	-0.17454	0.051232	3.388413
MA0888.1	EVX2	0.03	4581	12739	Down	4662509	8.54E-04	1444	3942	Down	4.14E-04	-0.31891	-0.06907	-3.38328
MA0706.1	MEOX2	0.08	5585	15429	Down	4619238	0.006588	1422	3889	Down	4.26E-04	-0.33974	-0.07168	-3.37043
MA1657.1	ZNF652	0.14	682	2043	Down	4626456	0.004802	538	1617	Down	4.44E-04	-1.67336	-0.20727	-3.35248
MA0693.2	VDR	0.04	1680	4805	Down	4672311	5.11E-04	1063	2992	Down	4.48E-04	-0.73866	-0.11306	-3.34895
MA0726.1	VSX2	0.13	4681	13180	Down	4681140	3.17E-04	1361	3739	Down	4.60E-04	-0.39973	-0.07817	-3.33699
MA0844.1	XBP1	0.15	259	864	Down	4579369	0.031729	230	761	Down	4.64E-04	-2.82887	-0.3446	-3.33336
MA1627.1	Wt1	0.13	2713	6261	Up	4699409	1.13E-04	1256	3048	Up	4.67E-04	-0.60499	0.100785	3.330302
MA1471.1	BARX2	0.04	369	1165	Down	4592155	0.019788	302	965	Down	4.86E-04	-2.46157	-0.29487	-3.31301
MA0720.1	Shox2	0.04	3897	10895	Down	4688455	2.11E-04	1312	3616	Down	5.14E-04	-0.45029	-0.0828	-3.28941
MA1481.1	DRGX	0.03	3983	11034	Down	4663954	7.93E-04	1323	3643	Down	5.25E-04	-0.43891	-0.08149	-3.28012

MA0702.2	LMX1A	0.13	3114	8736	Down	4668617	6.22E-04	1133	3167	Down	5.25E-04	-0.65168	-0.10309	-3.27966
MA0738.1	HIC2	0.01	993	2307	Up	4618321	0.006853	764	1767	Up	5.78E-04	-1.3566	0.170352	3.237766
MA0068.2	PAX4	0.07	3157	8822	Down	4645870	0.001954	1189	3307	Down	6.11E-04	-0.5857	-0.09591	-3.21369
MA1644.1	NFYC	0.12	1642	4786	Down	4678803	3.61E-04	1018	2871	Down	6.29E-04	-0.79962	-0.11589	-3.20148
MA0485.2	HOXC9	0.12	714	2177	Down	4634818	0.003288	569	1693	Down	6.47E-04	-1.59986	-0.19277	-3.1893
MA0886.1	EMX2	0.1	4440	12468	Down	4693501	1.59E-04	1411	3855	Down	6.48E-04	-0.35167	-0.07022	-3.18829
MA1489.1	FOXN3	0.13	1036	3077	Down	4660694	9.37E-04	755	2186	Down	6.95E-04	-1.21168	-0.15365	-3.15778
MA0067.1	Pax2	0.01	553	1725	Down	4613263	0.008496	437	1333	Down	7.30E-04	-1.96246	-0.22839	-3.13651
MA0846.1	FOXC2	0.13	4557	12691	Down	4674256	4.61E-04	1506	4083	Down	7.46E-04	-0.26325	-0.05913	-3.12716
MA1619.1	Ptf1a(var.2)	0.05	4052	9414	Up	4687236	2.26E-04	1203	2916	Up	7.86E-04	-0.66801	0.102472	3.104684
MA0500.2	MYOG	0.03	4052	9414	Up	4687236	2.26E-04	1203	2916	Up	7.86E-04	-0.66801	0.102472	3.104684
MA1641.1	MYF5	0.07	4052	9414	Up	4687236	2.26E-04	1203	2916	Up	7.86E-04	-0.66801	0.102472	3.104684
MA1522.1	MAZ	0.03	2368	5652	Up	4632696	0.003624	1184	2867	Up	8.36E-04	-0.69171	0.103958	3.07773
MA0691.1	TFAP4	0.12	4054	9430	Up	4684892	2.58E-04	1203	2917	Up	8.68E-04	-0.66776	0.101977	3.06169
MA0521.1	Tcf12	0.09	4053	9422	Up	4685787	2.45E-04	1203	2917	Up	8.68E-04	-0.66776	0.101977	3.06169
MA1635.1	BHLHE22(var.2)	0.01	4054	9430	Up	4684892	2.58E-04	1203	2917	Up	8.68E-04	-0.66776	0.101977	3.06169
MA1508.1	IKZF1	0.13	4215	10151	Up	4691419	1.79E-04	1636	4108	Up	8.94E-04	-0.19914	0.051475	3.048845
MA0780.1	PAX3	0.15	606	1863	Down	4596867	0.016503	391	1202	Down	9.51E-04	-2.11719	-0.23948	-3.02181
MA1512.1	KLF11	0.06	250	856	Down	4570139	0.043796	219	721	Down	9.52E-04	-2.90307	-0.33731	-3.02127
MA1100.2	ASCL1	0.03	1785	4060	Up	4625870	0.004929	730	1689	Up	9.58E-04	-1.42197	0.169833	3.018852
MA1506.1	HOXD10	0.15	906	2711	Down	4641282	0.002432	681	1985	Down	9.74E-04	-1.3556	-0.16324	-3.01123
MA1653.1	ZNF148	0.11	2097	5070	Up	4607029	0.011	1108	2668	Up	9.98E-04	-0.79143	0.112053	3.00072
MA0851.1	Foxj3	0.15	3669	10249	Down	4621214	0.006048	1413	3854	Down	0.001046	-0.35084	-0.0678	-2.98031
MA0602.1	Arid5a	0.04	793	2477	Down	4614869	0.00794	449	1360	Down	0.001102	-1.92848	-0.21827	-2.95768
MA0901.2	HOXB13	0.11	1836	5281	Down	4639711	0.002619	1028	2887	Down	0.001124	-0.78857	-0.10981	-2.94913
MA0658.1	LHX6	0.13	2343	6529	Down	4622615	0.005689	945	2672	Down	0.001175	-0.90508	-0.11957	-2.92983
MA0125.1	Nobox	0.07	3343	9212	Down	4637743	0.002872	1225	3386	Down	0.001196	-0.54717	-0.08695	-2.9223
MA0031.1	FOXD1	0.15	3665	10011	Down	4604408	0.012236	1479	4012	Down	0.001287	-0.28894	-0.05992	-2.89058
MA0117.2	Mafb	0.06	730	1628	Up	4613078	0.008562	596	1357	Up	0.001294	-1.72598	0.193115	2.887972
MA0905.1	HOXC10	0.09	1222	3542	Down	4637701	0.002877	840	2398	Down	0.001343	-1.06802	-0.13334	-2.87183

MA1513.1	KLF15	0.15	5174	14296	Down	4634909	0.003275	1276	3512	Down	0.001348	-0.4914	-0.08083	-2.87024
MA0601.1	Arid3b	0.05	3239	9364	Down	4700704	1.04E-04	1194	3304	Down	0.001415	-0.58333	-0.08855	-2.84918
MA1503.1	HOXB9	0.11	841	2527	Down	4635107	0.003245	650	1895	Down	0.001453	-1.42265	-0.16348	-2.83775
MA1583.1	ZFP57	0.14	1850	5301	Down	4650043	0.001595	969	2729	Down	0.001524	-0.87177	-0.11385	-2.81693
MA0887.1	EVX1	0.03	4467	12382	Down	4644964	0.002041	1435	3901	Down	0.001538	-0.33096	-0.063	-2.81291
MA0882.1	DLX6	0.05	5059	14358	Down	4734572	1.28E-05	1466	3977	Down	0.00154	-0.30163	-0.06001	-2.81254
MA0528.2	ZNF263	0.02	1783	4151	Up	4657331	0.001111	1066	2566	Up	0.001555	-0.84741	0.112555	2.80824
MA0894.1	HESX1	0.05	4337	12068	Down	4679632	3.45E-04	1394	3800	Down	0.001637	-0.37078	-0.06697	-2.78602
MA1106.1	HIF1A	0.12	3972	11594	Down	4717029	3.90E-05	1402	3820	Down	0.001695	-0.36287	-0.06629	-2.77093
MA0075.3	PRRX2	0.06	3077	8446	Down	4623901	0.005377	1155	3202	Down	0.001848	-0.62989	-0.09121	-2.73329
MA0701.2	LHX9	0.08	3077	8446	Down	4623901	0.005377	1155	3202	Down	0.001848	-0.62989	-0.09121	-2.73329
MA0722.1	VAX1	0.11	6876	18919	Down	4706960	7.21E-05	1625	4353	Down	0.001938	-0.16222	-0.04182	-2.71273
MA0705.1	Lhx8	0.07	1837	5155	Down	4605337	0.011785	736	2117	Down	0.001942	-1.25319	-0.14413	-2.71168
MA0744.2	SCRT2	0.1	396	861	Up	4570980	0.042556	358	777	Up	0.001974	-2.49527	0.262604	2.704668
MA1577.1	TLX2	0.02	4391	12228	Down	4669327	5.99E-04	1406	3827	Down	0.002063	-0.35949	-0.06482	-2.68554
MA0033.2	FOXL1	0.13	7492	20822	Down	4683787	2.74E-04	1693	4508	Down	0.002137	-0.10742	-0.03317	-2.67014
MA0642.1	EN2	0.08	4405	12089	Down	4635620	0.003169	1467	3975	Down	0.002214	-0.3015	-0.0583	-2.65477
MA0743.2	SCRT1	0.14	914	2117	Up	4610032	0.009722	726	1695	Up	0.002321	-1.42338	0.156797	2.634313
MA1478.1	DMRTA2	0.06	2193	6136	Down	4626417	0.00481	1215	3349	Down	0.002349	-0.561	-0.08292	-2.62911
MA0472.2	EGR2	0.15	523	1561	Down	4582417	0.028429	398	1206	Down	0.002362	-2.10201	-0.2187	-2.62672
MA1629.1	Zic2	0.08	452	960	Up	4581800	0.029072	394	867	Up	0.002369	-2.34723	0.242635	2.625466
MA0666.1	MSX1	0.08	4933	13924	Down	4717942	3.69E-05	1465	3967	Down	0.00258	-0.30394	-0.05736	-2.58841
MA1630.1	Znf281	0.14	1882	4404	Up	4661098	9.18E-04	1096	2655	Up	0.002603	-0.8028	0.103396	2.584487
MA0889.1	GBX1	0.06	3088	8461	Down	4617840	0.006996	1162	3212	Down	0.002674	-0.62328	-0.08699	-2.57288
MA0047.3	FOXA2	0.14	2616	7284	Down	4644415	0.002095	1301	3561	Down	0.002721	-0.46742	-0.07284	-2.56535
MA1497.1	HOXA6	0.07	5034	13720	Down	4627668	0.004549	1520	4099	Down	0.002754	-0.25375	-0.05143	-2.56002
MA0708.1	MSX2	0.08	3237	8902	Down	4630401	0.004023	1198	3302	Down	0.002812	-0.58135	-0.08286	-2.55099
MA0612.2	EMX1	0.1	3937	10954	Down	4652361	0.001423	1356	3698	Down	0.002834	-0.41033	-0.06758	-2.54767
MA0662.1	MIXL1	0.04	5240	14729	Down	4708105	6.73E-05	1486	4016	Down	0.002924	-0.28482	-0.05455	-2.53409
MA0716.1	PRRX1	0.03	5242	14823	Down	4725057	2.36E-05	1483	4009	Down	0.003014	-0.28753	-0.05494	-2.52089

MA0471.2	E2F6	0.08	936	2586	Down	4583630	0.0272	619	1798	Down	0.003068	-1.49576	-0.15814	-2.51312
MA1623.1	Stat2	0.12	1329	3882	Down	4624985	0.005126	842	2387	Down	0.003076	-1.06962	-0.12328	-2.51197
MA0847.2	FOXD2	0.13	2030	5765	Down	4641734	0.00238	1044	2909	Down	0.0031	-0.77196	-0.09849	-2.50858
MA0108.2	TBP	0.08	2547	7050	Down	4622100	0.005818	1112	3082	Down	0.003156	-0.6848	-0.09082	-2.50089
MA0650.2	HOXA13	0.15	1431	4086	Down	4619531	0.006505	936	2630	Down	0.003289	-0.9234	-0.11051	-2.48287
MA0874.1	Arx	0.15	2225	6252	Down	4605851	0.011541	765	2182	Down	0.003474	-1.20352	-0.13203	-2.45915
MA0899.1	HOXA10	0.1	5879	16278	Down	4645690	0.001971	1634	4367	Down	0.003545	-0.15592	-0.03848	-2.4504
MA1487.1	FOXE1	0.15	2939	8418	Down	4653446	0.001349	1250	3429	Down	0.003604	-0.5235	-0.07602	-2.44319
MA1122.1	TFDP1	0.09	1563	4285	Down	4603722	0.012579	866	2445	Down	0.003689	-1.03205	-0.11738	-2.43307
MA1618.1	Ptf1a	0.15	8868	21053	Up	4780380	5.24E-07	1799	4612	Up	0.003769	-0.04718	0.021521	2.423774
MA0842.2	NRL	0.12	687	1560	Up	4589559	0.021832	568	1306	Up	0.003834	-1.78828	0.178999	2.41636
MA0042.2	FOXI1	0.09	1392	3942	Down	4624139	0.005321	905	2545	Down	0.003996	-0.97138	-0.11169	-2.39833
MA0039.4	KLF4	0.01	1162	2555	Up	4625762	0.004952	731	1719	Up	0.004136	-1.40829	0.146414	2.383414
MA1645.1	NKX2-2	0.15	3885	9405	Up	4617401	0.007129	1609	4054	Up	0.004294	-0.22068	0.046562	2.367097
MA1593.1	ZNF317	0.1	1020	2342	Up	4621002	0.006103	770	1820	Up	0.004555	-1.32965	0.139006	2.341525
MA1565.1	TBX18	0.14	3379	8391	Up	4590017	0.021459	1543	3871	Up	0.004796	-0.2842	0.052786	2.319087
MA0723.1	VAX2	0.11	6403	17438	Down	4666262	7.03E-04	1596	4273	Down	0.004906	-0.18858	-0.04103	-2.30926
MA0900.2	HOXA2	0.09	5875	16018	Down	4653819	0.001324	1551	4166	Down	0.005018	-0.2275	-0.0457	-2.29946
MA1495.1	HOXA1	0.09	8250	22546	Down	4631714	0.00379	1551	4166	Down	0.005018	-0.2275	-0.0457	-2.29946
MA0739.1	Hic1	0.06	2623	6435	Up	4600564	0.01427	1390	3453	Up	0.005057	-0.44192	0.067018	2.296124
MA1683.1	FOXA3	0.14	3501	9622	Down	4616199	0.007504	1454	3930	Down	0.005194	-0.31613	-0.05472	-2.28454
MA0721.1	UNCX	0.03	3096	8522	Down	4620132	0.006338	1158	3190	Down	0.00521	-0.63073	-0.08205	-2.28318
MA0853.1	Alx4	0.15	1774	4970	Down	4589864	0.021583	653	1879	Down	0.005229	-1.42544	-0.14462	-2.28156
MA0686.1	SPDEF	0.15	764	2302	Down	4609887	0.009781	590	1710	Down	0.00523	-1.56653	-0.15494	-2.28151
MA1144.1	FOSL2::JUND	0.06	959	2291	Up	4569399	0.044912	539	1239	Up	0.005353	-1.86402	0.17941	2.271384
MA1099.2	HES1	0.15	11227	32315	Down	4720895	3.07E-05	1546	4152	Down	0.005422	-0.23225	-0.0455	-2.26585
MA0912.2	HOXD3	0.15	4756	13297	Down	4677428	3.89E-04	1504	4051	Down	0.00553	-0.26988	-0.0497	-2.25728
MA1519.1	LHX5	0.09	4777	13360	Down	4697832	1.24E-04	1459	3941	Down	0.005549	-0.31164	-0.0538	-2.2558
MA1500.1	HOXB6	0.05	4451	12072	Down	4618133	0.006909	1447	3911	Down	0.005561	-0.32311	-0.05469	-2.25485
MA0907.1	HOXC13	0.15	791	2272	Down	4590533	0.021045	626	1804	Down	0.005806	-1.48526	-0.14674	-2.23614

MA0699.1	LBX2	0.06	4303	11873	Down	4647446	0.00181	1387	3763	Down	0.005859	-0.38147	-0.06011	-2.23217
MA0040.1	Foxq1	0.12	1885	5181	Down	4607779	0.010668	1076	2978	Down	0.00592	-0.73328	-0.08876	-2.22771
MA0903.1	HOXB3	0.03	5511	15246	Down	4678874	3.59E-04	1557	4177	Down	0.006021	-0.22281	-0.04393	-2.22035
MA1138.1	FOSL2::JUNB	0.06	1135	2638	Up	4575141	0.036847	545	1257	Up	0.00611	-1.84564	0.174566	2.213977
MA0465.2	CDX2	0.15	4149	11924	Down	4695756	1.39E-04	1472	3970	Down	0.006183	-0.29995	-0.05158	-2.20877
MA0259.1	ARNT::HIF1A	0.02	2161	6184	Down	4614340	0.008119	1086	3001	Down	0.006341	-0.72106	-0.08652	-2.19786
MA0513.1	SMAD2::SMAD3::SMAD 4	0.14	2290	5242	Up	4667383	6.63E-04	1260	3111	Up	0.007265	-0.58795	0.075859	2.138752
MA0481.3	FOXP1	0.15	3728	10292	Down	4628005	0.004481	1488	4007	Down	0.007381	-0.28547	-0.04937	-2.1319
MA0873.1	HOXD12	0.15	1062	3111	Down	4617428	0.007121	780	2206	Down	0.007559	-1.18163	-0.11982	-2.12154
MA0786.1	POU3F1	0.15	2534	7051	Down	4626502	0.004792	1204	3299	Down	0.007654	-0.57841	-0.07434	-2.11611
MA1607.1	Foxl2	0.11	665	1910	Down	4576056	0.035684	529	1539	Down	0.007754	-1.72117	-0.16029	-2.1105
MA0132.2	PDX1	0.06	5347	14832	Down	4671084	5.46E-04	1536	4123	Down	0.007847	-0.24199	-0.04475	-2.10529
MA0675.1	NKX6-2	0.02	2013	5555	Down	4598038	0.015764	894	2500	Down	0.008533	-0.99306	-0.10359	-2.06891
MA0032.2	FOXC1	0.11	2358	6641	Down	4637983	0.00284	1132	3113	Down	0.008705	-0.66472	-0.07955	-2.06021
MA1499.1	HOXB4	0.08	5027	13730	Down	4641326	0.002427	1479	3982	Down	0.008976	-0.29436	-0.04909	-2.04692
MA1545.1	OVOL2	0.15	1318	3895	Down	4652336	0.001425	907	2533	Down	0.009209	-0.9732	-0.10169	-2.03577
MA0785.1	POU2F1	0.15	1991	5506	Down	4577135	0.034352	1066	2942	Down	0.009238	-0.74879	-0.08469	-2.03441
MA0661.1	MEOX1	0.12	4474	12297	Down	4624945	0.005135	1460	3934	Down	0.009526	-0.31243	-0.05024	-2.02107
MA0116.1	Znf423	0.12	1468	3408	Up	4603114	0.01289	802	1918	Up	0.009577	-1.26247	0.122068	2.018758
MA0004.1	Arnt	0.1	3047	8872	Down	4668825	6.15E-04	1304	3545	Down	0.009609	-0.46901	-0.06302	-2.01733
MA0704.1	Lhx4	0.1	8958	24673	Down	4708013	6.77E-05	1710	4533	Down	0.009713	-0.09623	-0.02673	-2.01264
MA1496.1	HOXA4	0.09	5328	14480	Down	4636938	0.002981	1523	4088	Down	0.01018	-0.25427	-0.04471	-1.99227
MA0707.1	MNX1	0.09	6185	16884	Down	4674799	4.48E-04	1562	4182	Down	0.010447	-0.21964	-0.04104	-1.98099
MA1649.1	ZBTB12	0.13	2929	8077	Down	4629828	0.004128	1432	3862	Down	0.010736	-0.33971	-0.05153	-1.96916
MA0909.2	HOXD13	0.15	1243	3448	Down	4579848	0.031189	851	2383	Down	0.010818	-1.06317	-0.10553	-1.96585
MA0813.1	TFAP2B(var.3)	0.13	1882	4661	Up	4568715	0.045966	976	2372	Up	0.010862	-0.9677	0.098757	1.964078
MA1123.2	TWIST1	0.04	645	1466	Up	4573459	0.039071	532	1236	Up	0.011046	-1.87519	0.164065	1.956789
MA0904.2	HOXB5	0.12	7468	20274	Down	4619547	0.006501	1577	4217	Down	0.01105	-0.20673	-0.03927	-1.95663
MA1511.1	KLF10	0.11	3007	7353	Up	4610389	0.009579	1330	3308	Up	0.011251	-0.50468	0.065265	1.948816
MA0717.1	RAX2	0.04	5630	15856	Down	4715696	4.24E-05	1534	4112	Down	0.011443	-0.24486	-0.04277	-1.94147

MA1608.1	Isl1	0.14	5126	14178	Down	4639770	0.002612	1642	4371	Down	0.011753	-0.15174	-0.03276	-1.92984
MA0493.1	Klf1	0.12	4329	10439	Up	4661196	9.13E-04	1530	3852	Up	0.011993	-0.29385	0.047682	1.921061
MA0027.2	EN1	0.1	8257	23356	Down	4778370	6.08E-07	1719	4550	Down	0.012848	-0.08974	-0.02456	-1.89117
MA0613.1	FOXG1	0.07	1829	4998	Down	4583927	0.026906	1069	2941	Down	0.013754	-0.747	-0.08014	-1.86156
MA0807.1	TBX5	0.15	13310	32524	Up	4708039	6.76E-05	1844	4781	Up	0.014082	-0.0034	0.00524	1.851325
MA0151.1	Arid3a	0.04	4272	12380	Down	4765060	1.60E-06	1545	4135	Down	0.014348	-0.23568	-0.04051	-1.84322
MA1560.1	SOHLH2	0.15	2016	5904	Down	4620176	0.006326	931	2583	Down	0.014634	-0.94027	-0.09223	-1.83463
MA1474.1	CREB3L4	0.15	1326	3928	Down	4618377	0.006837	874	2434	Down	0.015923	-1.02867	-0.09762	-1.79797
MA0630.1	SHOX	0.02	2828	7731	Down	4596966	0.016439	1098	3012	Down	0.01654	-0.7105	-0.07595	-1.78146
MA0649.1	HEY2	0.14	1729	5001	Down	4581090	0.029828	786	2203	Down	0.016605	-1.17709	-0.1068	-1.77976
MA0659.2	MAFG	0.1	974	2325	Up	4573104	0.039555	744	1784	Up	0.017809	-1.36882	0.118297	1.749368
MA0084.1	SRY	0.07	6547	18653	Down	4723681	2.58E-05	1672	4437	Down	0.018109	-0.12787	-0.02827	-1.7421
MA0160.1	NR4A2	0.04	959	2327	Up	4574636	0.037504	756	1814	Up	0.018145	-1.34526	0.117313	1.741248
MA0634.1	ALX3	0.04	2946	8144	Down	4610862	0.009393	1129	3089	Down	0.018224	-0.67222	-0.07222	-1.73935
MA0908.1	HOXD11	0.14	2331	6577	Down	4639795	0.002608	1248	3391	Down	0.018434	-0.53269	-0.06225	-1.73438
MA1647.1	PRDM4	0.07	1360	3280	Up	4595522	0.017387	956	2332	Up	0.018484	-0.99489	0.093433	1.733207
MA0158.2	HOXA5	0.08	3636	10098	Down	4651903	0.001456	1331	3600	Down	0.018883	-0.44312	-0.05567	-1.72394
MA1107.2	KLF9	0.15	9450	20454	Up	4695583	1.41E-04	1352	3381	Up	0.021514	-0.4771	0.057439	1.667271
MA1652.1	ZKSCAN5	0.14	2420	5738	Up	4648324	0.001735	1265	3148	Up	0.021964	-0.57656	0.064516	1.658288
MA0674.1	NKX6-1	0.1	6513	17764	Down	4673053	4.92E-04	1607	4278	Down	0.022633	-0.18279	-0.03281	-1.64526
MA0050.2	IRF1	0.15	1552	4329	Down	4573922	0.038448	934	2581	Down	0.022763	-0.93851	-0.08647	-1.64277
MA0823.1	HEY1	0.14	1877	5547	Down	4594887	0.01782	828	2301	Down	0.026262	-1.10817	-0.09453	-1.58068
MA0468.1	DUX4	0.15	1255	3599	Down	4591806	0.020053	848	2353	Down	0.02642	-1.07485	-0.09235	-1.57807
MA1628.1	Zic1::Zic2	0.01	1251	2875	Up	4609373	0.009991	864	2105	Up	0.02953	-1.1417	0.095248	1.529733
MA0892.1	GSX1	0.05	5773	15886	Down	4660596	9.42E-04	1603	4265	Down	0.029734	-0.18678	-0.03202	-1.52674
MA1501.1	HOXB7	0.15	3175	8622	Down	4581860	0.029008	1358	3658	Down	0.030387	-0.41712	-0.04976	-1.51731
MA1507.1	HOXD4	0.02	2856	7879	Down	4590468	0.021096	1136	3094	Down	0.030467	-0.6666	-0.06564	-1.51617
MA1504.1	HOXC4	0.03	2856	7879	Down	4590468	0.021096	1136	3094	Down	0.030467	-0.6666	-0.06564	-1.51617
MA1656.1	ZNF449	0.12	836	1931	Up	4571251	0.042163	639	1528	Up	0.0306	-1.59018	0.122373	1.514276
MA1498.1	HOXA7	0.07	7479	20435	Down	4649986	0.0016	1708	4514	Down	0.033151	-0.1001	-0.02236	-1.4795

MA0002.2	RUNX1	0.07	2735	6755	Up	4591992	0.019911	1413	3555	Up	0.034643	-0.40909	0.048693	1.460379
MA0890.1	GBX2	0.06	6062	17137	Down	4732134	1.50E-05	1584	4214	Down	0.036378	-0.20405	-0.03186	-1.43916
MA0155.1	INSM1	0.14	2989	7461	Up	4576711	0.034871	1394	3506	Up	0.037064	-0.42886	0.04919	1.431052
MA0654.1	ISX	0.05	2754	7452	Down	4576276	0.035409	1077	2937	Down	0.038233	-0.74261	-0.06743	-1.41756
MA0913.2	HOXD9	0.14	3926	10832	Down	4610595	0.009498	1541	4108	Down	0.038352	-0.24228	-0.0348	-1.41621
MA0087.1	Sox5	0.01	1551	4363	Down	4593172	0.019034	971	2663	Down	0.039065	-0.88794	-0.07556	-1.40821
MA1637.1	EBF3	0.15	1829	4317	Up	4584408	0.026436	940	2310	Up	0.039893	-1.0139	0.082768	1.399101
MA0868.2	SOX8	0.05	1550	4351	Down	4589870	0.021577	971	2661	Down	0.041771	-0.88849	-0.07448	-1.37913
MA1562.1	SOX14	0.15	1716	4786	Down	4586238	0.024712	1038	2833	Down	0.042657	-0.79521	-0.06861	-1.37001
MA0611.1	Dux	0.1	1598	4566	Down	4599852	0.014678	1032	2817	Down	0.042915	-0.80347	-0.0688	-1.36739
MA0077.1	SOX9	0.12	2877	7944	Down	4615773	0.007641	1364	3664	Down	0.044366	-0.41275	-0.04577	-1.35295
MA0616.2	HES2	0.12	1902	5574	Down	4586139	0.024803	858	2365	Down	0.045453	-1.06273	-0.08278	-1.34244
MA0755.1	CUX2	0.14	2792	7770	Down	4610782	0.009424	1281	3453	Down	0.045862	-0.5008	-0.05075	-1.33855
MA0764.2	ETV4	0.02	5666	13798	Up	4667559	6.57E-04	1713	4384	Up	0.046455	-0.11907	0.024007	1.33297
MA1530.1	NKX6-3	0.12	5890	16343	Down	4677639	3.84E-04	1678	4438	Down	0.048547	-0.12513	-0.02343	-1.31384
MA0640.2	ELF3	0.13	5029	12208	Up	4664100	7.87E-04	1683	4302	Up	0.049173	-0.14543	0.025762	1.308274
MA0876.1	BSX	0.05	8773	24250	Down	4706525	7.40E-05	1745	4596	Down	0.049428	-0.07166	-0.01742	-1.30602

Supplementary Table S9. Summary of over- and under-represented DNA binding sites for transcription factors (TFs) in genes with high variability (p values <0.01) vs. low variability (p>0.7) found in whole brain of stressed zebrafish vs. control groups using CiiiDer TFMs software [S4] (Experiment 1). Only TFs with p<0.05 simultaneously for gene coverage p-value and the number of the binding sites distributions were considered significant. TFs in the table are listed as sorted by their gene coverage p-value.

TF ID	TF Name	Deficit	N sites	N background sites	Direction	MW U	P-Value	N TF (of 1964)	N TF background genes (of 3948)	Direction	P-Value	Average Log2 Proportion Bound	Log2 Enrichment	Significance Score
MA0885.1	Dlx2	0	443	1011	Down	12586771	3.56E-12	415	885	Down	1.3E-31	-2.99159	-0.90905	-30.8849
MA0051.1	IRF2	0.09	416	293	Up	11986070	0.010281	381	278	Up	5.22E-09	-3.88758	0.6366	8.282529
MA1125.1	ZNF384	0	281	494	Down	11997843	0.007996	266	462	Down	6.22E-09	-3.78046	-0.61272	-8.20643
MA0708.1	MSX2	0.01	3814	4894	Down	12335139	3.13E-07	2484	3067	Down	1.74E-06	-0.80532	-0.12151	-5.76017
MA0517.1	STAT1::STAT2	0.1	1030	909	Up	12056560	0.002063	880	821	Up	8.8E-06	-2.50397	0.282657	5.05553
MA0632.2	TCFL5	0.1	2842	3772	Down	12093561	0.000804	1284	1651	Down	6.01E-05	-1.72784	-0.17998	-4.22101
MA0156.2	FEV	0.15	4631	5757	Down	12159601	0.000126	2647	3206	Down	8.13E-05	-0.72752	-0.09377	-4.09015
MA0645.1	ETV6	0.04	474	695	Down	11913655	0.041613	431	611	Down	0.000178	-3.2314	-0.3204	-3.74992
MA1122.1	TFDP1	0.15	1696	2148	Down	12028351	0.00404	1246	1590	Down	0.000241	-1.77665	-0.169	-3.6178
MA1099.2	HES1	0.15	13065	16225	Down	12161049	0.000121	3162	3760	Down	0.000299	-0.48434	-0.06726	-3.525
MA0594.2	HOXA9	0.07	6774	8198	Down	12176525	7.58E-05	3319	3931	Down	0.000316	-0.41731	-0.06152	-3.49989
MA0748.2	YY2	0.07	532	748	Down	11914896	0.040714	489	678	Down	0.000319	-3.06543	-0.28845	-3.49559
MA0765.2	ETV5	0.01	488	708	Down	11905589	0.047877	445	623	Down	0.000346	-3.19435	-0.30237	-3.46132
MA0474.2	ERG	0.09	1017	1327	Down	11972549	0.013606	847	1112	Down	0.00035	-2.31279	-0.20993	-3.45626
MA1651.1	ZFP42	0.11	670	932	Down	11942789	0.02444	614	830	Down	0.00039	-2.75556	-0.25197	-3.40915
MA0092.1	Hand1::Tcf3	0.05	4913	5183	Up	12060545	0.00187	2899	3111	Up	0.000448	-0.68362	0.080789	3.348324
MA0763.1	ETV3	0.13	1064	1408	Down	11975009	0.012938	906	1176	Down	0.000622	-2.22389	-0.19353	-3.20625
MA0076.2	ELK4	0.09	779	1037	Down	11930027	0.03101	665	886	Down	0.000636	-2.65098	-0.23109	-3.19647
MA0909.2	HOXD13	0.13	5247	6185	Down	11952926	0.020116	2981	3546	Down	0.00094	-0.56911	-0.06776	-3.02696
MA0509.2	RFX1	0.15	2354	2666	Up	12031302	0.003772	1570	1948	Down	0.001025	-1.46354	-0.12855	-2.98915
MA1484.1	ETS2	0.11	936	1229	Down	11941775	0.024913	796	1038	Down	0.001055	-2.40721	-0.20016	-2.97656
MA0098.3	ETS1	0.09	709	945	Down	11907089	0.046655	618	821	Down	0.001341	-2.75874	-0.22689	-2.8725
MA0615.1	Gmeb1	0.12	29950	36083	Down	12244753	8.37E-06	4215	4865	Down	0.001354	-0.09118	-0.02429	-2.86829
MA0471.2	E2F6	0.13	1435	1822	Down	11971227	0.013977	1152	1456	Down	0.001415	-1.89669	-0.15514	-2.84929

MA0524.2	TFAP2C	0.14	2277	2843	Down	11993241	0.008829	1479	1837	Down	0.001486	-1.5489	-0.13004	-2.82794
MA0798.2	RFX3	0.15	1151	1196	Up	11903372	0.049732	712	933	Down	0.001508	-2.56449	-0.20717	-2.82166
MA0759.1	ELK3	0.14	2712	3374	Down	12056331	0.002074	1860	2274	Down	0.00174	-1.22971	-0.10726	-2.75935
MA1545.1	OVOL2	0.14	2143	2583	Down	11962186	0.016765	1594	1968	Down	0.001766	-1.44523	-0.1214	-2.75302
MA0259.1	ARNT::HIF1A	0.09	14715	17251	Down	11984815	0.010555	4074	4718	Down	0.001768	-0.13785	-0.02911	-2.75241
MA0619.1	LIN54	0.02	13952	16575	Down	12098117	0.000713	4052	4694	Down	0.002003	-0.14544	-0.02956	-2.69839
MA0877.2	BARHL1	0.07	8044	9742	Down	12122330	0.000369	3550	4159	Down	0.002149	-0.32811	-0.04579	-2.66786
MA0679.2	ONECUT1	0.09	3504	3642	Up	12012150	0.005838	2208	2345	Up	0.002194	-1.08385	0.095766	2.658862
MA0651.1	HOXC11	0.12	3549	4325	Down	12030797	0.003817	2344	2820	Down	0.002333	-0.90772	-0.08408	-2.6321
MA0818.1	BHLHE22	0.08	3638	3709	Up	11974059	0.013192	1508	1562	Up	0.00234	-1.65185	0.131853	2.630717
MA0898.1	Hmx3	0.09	1296	1584	Down	11932632	0.029558	1053	1332	Down	0.002478	-2.02567	-0.15635	-2.60592
MA0823.1	HEY1	0.15	4805	5804	Down	11965101	0.015817	2239	2700	Down	0.002549	-0.97213	-0.08746	-2.59368
MA0811.1	TFAP2B	0.12	1795	2247	Down	11961279	0.01707	1246	1558	Down	0.002615	-1.79131	-0.13968	-2.58256
MA1554.1	RFX7	0.1	10098	11682	Down	12032098	0.003703	3877	4508	Down	0.002647	-0.20644	-0.03493	-2.57725
MA0614.1	Foxj2	0.02	5600	6611	Down	11978672	0.011998	3052	3608	Down	0.002962	-0.53964	-0.05881	-2.52835
MA0157.2	FOXO3	0.13	5600	6611	Down	11978672	0.011998	3052	3608	Down	0.002962	-0.53964	-0.05881	-2.52835
MA0678.1	OLIG2	0.09	3110	3210	Up	11930301	0.030855	1413	1461	Up	0.00302	-1.74697	0.134417	2.520008
MA0668.1	NEUROD2	0.15	8156	8739	Up	11963420	0.016358	2918	3162	Up	0.003143	-0.66718	0.066756	2.502623
MA0862.1	GMEB2	0.13	16172	19559	Down	12112995	0.000477	3798	4421	Down	0.003261	-0.23535	-0.03651	-2.48665
MA0827.1	OLIG3	0.08	3201	3317	Up	11931979	0.029916	1526	1589	Up	0.003724	-1.63093	0.124249	2.428977
MA0613.1	FOXG1	0.03	7201	8485	Down	12000594	0.007533	3445	4036	Down	0.004047	-0.37142	-0.0458	-2.39291
MA0502.2	NFYB	0.1	1480	1556	Up	11920082	0.037132	1236	1271	Up	0.004233	-1.94393	0.142326	2.373348
MA0762.1	ETV2	0.14	952	1239	Down	11912644	0.042358	832	1063	Down	0.004377	-2.35816	-0.1707	-2.35879
MA1468.1	ATOH7	0.11	7111	7525	Up	12045370	0.002706	2906	3154	Up	0.00445	-0.67198	0.064466	2.351649
MA0604.1	Atf1	0.07	5000	5949	Down	12005434	0.006776	2688	3196	Down	0.004518	-0.71868	-0.0671	-2.3451
MA0826.1	OLIG1	0.13	3467	3552	Up	11943295	0.024206	1459	1519	Up	0.004724	-1.6958	0.124472	2.32565
MA0616.2	HES2	0.15	4682	5643	Down	11944748	0.023546	2091	2521	Down	0.004866	-1.07092	-0.08715	-2.31279
MA0475.2	FLI1	0.15	2124	2627	Down	11976605	0.012521	1540	1889	Down	0.004928	-1.49963	-0.11201	-2.30734
MA0686.1	SPDEF	0.15	1004	1310	Down	11905049	0.048323	825	1053	Down	0.004986	-2.37107	-0.16926	-2.30221
MA0817.1	BHLHE23	0.12	3380	3466	Up	11934714	0.028439	1436	1495	Up	0.005213	-1.71874	0.124525	2.282877

MA0146.2	Zfx	0.12	3429	4030	Down	11940580	0.025482	2066	2491	Down	0.005491	-1.08823	-0.08723	-2.26033
MA0483.1	Gfi1b	0.08	1316	1653	Down	11907688	0.046175	1133	1412	Down	0.006326	-1.93081	-0.13487	-2.1989
MA0499.2	MYOD1	0.13	8050	8826	Up	12018468	0.005065	3647	4026	Up	0.007598	-0.33211	0.039976	2.119277
MA0884.1	DUXA	0.06	2082	2524	Down	11939719	0.025899	1611	1963	Down	0.007819	-1.43941	-0.10243	-2.10687
MA0520.1	Stat6	0.15	6243	6683	Up	11960997	0.017166	2333	2511	Up	0.009089	-0.99481	0.076541	2.041493
MA1579.1	ZBTB26	0.04	2358	2451	Up	11945720	0.023113	1750	1855	Up	0.010134	-1.42054	0.098554	1.994212
MA1647.1	PRDM4	0.08	5231	5702	Up	11910933	0.043645	3068	3356	Up	0.010878	-0.58809	0.053171	1.963459
MA0913.2	HOXD9	0.05	1618	1995	Down	11907816	0.046073	1323	1624	Down	0.011606	-1.71815	-0.11304	-1.93533
MA0658.1	LHX6	0.02	3052	3721	Down	11920011	0.037179	1378	1687	Down	0.011786	-1.66134	-0.10919	-1.92865
MA0144.2	STAT3	0.09	1713	1792	Up	11904540	0.048747	1337	1398	Up	0.012015	-1.81862	0.118253	1.920258
MA1570.1	TFAP4(var.2)	0.04	5368	5773	Up	11923053	0.035204	2220	2388	Up	0.012088	-1.06684	0.077374	1.917659
MA0911.1	Hoxa11	0.12	5368	6290	Down	11985744	0.010351	2987	3513	Down	0.012531	-0.57441	-0.05138	-1.90201
MA0846.1	FOXC2	0.07	4744	5444	Down	11909213	0.044971	2746	3243	Down	0.013253	-0.69276	-0.05736	-1.8777
MA1568.1	TCF21(var.2)	0.12	3968	4137	Up	11920347	0.036957	1707	1814	Up	0.014872	-1.4546	0.094908	1.827642
MA1542.1	OSR1	0.13	2422	2895	Down	11908991	0.045145	1781	2146	Down	0.016077	-1.30279	-0.0863	-1.79379
MA0607.1	Bhlha15	0.1	8827	9413	Up	12040871	0.003012	3045	3337	Up	0.016929	-0.59761	0.050506	1.771379
MA1153.1	Smad4	0.03	2684	2828	Up	11915745	0.040108	1922	2059	Up	0.018245	-1.27769	0.083284	1.738868
MA1508.1	IKZF1	0.09	3898	4176	Up	11948267	0.022012	2564	2789	Up	0.020531	-0.85099	0.061266	1.687591
MA0623.2	NEUROG1	0.08	4892	5165	Up	11907567	0.046272	1846	1977	Up	0.021863	-1.3361	0.08371	1.660299
MA0508.3	PRDM1	0.11	2394	2519	Up	11909715	0.04458	1776	1899	Up	0.022125	-1.393	0.086013	1.655108
MA1606.1	Foxf1	0.02	7872	9260	Down	11962227	0.016752	3579	4158	Down	0.02325	-0.32242	-0.03371	-1.63357
MA0635.1	BARHL2	0.1	41687	48335	Down	12056922	0.002044	4526	5128	Up	0.02575	-0.00186	0.002454	1.589221
MA0800.1	EOMES	0.13	5066	5529	Up	11917174	0.039106	3010	3306	Up	0.02742	-0.61268	0.047293	1.561926
MA0601.1	Arid3b	0.02	5494	6689	Down	11944901	0.023478	2640	3109	Down	0.029286	-0.75158	-0.05328	-1.53334
MA0148.4	FOXA1	0.11	5155	6105	Down	11974997	0.012941	2964	3464	Down	0.044643	-0.59011	-0.04226	-1.35024
MA0899.1	HOXA10	0.05	8936	10518	Down	11972711	0.013561	3701	4279	Down	0.049974	-0.27755	-0.02674	-1.30126

Supplementary Table S10. Statistical data for pathways enriched in combined with 1:1:1 orthologue map mdd and stress samples in combined human (subiculum), rat (hippocampus) and zebrafish (whole brain) data vs. combined control groups (Experiment 2). The KEGG- enrichment analysis was performed on normalized and log2-transformed counts by general applicable gene set enrichment for pathway analysis (GAGE) package [S4], using two-tailed t-test for group comparison of differential expression of gene sets. False discovery rate cutoff (q.val) was set at 0.05. p.geomean – geometric mean of the individual p-values from multiple single array based gene set tests, stat.mean - mean of the individual statistics from multiple single array based gene set tests. Pathways are listed as sorted by q value.

	**p.geomean**	**stat.mean**	**p.val**	**q.val**	**set.size**
Bidirected					
hsa04080 Neuroactive ligand-receptor interaction	6.43E-05	3.747224	7.93E-38	2.36E-35	199
hsa04020 Calcium signaling pathway	0.002036	2.756785	1.47E-21	2.18E-19	175
hsa04512 ECM-receptor interaction	0.0103	2.291673	2.86E-15	2.83E-13	60
hsa05414 Dilated cardiomyopathy	0.011529	2.175357	5.66E-14	4.21E-12	73
hsa04024 cAMP signaling pathway	0.014065	2.128332	1.14E-13	6.8E-12	164
hsa05410 Hypertrophic cardiomyopathy (HCM)	0.018104	2.042219	1.44E-12	7.13E-11	69
hsa04742 Taste transduction	0.031838	1.852065	1.53E-10	6.51E-09	37
hsa05033 Nicotine addiction	0.029919	1.83466	2.93E-10	1.09E-08	31
hsa05032 Morphine addiction	0.020663	1.793306	5.03E-10	1.66E-08	73
hsa04510 Focal adhesion	0.035082	1.751335	7.62E-10	2.26E-08	156
hsa04360 Axon guidance	0.028882	1.727033	1.36E-09	3.68E-08	145
hsa04724 Glutamatergic synapse	0.033114	1.689927	3.48E-09	8.61E-08	85
hsa04010 MAPK signaling pathway	0.035732	1.671304	3.96E-09	9.05E-08	223
hsa04725 Cholinergic synapse	0.037452	1.661053	5.86E-09	1.24E-07	92
hsa05412 Arrhythmogenic right ventricular cardiomyopathy (ARVC)	0.047739	1.648319	7.74E-09	1.49E-07	59
hsa04261 Adrenergic signaling in cardiomyocytes	0.033242	1.643525	8.01E-09	1.49E-07	110
hsa05200 Pathways in cancer	0.038035	1.626444	9.4E-09	1.64E-07	374
hsa04151 PI3K-Akt signaling pathway	0.042373	1.594397	1.83E-08	2.96E-07	242
hsa04022 cGMP-PKG signaling pathway	0.043272	1.59675	1.9E-08	2.96E-07	118
hsa04713 Circadian entrainment	0.055321	1.511983	1.06E-07	1.57E-06	69
hsa04974 Protein digestion and absorption	0.054494	1.493315	1.56E-07	2.21E-06	63
hsa04925 Aldosterone synthesis and secretion	0.050952	1.484697	1.74E-07	2.35E-06	75
hsa04726 Serotonergic synapse	0.066804	1.471338	2.08E-07	2.69E-06	76
hsa05146 Amoebiasis	0.07315	1.429375	4.49E-07	5.55E-06	59
hsa04380 Osteoclast differentiation	0.076023	1.344395	1.86E-06	2.21E-05	83
hsa05030 Cocaine addiction	0.087137	1.316132	3.41E-06	3.89E-05	38
hsa04668 TNF signaling pathway	0.085639	1.305408	3.54E-06	3.89E-05	71
hsa04060 Cytokine-cytokine receptor interaction	0.070836	1.302955	3.82E-06	4.05E-05	83
hsa04610 Complement and coagulation cascades	0.052226	1.321515	4.09E-06	4.19E-05	46
hsa04727 GABAergic synapse	0.075184	1.29523	4.42E-06	4.38E-05	70
hsa05031 Amphetamine addiction	0.088336	1.26116	7.87E-06	7.54E-05	46
hsa04923 Regulation of lipolysis in adipocytes	0.093031	1.24333	1.04E-05	9.63E-05	45
hsa04015 Rap1 signaling pathway	0.105125	1.206869	1.52E-05	0.000135	162

hsa04728 Dopaminergic synapse	0.088897	1.207947	1.61E-05	0.000135	95
hsa04918 Thyroid hormone synthesis	0.107011	1.210822	1.62E-05	0.000135	54
hsa04933 AGE-RAGE signaling pathway in diabetic complications	0.098533	1.206712	1.64E-05	0.000135	71
hsa04913 Ovarian steroidogenesis	0.090988	1.208348	1.9E-05	0.000153	39
hsa05205 Proteoglycans in cancer	0.076901	1.19177	1.99E-05	0.000156	162
hsa04912 GnRH signaling pathway	0.078631	1.197353	2.05E-05	0.000156	65
hsa04921 Oxytocin signaling pathway	0.097233	1.18653	2.14E-05	0.000159	107
hsa04917 Prolactin signaling pathway	0.093764	1.185497	2.42E-05	0.000166	58
hsa04640 Hematopoietic cell lineage	0.092253	1.202767	2.43E-05	0.000166	23
hsa04924 Renin secretion	0.108886	1.183691	2.44E-05	0.000166	44
hsa04514 Cell adhesion molecules (CAMs)	0.113258	1.178033	2.46E-05	0.000166	72
hsa04976 Bile secretion	0.120613	1.162656	3.16E-05	0.000209	50
hsa05133 Pertussis	0.089438	1.144918	5.01E-05	0.000323	43
hsa04740 Olfactory transduction	0.11329	1.103163	9.38E-05	0.000593	25
hsa04390 Hippo signaling pathway	0.107835	1.054247	0.00014	0.00085	120
hsa05150 Staphylococcus aureus infection	0.103762	1.087561	0.00014	0.00085	22
hsa05144 Malaria	0.125834	1.062982	0.000155	0.000919	23
hsa04723 Retrograde endocannabinoid signaling	0.097371	1.043756	0.000165	0.000963	120
hsa04916 Melanogenesis	0.100434	1.044096	0.000173	0.000986	81
hsa04911 Insulin secretion	0.110966	1.013207	0.000253	0.001419	58
hsa04971 Gastric acid secretion	0.111337	0.99542	0.000328	0.001805	50
hsa04270 Vascular smooth muscle contraction	0.140633	0.979952	0.000368	0.001987	78
hsa04750 Inflammatory mediator regulation of TRP channels	0.143622	0.979324	0.000376	0.001992	62
hsa05418 Fluid shear stress and atherosclerosis	0.146413	0.965169	0.000434	0.002261	96
hsa04371 Apelin signaling pathway	0.159676	0.936328	0.000615	0.003149	100
hsa05320 Autoimmune thyroid disease	0.158784	0.982107	0.000647	0.003255	10
hsa04014 Ras signaling pathway	0.152909	0.928409	0.000668	0.003306	164
hsa04970 Salivary secretion	0.122053	0.923143	0.000801	0.003901	51
hsa04659 Th17 cell differentiation	0.150241	0.909645	0.000879	0.004208	60
hsa04260 Cardiac muscle contraction	0.14605	0.908278	0.000912	0.004298	56
hsa04550 Signaling pathways regulating pluripotency of stem cells	0.146366	0.901617	0.000938	0.004351	111
hsa05142 Chagas disease (American trypanosomiasis)	0.167786	0.896403	0.001001	0.004573	70
hsa04744 Phototransduction	0.085489	1.005784	0.001093	0.004917	19
hsa04540 Gap junction	0.172421	0.855701	0.001593	0.007061	70
hsa04310 Wnt signaling pathway	0.123629	0.834726	0.002038	0.008901	122
hsa00830 Retinol metabolism	0.17417	0.824523	0.002588	0.011141	25
hsa05204 Chemical carcinogenesis	0.185074	0.807223	0.003039	0.012893	20
hsa05202 Transcriptional misregulation in cancer	0.189504	0.790851	0.003151	0.012907	119
hsa04930 Type II diabetes mellitus	0.136246	0.802107	0.003158	0.012907	40
hsa04062 Chemokine signaling pathway	0.17057	0.791513	0.003172	0.012907	115
hsa02010 ABC transporters	0.199764	0.77908	0.003772	0.015139	30
hsa04658 Th1 and Th2 cell differentiation	0.173363	0.766497	0.004222	0.016718	50
hsa04611 Platelet activation	0.21025	0.750968	0.004752	0.018569	90
hsa04721 Synaptic vesicle cycle	0.174376	0.751912	0.004965	0.019151	54
hsa05416 Viral myocarditis	0.206648	0.751062	0.005139	0.019569	24
hsa04660 T cell receptor signaling pathway	0.220491	0.741125	0.005251	0.019743	73

hsa00910 Nitrogen metabolism	0.207374	0.741918	0.006424	0.023848	12
hsa05321 Inflammatory bowel disease (IBD)	0.194381	0.69712	0.008996	0.03274	23
hsa05222 Small cell lung cancer	0.225411	0.685255	0.009039	0.03274	72
hsa05224 Breast cancer	0.202341	0.68059	0.009421	0.033711	118
hsa05140 Leishmaniasis	0.203921	0.668531	0.010979	0.038817	42
hsa04915 Estrogen signaling pathway	0.210616	0.645201	0.013004	0.045357	83
hsa05161 Hepatitis B	0.221626	0.642686	0.013202	0.045357	118
hsa05217 Basal cell carcinoma	0.162746	0.649796	0.013348	0.045357	55
hsa05166 HTLV-I infection	0.242782	0.639881	0.013439	0.045357	158
Upregulated					
hsa00071 Fatty acid degradation	0.07651	1.073767	0.000142	0.023071	32
hsa00010 Glycolysis / Gluconeogenesis	0.071185	1.067887	0.000155	0.023071	45
Downregulated					
hsa05032 Morphine addiction	0.041747	-1.06461	0.000138	0.041101	73

Supplementary Table S11. Summary of statistical analyses for all differentially expressed genes in subiculum of mdd vs. control groups using only orthologues 1:1:1 mapped between human, rat and zebrafish (Experiment 3), on the Negative Binomial (Gamma-Poisson) distribution performed by estimation of size factors, estimation of dispersion and negative binomial generalized linear models and Wald statistics using the DESeq function [S3]. P value and false discovery rate were set at 0.05. Genes in this table are listed as sorted by their p-adjusted value.

	baseMean	log2FoldChange	lfcSE	stat	pvalue	padj
TRHDE	2464.401	-1.33853	0.250472	-5.34405	9.09E-08	0.000864
VWF	838.6989	0.857176	0.171901	4.986457	6.15E-07	0.002924
APBA2	137.3063	-2.26438	0.479413	-4.72323	2.32E-06	0.005564
ELAVL2	4350.698	-1.01495	0.214962	-4.72156	2.34E-06	0.005564
NEUROD6	4729.525	-1.16977	0.269735	-4.33673	1.45E-05	0.023136
LENG1	16.44758	-5.44315	1.255718	-4.33469	1.46E-05	0.023136
STMN2	23897.14	-0.63013	0.151397	-4.16208	3.15E-05	0.030895
FAM102B	4352.728	-0.62967	0.149692	-4.20645	2.59E-05	0.030895
KIF19	87.06534	1.546379	0.372146	4.155296	3.25E-05	0.030895
PTPRB	2267.339	0.656183	0.157768	4.159176	3.19E-05	0.030895
FABP3	3154.604	-0.86153	0.210791	-4.08714	4.37E-05	0.035749
SUCLG2	725.5435	0.648311	0.159644	4.060975	4.89E-05	0.035749
SLC39A10	8477.13	-0.54222	0.133074	-4.07455	4.61E-05	0.035749
PARP9	704.5983	0.763285	0.190791	4.00064	6.32E-05	0.042911
IFI44	625.3891	0.783843	0.197873	3.961346	7.45E-05	0.047251

Supplementary Table S12. Summary of statistical analyses for all differentially expressed genes in hippocampus of stressed rats vs. control groups using only orthologues 1:1:1 mapped between human, rat and zebrafish (Experiment 3), on the Negative Binomial (Gamma-Poisson) distribution performed by estimation of size factors, estimation of dispersion and negative binomial generalized linear models and Wald statistics using the DESeq function [S3]. P value and false discovery rate were set at 0.05. Genes in this table are listed as sorted by their p-adjusted value.

	baseMean	log2FoldChange	lfcSE	stat	pvalue	padj
HGF	404.1687	1.895295	0.27399	6.917389	4.6E-12	4.56E-08
FEZF2	894.0668	1.017009	0.160627	6.331481	2.43E-10	1.2E-06
TTC22	397.1618	1.666487	0.311176	5.355447	8.53E-08	0.000282
TP53BP2	5699.253	1.219034	0.234365	5.201431	1.98E-07	0.00049
KCNS1	297.6239	1.432952	0.303183	4.726361	2.29E-06	0.004529
OVOL2	49.41324	4.594552	0.988002	4.650346	3.31E-06	0.005471
HMGCS1	19775.1	0.769573	0.169208	4.548085	5.41E-06	0.007661
PPL	553.8464	-3.68711	0.820857	-4.49179	7.06E-06	0.008745
SLC5A7	297.6599	-2.70201	0.605174	-4.46485	8.01E-06	0.008819
HDC	397.5538	-4.0302	0.913243	-4.41306	1.02E-05	0.010096
USH2A	53.559	-1.8748	0.434595	-4.3139	1.6E-05	0.012222
RSPH10B	857.4857	-1.7044	0.393796	-4.32814	1.5E-05	0.012222
RSPH10B2	857.4857	-1.7044	0.393796	-4.32814	1.5E-05	0.012222
LDLR	1373.883	-0.75762	0.176533	-4.29167	1.77E-05	0.012548
RPE65	171.3564	-1.46829	0.35072	-4.1865	2.83E-05	0.018545
PIK3C2B	910.9034	-0.81143	0.19441	-4.17382	3E-05	0.018545
NADK2	6227.18	0.559755	0.134872	4.15028	3.32E-05	0.01935
FCHO2	3209.427	0.641262	0.155073	4.135236	3.55E-05	0.019514
TCEA2	6189.194	0.658331	0.161734	4.070461	4.69E-05	0.024463
ROBO3	784.5646	-3.0085	0.745855	-4.03363	5.49E-05	0.025907
VAV3	1312.649	-1.86314	0.461333	-4.0386	5.38E-05	0.025907
THRB	2458.737	1.334263	0.334114	3.993437	6.51E-05	0.028515
FAM101B	162.4399	-1.12712	0.282521	-3.98952	6.62E-05	0.028515
CPLX3	292.824	2.440444	0.61704	3.955083	7.65E-05	0.02915
SLC7A11	4068.756	0.536608	0.135153	3.970372	7.18E-05	0.02915
IGFN1	64.61423	2.076292	0.524425	3.959179	7.52E-05	0.02915
VANGL2	360.2331	-1.15621	0.304274	-3.79989	0.000145	0.049447
PHACTR3	10433.5	0.688542	0.180439	3.815924	0.000136	0.049447
MFRP	138.3103	-3.23802	0.851087	-3.80457	0.000142	0.049447

Supplementary Table S13. Summary of statistical analyses for all differentially expressed genes in whole brain of stressed zebrafish vs. control groups using only orthologues 1:1:1 mapped between human, rat and zebrafish (Experiment 3), on the Negative Binomial (Gamma-Poisson) distribution performed by estimation of size factors, estimation of dispersion and negative binomial generalized linear models and Wald statistics using the DESeq function [S3]. P value and false discovery rate were set at 0.05. Genes in this table are listed as sorted by their p-adjusted value.

	baseMean	log2FoldChange	lfcSE	stat	pvalue	padj
CBS	1373.633	-1.12835	0.164931	-6.84132	7.85E-12	4.03E-08
LOC102724560	1373.633	-1.12835	0.164931	-6.84132	7.85E-12	4.03E-08
TAPBP	164.2932	-1.79661	0.317186	-5.66421	1.48E-08	5.06E-05
SOCS3	314.5642	-2.29253	0.435013	-5.27004	1.36E-07	0.000351
CCR9	56.59636	-2.79566	0.554845	-5.03863	4.69E-07	0.000964
MYH4	258.5984	3.323343	0.709557	4.683685	2.82E-06	0.004829
THEMIS	17.14337	-1.83225	0.397957	-4.60414	4.14E-06	0.006085
TTF2	178.6828	-0.68797	0.150573	-4.56902	4.9E-06	0.006299
STAT4	36.67781	-1.59845	0.364297	-4.38778	1.15E-05	0.010946
MYH6	19.15213	-5.21277	1.187409	-4.39004	1.13E-05	0.010946
HMHA1	126.6126	-1.07557	0.245398	-4.38296	1.17E-05	0.010946
IQCE	417.4338	0.839114	0.194836	4.306767	1.66E-05	0.014117
MARCO	219.1724	-2.25995	0.528794	-4.27378	1.92E-05	0.014117
TBC1D10C	46.85412	-1.21312	0.283688	-4.27626	1.9E-05	0.014117
GNGT1	156.3481	-4.61752	1.093479	-4.22278	2.41E-05	0.01551
GMIP	52.35498	-1.07836	0.254683	-4.23413	2.29E-05	0.01551
ELF1	172.8876	-0.74862	0.179926	-4.16072	3.17E-05	0.017064
TAGAP	108.7985	-1.68608	0.404148	-4.17193	3.02E-05	0.017064
C4A	226.9903	-2.21315	0.533233	-4.15043	3.32E-05	0.017064
C4B	226.9903	-2.21315	0.533233	-4.15043	3.32E-05	0.017064
AP1M1	53.98701	-1.23875	0.301151	-4.1134	3.9E-05	0.019093
CEBPD	330.8055	-2.64475	0.646564	-4.09048	4.3E-05	0.019249
CAPG	55.92728	-1.16564	0.284259	-4.10063	4.12E-05	0.019249
SRR	22.26485	1.843807	0.453925	4.061918	4.87E-05	0.020856
C1orf158	34.69932	1.533384	0.378823	4.047754	5.17E-05	0.021272
SOCS1	66.27568	-1.74214	0.434759	-4.00715	6.15E-05	0.023338
LRIT1	29.5476	-1.49938	0.375628	-3.99165	6.56E-05	0.023338
GRN	462.8873	-0.9894	0.246331	-4.01654	5.91E-05	0.023338
BCL10	18.78615	-1.46481	0.367033	-3.99095	6.58E-05	0.023338
RDH5	27.74743	-3.85398	0.973009	-3.96089	7.47E-05	0.023997
RPAP1	260.3229	-0.6462	0.162521	-3.9761	7.01E-05	0.023997
LTB4R	36.54535	-1.56635	0.394873	-3.96671	7.29E-05	0.023997
LCK	21.17211	-1.98351	0.503234	-3.94152	8.1E-05	0.025233
JAKMIP3	1017.44	0.881741	0.225268	3.91419	9.07E-05	0.027437
TMEM129	265.5617	0.490474	0.12581	3.898534	9.68E-05	0.028436
GPR183	21.22254	-1.58352	0.407132	-3.88944	0.0001	0.028702
IL21R	67.83947	-1.30991	0.337564	-3.88048	0.000104	0.028976

INPP5D	120.971	-1.13115	0.294056	-3.84672	0.00012	0.032397
NPM1	438.6385	-0.43264	0.114254	-3.78662	0.000153	0.035353
POLR3E	636.9908	0.723905	0.191083	3.788444	0.000152	0.035353
CDH17	66.11075	4.632365	1.226162	3.777938	0.000158	0.035353
PTPRC	384.383	-1.11542	0.293817	-3.79631	0.000147	0.035353
JAK3	54.23865	-1.23074	0.322407	-3.81736	0.000135	0.035353
INPP5K	46.40485	-2.27796	0.602304	-3.78207	0.000156	0.035353
CNPY2	149.4071	0.724139	0.191647	3.778497	0.000158	0.035353
SAG	41.19081	-4.07355	1.072016	-3.7999	0.000145	0.035353
MTMR1	304.7362	0.633281	0.168459	3.759255	0.00017	0.037289
ZAP70	36.76595	-1.43135	0.381875	-3.74821	0.000178	0.03738
TNIP2	13.41921	-1.84403	0.49148	-3.752	0.000175	0.03738
FOSL1	61.16041	-1.45478	0.391125	-3.71947	0.0002	0.039933
ROR1	41.99598	-1.01654	0.273514	-3.71661	0.000202	0.039933
DHX58	85.19354	-3.31629	0.892052	-3.71759	0.000201	0.039933
CYP1A1	26.7201	3.443234	0.932283	3.693337	0.000221	0.042194
TPD52L2	1790.857	0.378175	0.102645	3.684304	0.000229	0.042194
MAD1L1	138.1593	-0.60458	0.164116	-3.68383	0.00023	0.042194
CRH	571.7543	0.593343	0.160978	3.685869	0.000228	0.042194
NPTX1	5347.391	0.368046	0.100279	3.670221	0.000242	0.043723
CPEB1	55.62308	1.039557	0.285336	3.643278	0.000269	0.046921
DNAJB1	27.47223	-0.87949	0.24121	-3.64614	0.000266	0.046921
PPP1R1C	919.5042	0.932417	0.257008	3.627964	0.000286	0.04816
XAF1	104.8072	-2.12535	0.585671	-3.62892	0.000285	0.04816
SLA	13.72092	-1.86999	0.516322	-3.62176	0.000293	0.048535

Supplementary Table S14. Summary of statistical analyses of Fisher meta-analysis between genes analyzed using DESeq function of human (Supplementary Table S11), rat (Supplementary Table S12) and zebrafish (Supplementary Table S13) vs. control groups using only orthologues 1:1:1 mapped between human, rat and zebrafish (Experiment 3) using the metaRNASeq R package [S5]. P value and false discovery rate were set at 0.05. Genes in this table are listed as sorted by their combined p-adjusted value and further compared using similarity in log 2-fold change direction between all species.

**Gene name**	**fishcomb.TestStatistic**	**fishcomb.rawpval**	**fishcomb.adjpval**	**DE in any study**	**Same l2fc direction**
CBS	59.74788832	5.06432E-11	1.74753E-07	TRUE	-
LOC102724560	60.83906421	3.0393E-11	1.74753E-07	TRUE	-
HGF	59.87689827	4.7678E-11	1.74753E-07	TRUE	-
TP53BP2	48.8161146	8.11522E-09	2.10022E-05	TRUE	-
FEZF2	48.13837725	1.10873E-08	2.29551E-05	TRUE	+
TAPBP	40.49993901	3.63243E-07	0.000626715	TRUE	-
TTC22	37.22402462	1.59238E-06	0.002354908	TRUE	+
TRHDE	35.82478145	2.98142E-06	0.003857955	TRUE	-
SUCLG2	35.03968139	4.23388E-06	0.004869905	TRUE	+
CCR9	34.57980169	5.19733E-06	0.005380279	TRUE	-
XAF1	33.97108634	6.81444E-06	0.006413004	TRUE	-
SOCS3	33.42120484	8.69955E-06	0.006902547	TRUE	+
PTPN3	33.26228091	9.33497E-06	0.006902547	FALSE	-
PPL	33.3502467	8.97778E-06	0.006902547	TRUE	+
VWF	32.87587228	1.10785E-05	0.007645644	TRUE	+
HMGCS1	32.72868894	1.18244E-05	0.007650407	TRUE	-
MYH4	31.31394572	2.20794E-05	0.01344507	TRUE	+
RPE65	30.92033756	2.62526E-05	0.013588353	TRUE	-
HMHA1	30.92224017	2.62307E-05	0.013588353	TRUE	-
SMARCD2	30.9279381	2.61651E-05	0.013588353	FALSE	-
PARP14	30.24006807	3.53853E-05	0.017443259	FALSE	-
THEMIS	25.36746459	4.24355E-05	0.019967845	TRUE	X
LDLR	29.47665108	4.94155E-05	0.022241283	TRUE	+
SLC5A7	24.6673321	5.86822E-05	0.025311594	TRUE	-
TBC1D10C	24.55151455	6.19107E-05	0.025635979	TRUE	-
TGM2	28.55042073	7.39872E-05	0.027734576	FALSE	+
APBA2	28.06170757	9.14814E-05	0.027734576	TRUE	-
TCEA2	28.12627619	8.89544E-05	0.027734576	TRUE	-
ELAVL2	28.37024832	8.00138E-05	0.027734576	TRUE	-
STMN2	28.46883519	7.66584E-05	0.027734576	TRUE	-
TTF2	28.1297397	8.88208E-05	0.027734576	TRUE	-
ARHGAP25	27.93975327	9.64495E-05	0.027734576	FALSE	-
TRIM54	27.94320845	9.63051E-05	0.027734576	FALSE	-
PIK3C2B	28.25286739	8.41986E-05	0.027734576	TRUE	-
TMEM97	28.32463944	8.16148E-05	0.027734576	FALSE	-
MFRP	24.23969556	7.15065E-05	0.027734576	TRUE	X
THRB	27.78313312	0.000103222	0.028183229	TRUE	-
KIF19	27.77794474	0.000103455	0.028183229	TRUE	-

INPP5D	27.70022303	0.000106996	0.028400566	TRUE	-
GNGT1	23.19002013	0.000116024	0.02901769	TRUE	-
CD53	27.4542457	0.000119012	0.02901769	FALSE	-
USH2A	27.58155305	0.000112635	0.02901769	TRUE	+
OVOL2	27.42486474	0.000120533	0.02901769	TRUE	+
TIFA	27.21628694	0.000131901	0.029465748	FALSE	-
FAN1	27.12419136	0.000137252	0.029465748	FALSE	-
C4A	22.78985784	0.000139473	0.029465748	TRUE	X
C4B	22.78985784	0.000139473	0.029465748	TRUE	X
KCNS1	27.28813898	0.00012787	0.029465748	TRUE	-
JAKMIP3	27.21143796	0.000132178	0.029465748	TRUE	-
FABP3	26.91482584	0.000150226	0.031102701	TRUE	-
TMEM72	26.81720228	0.000156682	0.031803369	FALSE	-
SLC39A10	26.73719811	0.000162177	0.032285644	TRUE	-
CEBPD	26.61426488	0.000170993	0.03277993	TRUE	-
C1QTNF7	26.61459442	0.000170968	0.03277993	FALSE	+
THRA	26.48613238	0.000180685	0.034008192	FALSE	-
IFI44	26.33327138	0.000192959	0.035669832	TRUE	+
MYH6	25.96315727	0.000226188	0.041078997	TRUE	-
MPEG1	25.80573566	0.000241978	0.042492558	FALSE	-
FCHO2	25.79174096	0.000243433	0.042492558	TRUE	+
COLEC12	25.76454024	0.000246286	0.042492558	FALSE	-
FKBP4	25.62893011	0.00026101	0.04429461	FALSE	+
ELF1	25.44852015	0.00028195	0.047076497	TRUE	-
GAPDH	25.33934258	0.000295417	0.048542206	FALSE	-
NEUROD6	25.29831383	0.000300641	0.048576121	TRUE	-
FAM101B	25.26453979	0.000305008	0.048576121	TRUE	-
PDE6B	25.20456039	0.00031292	0.049081062	FALSE	-

Supplementary Table S15. Statistical data for pathways enriched in subiculum of mdd vs. control groups using only orthologues 1:1:1 mapped between human, rat and zebrafish (Experiment 3). The KEGG- enrichment analysis was performed on normalized and log2-transformed counts by general applicable gene set enrichment for pathway analysis (GAGE) package [S4], using two-tailed t-test for group comparison of differential expression of gene sets. False discovery rate cutoff (q.val) was set at 0.05. p.geomean – geometric mean of the individual p-values from multiple single array based gene set tests, stat.mean - mean of the individual statistics from multiple single array based gene set tests. Pathways are listed as sorted by q value.

	**p.geomean**	**stat.mean**	**p.val**	**q.val**	**set.size**
Bidirected					
hsa04080 Neuroactive ligand-receptor interaction	4.53E-07	4.95713	1.95E-17	5.77E-15	190
hsa04512 ECM-receptor interaction	0.003851	2.701489	2.27E-06	0.000336	59
hsa04020 Calcium signaling pathway	0.003799	2.405548	1.85E-05	0.001821	171
hsa04974 Protein digestion and absorption	0.012619	2.266103	5.46E-05	0.003848	60
hsa04060 Cytokine-cytokine receptor interaction	0.011982	2.237656	6.5E-05	0.003848	78
hsa04151 PI3K-Akt signaling pathway	0.01561	2.143248	0.000108	0.005084	238
hsa04610 Complement and coagulation cascades	0.013341	2.174333	0.00012	0.005084	39
hsa05150 Staphylococcus aureus infection	0.02048	2.128722	0.000239	0.008842	16
hsa04742 Taste transduction	0.025507	1.943042	0.000493	0.014988	36
hsa04640 Hematopoietic cell lineage	0.026864	1.959298	0.000506	0.014988	22
hsa05033 Nicotine addiction	0.029813	1.845318	0.000932	0.025066	31
hsa04514 Cell adhesion molecules (CAMs)	0.040335	1.75408	0.001294	0.031909	71
hsa04950 Maturity onset diabetes of the young	0.043401	1.754742	0.001714	0.039023	17
Downregulated					
hsa05010 Alzheimer's disease	0.000337	-3.1049	4.82E-08	1.43E-05	263
hsa05016 Huntington's disease	0.000521	-2.98741	1.5E-07	1.71E-05	216
hsa05012 Parkinson's disease	0.000524	-2.95978	2.05E-07	1.71E-05	176
hsa05014 Amyotrophic lateral sclerosis (ALS)	0.000624	-2.93608	2.32E-07	1.71E-05	241
hsa05020 Prion diseases	0.000478	-2.91884	2.89E-07	1.71E-05	197
hsa00190 Oxidative phosphorylation	0.00215	-2.26604	6.64E-05	0.003277	93
hsa03050 Proteasome	0.016946	-2.14893	0.000142	0.006008	39
hsa04723 Retrograde endocannabinoid signaling	0.010977	-1.99861	0.000306	0.01132	120
hsa04961 Endocrine and other factor-regulated calcium reabsorption	0.021919	-1.80017	0.001225	0.040292	39

Supplementary Table S16. Statistical data for pathways enriched in hippocampus of stressed rats vs. control groups using only orthologues 1:1:1 mapped between human, rat and zebrafish (Experiment 3). The KEGG- enrichment analysis was performed on normalized and log2-transformed counts by general applicable gene set enrichment for pathway analysis (GAGE) package [S4], using two-tailed t-test for group comparison of differential expression of gene sets. False discovery rate cutoff (q.val) was set at 0.05. p.geomean – geometric mean of the individual p-values from multiple single array based gene set tests, stat.mean - mean of the individual statistics from multiple single array based gene set tests. Pathways are listed as sorted by q value.

	**p.geomean**	**stat.mean**	**p.val**	**q.val**	**set.size**
Bidirected					
hsa04080 Neuroactive ligand-receptor interaction	4.96E-08	5.383944	5.39E-20	1.59E-17	192
hsa04020 Calcium signaling pathway	3.35E-05	4.022018	3.27E-12	4.82E-10	171
hsa05205 Proteoglycans in cancer	0.003178	2.735122	1.29E-06	0.000127	157
hsa05200 Pathways in cancer	0.003917	2.654317	2.28E-06	0.000161	369
hsa04024 cAMP signaling pathway	0.004133	2.644801	2.73E-06	0.000161	161
hsa04512 ECM-receptor interaction	0.004598	2.647103	3.36E-06	0.000165	58
hsa04916 Melanogenesis	0.005195	2.566598	6.02E-06	0.000254	79
hsa04310 Wnt signaling pathway	0.007975	2.423094	1.57E-05	0.00058	119
hsa05414 Dilated cardiomyopathy	0.008904	2.392839	2.14E-05	0.0007	73
hsa04022 cGMP-PKG signaling pathway	0.011949	2.270973	4.69E-05	0.001384	115
hsa04725 Cholinergic synapse	0.011551	2.250127	5.71E-05	0.001418	92
hsa04360 Axon guidance	0.01163	2.232943	6.07E-05	0.001418	145
hsa05217 Basal cell carcinoma	0.01201	2.259081	6.25E-05	0.001418	53
hsa05410 Hypertrophic cardiomyopathy (HCM)	0.016679	2.147487	0.000117	0.002472	69
hsa04510 Focal adhesion	0.017245	2.103377	0.000144	0.002782	154
hsa04974 Protein digestion and absorption	0.013719	2.123501	0.000151	0.002782	59
hsa04390 Hippo signaling pathway	0.01789	2.084749	0.000168	0.002917	118
hsa04550 Signaling pathways regulating pluripotency of stem cells	0.018952	2.070649	0.000186	0.003049	106
hsa04925 Aldosterone synthesis and secretion	0.020185	2.045159	0.000226	0.003516	73
hsa04970 Salivary secretion	0.019625	2.013314	0.000302	0.00446	49
hsa05033 Nicotine addiction	0.025544	1.973838	0.000422	0.005931	31
hsa04151 PI3K-Akt signaling pathway	0.031389	1.853605	0.000681	0.008866	238
hsa04261 Adrenergic signaling in cardiomyocytes	0.031241	1.856926	0.000691	0.008866	110
hsa04713 Circadian entrainment	0.030422	1.854979	0.00073	0.00897	68
hsa04742 Taste transduction	0.035699	1.824367	0.000934	0.011021	37
hsa05031 Amphetamine addiction	0.038772	1.774578	0.00121	0.013731	46
hsa04971 Gastric acid secretion	0.042327	1.737823	0.001456	0.01544	49
hsa04015 Rap1 signaling pathway	0.041897	1.72349	0.001465	0.01544	160
hsa05224 Breast cancer	0.040842	1.716937	0.001547	0.015741	115
hsa04060 Cytokine-cytokine receptor interaction	0.044372	1.699749	0.001728	0.016996	77
hsa04728 Dopaminergic synapse	0.041237	1.65509	0.002214	0.021072	95
hsa04740 Olfactory transduction	0.04637	1.669576	0.002439	0.021882	23
hsa04911 Insulin secretion	0.051882	1.636751	0.002477	0.021882	56
hsa05412 Arrhythmogenic right ventricular cardiomyopathy (ARVC)	0.053253	1.62741	0.002591	0.021882	59
hsa05032 Morphine addiction	0.046565	1.62697	0.002596	0.021882	73

hsa04270 Vascular smooth muscle contraction	0.052136	1.619054	0.002688	0.022029	77
hsa04724 Glutamatergic synapse	0.056356	1.556455	0.003683	0.029366	85
hsa05030 Cocaine addiction	0.061718	1.562645	0.003826	0.029704	38
hsa05202 Transcriptional misregulation in cancer	0.062474	1.496723	0.004926	0.03726	114
Upregulated					
hsa03010 Ribosome	0.000541	2.842556	1.06E-06	0.000312	93
hsa05140 Leishmaniasis	0.015542	2.173171	0.000114	0.016805	40
hsa05145 Toxoplasmosis	0.01901	2.016395	0.000283	0.027796	66

Supplementary Table S17. Statistical data for pathways enriched in whole brain of stressed zebrafish vs. control groups using only orthologues 1:1:1 mapped between human, rat and zebrafish (Experiment 3). The KEGG- enrichment analysis was performed on normalized and log2-transformed counts by general applicable gene set enrichment for pathway analysis (GAGE) package [S4], using two-tailed t-test for group comparison of differential expression of gene sets. False discovery rate cutoff (q.val) was set at 0.05. p.geomean – geometric mean of the individual p-values from multiple single array based gene set tests, stat.mean - mean of the individual statistics from multiple single array based gene set tests. Pathways are listed as sorted by q value.

	**p.geomean**	**stat.mean**	**p.val**	**q.val**	**set.size**
Bidirected					
hsa05414 Dilated cardiomyopathy	0.004006	2.641433	1.01E-10	2.19E-08	73
hsa05032 Morphine addiction	0.003268	2.618746	1.7E-10	2.19E-08	73
hsa04080 Neuroactive ligand-receptor interaction	0.005036	2.559949	2.21E-10	2.19E-08	199
hsa05410 Hypertrophic cardiomyopathy (HCM)	0.007766	2.429903	2.27E-09	1.69E-07	69
hsa04024 cAMP signaling pathway	0.013014	2.162309	6.92E-08	4.11E-06	164
hsa04020 Calcium signaling pathway	0.017887	2.094285	1.62E-07	6.9E-06	175
hsa04724 Glutamatergic synapse	0.012755	2.115018	1.63E-07	6.9E-06	85
hsa04512 ECM-receptor interaction	0.019121	2.018571	5.42E-07	2.01E-05	60
hsa04261 Adrenergic signaling in cardiomyocytes	0.023224	1.935474	1.26E-06	3.79E-05	110
hsa04010 MAPK signaling pathway	0.018283	1.928344	1.28E-06	3.79E-05	223
hsa04510 Focal adhesion	0.027651	1.879222	2.29E-06	6.18E-05	156
hsa04742 Taste transduction	0.030154	1.879279	3.21E-06	7.94E-05	37
hsa05412 Arrhythmogenic right ventricular cardiomyopathy (ARVC)	0.030375	1.852914	3.61E-06	8.25E-05	59
hsa04380 Osteoclast differentiation	0.030327	1.839168	3.94E-06	8.37E-05	83
hsa04727 GABAergic synapse	0.027039	1.839619	4.29E-06	8.5E-05	70
hsa04912 GnRH signaling pathway	0.027123	1.815523	5.74E-06	0.000106	65
hsa04917 Prolactin signaling pathway	0.034688	1.754358	1.1E-05	0.000192	58
hsa05033 Nicotine addiction	0.034844	1.744305	1.53E-05	0.000253	31
hsa04723 Retrograde endocannabinoid signaling	0.025	1.699717	1.87E-05	0.000292	120
hsa04360 Axon guidance	0.032668	1.686751	1.98E-05	0.000294	145
hsa04713 Circadian entrainment	0.04079	1.670769	2.57E-05	0.000364	69
hsa05146 Amoebiasis	0.048949	1.632643	3.71E-05	0.0005	59
hsa04659 Th17 cell differentiation	0.054352	1.606018	4.76E-05	0.000614	60
hsa04930 Type II diabetes mellitus	0.04567	1.594037	6.25E-05	0.000765	40
hsa04913 Ovarian steroidogenesis	0.056635	1.585977	6.44E-05	0.000765	39
hsa04022 cGMP-PKG signaling pathway	0.056157	1.538202	8.82E-05	0.000987	118
hsa04668 TNF signaling pathway	0.059471	1.54041	8.97E-05	0.000987	71
hsa05133 Pertussis	0.042552	1.538207	0.000118	0.001254	43
hsa04725 Cholinergic synapse	0.054533	1.500017	0.000133	0.001364	92
hsa04726 Serotonergic synapse	0.062552	1.49424	0.000141	0.001395	76
hsa04610 Complement and coagulation cascades	0.032983	1.513534	0.00016	0.001535	46
hsa04740 Olfactory transduction	0.059184	1.492518	0.000194	0.001805	24
hsa04658 Th1 and Th2 cell differentiation	0.078117	1.403228	0.000328	0.00295	50
hsa04933 AGE-RAGE signaling pathway in diabetic complications	0.075228	1.374358	0.000414	0.003613	71
hsa04728 Dopaminergic synapse	0.068932	1.370059	0.000428	0.00363	95

hsa04921 Oxytocin signaling pathway	0.074252	1.350229	0.000499	0.004077	107
hsa04923 Regulation of lipolysis in adipocytes	0.078945	1.356623	0.000508	0.004077	45
hsa00604 Glycosphingolipid biosynthesis - ganglio series	0.058034	1.398154	0.000598	0.004676	13
hsa04925 Aldosterone synthesis and secretion	0.071949	1.323342	0.000647	0.004929	75
hsa04260 Cardiac muscle contraction	0.077302	1.321452	0.000683	0.005072	56
hsa04640 Hematopoietic cell lineage	0.090608	1.30384	0.000878	0.006361	23
hsa04730 Long-term depression	0.083144	1.285332	0.000933	0.006597	42
hsa00230 Purine metabolism	0.090769	1.270444	0.000977	0.006745	105
hsa05321 Inflammatory bowel disease (IBD)	0.094583	1.276452	0.001093	0.007335	23
hsa05162 Measles	0.091061	1.256781	0.001111	0.007335	83
hsa04630 Jak-STAT signaling pathway	0.092048	1.249675	0.00118	0.00762	73
hsa05145 Toxoplasmosis	0.108654	1.214563	0.001538	0.009721	70
hsa05418 Fluid shear stress and atherosclerosis	0.10953	1.190996	0.001831	0.011327	96
hsa04924 Renin secretion	0.104251	1.186261	0.002022	0.012255	44
hsa05030 Cocaine addiction	0.109751	1.177717	0.002165	0.01286	38
hsa04914 Progesterone-mediated oocyte maturation	0.113577	1.106904	0.003509	0.020426	78
hsa05142 Chagas disease (American trypanosomiasis)	0.124369	1.104028	0.003576	0.020426	70
hsa04151 PI3K-Akt signaling pathway	0.095503	1.096051	0.003718	0.020837	242
hsa04066 HIF-1 signaling pathway	0.137025	1.070691	0.004504	0.024772	78
hsa04015 Rap1 signaling pathway	0.141647	1.058095	0.004839	0.026132	162
hsa04911 Insulin secretion	0.110804	1.059492	0.005043	0.026744	58
hsa05161 Hepatitis B	0.140602	1.050694	0.005133	0.026747	118
hsa04514 Cell adhesion molecules (CAMs)	0.151312	1.025071	0.006188	0.031686	71
hsa04974 Protein digestion and absorption	0.124905	1.023798	0.006466	0.03255	63
hsa04371 Apelin signaling pathway	0.141862	1.012356	0.006764	0.033311	100
hsa00250 Alanine. aspartate and glutamate metabolism	0.14214	1.019662	0.006842	0.033311	31
hsa04976 Bile secretion	0.158373	1.001103	0.00737	0.035303	50
hsa05200 Pathways in cancer	0.137308	0.973487	0.008615	0.040611	374
hsa04918 Thyroid hormone synthesis	0.161346	0.969912	0.009092	0.041849	54
hsa04540 Gap junction	0.13844	0.968447	0.009188	0.041849	69
hsa04062 Chemokine signaling pathway	0.130343	0.965753	0.0093	0.041849	115
hsa04931 Insulin resistance	0.122016	0.962011	0.009616	0.042626	84
hsa04620 Toll-like receptor signaling pathway	0.166371	0.957473	0.00982	0.04289	55
hsa05212 Pancreatic cancer	0.150904	0.954291	0.010034	0.043189	66
hsa04660 T cell receptor signaling pathway	0.16839	0.946175	0.01047	0.044421	73
hsa05166 HTLV-I infection	0.172082	0.939943	0.010759	0.045008	158
hsa05221 Acute myeloid leukemia	0.160312	0.932233	0.011668	0.048129	52
Upregulated					
hsa00190 Oxidative phosphorylation	3.3E-05	2.024304	1.76E-06	0.000521	93
hsa01200 Carbon metabolism	0.029977	1.475358	0.000179	0.020542	100
hsa00010 Glycolysis / Gluconeogenesis	0.046397	1.46836	0.000207	0.020542	45
Downregulated					
hsa04380 Osteoclast differentiation	0.016547	-1.8477	3.95E-06	0.000779	83
hsa04659 Th17 cell differentiation	0.018426	-1.82853	5.24E-06	0.000779	60
hsa05032 Morphine addiction	0.007228	-1.68652	2.48E-05	0.002452	73
hsa04062 Chemokine signaling pathway	0.03853	-1.57102	6.7E-05	0.00417	115

hsa04658 Th1 and Th2 cell differentiation	0.041076	-1.55303	8.89E-05	0.00417	50
hsa04010 MAPK signaling pathway	0.003573	-1.52863	0.000101	0.00417	223
hsa04724 Glutamatergic synapse	0.01262	-1.54118	0.000106	0.00417	85
hsa05133 Pertussis	0.051107	-1.53122	0.000115	0.00417	43
hsa05168 Herpes simplex infection	0.030878	-1.50251	0.00014	0.00417	90
hsa04064 NF-kappa B signaling pathway	0.053472	-1.49877	0.00014	0.00417	61
hsa05162 Measles	0.030618	-1.47929	0.000174	0.004627	83
hsa04668 TNF signaling pathway	0.033729	-1.47285	0.000187	0.004627	71
hsa04660 T cell receptor signaling pathway	0.058261	-1.38726	0.000376	0.0086	73
hsa05340 Primary immunodeficiency	0.027182	-1.45371	0.000439	0.009304	21
hsa04713 Circadian entrainment	0.037009	-1.34787	0.000561	0.011101	69
hsa04630 Jak-STAT signaling pathway	0.071304	-1.26971	0.001027	0.018005	73
hsa04621 NOD-like receptor signaling pathway	0.070683	-1.26776	0.001031	0.018005	91
hsa00601 Glycosphingolipid biosynthesis - lacto and neolacto series	0.095572	-1.2792	0.001316	0.020753	12
hsa04060 Cytokine-cytokine receptor interaction	0.087552	-1.23497	0.001328	0.020753	83
hsa04930 Type II diabetes mellitus	0.036333	-1.22275	0.001783	0.025286	40
hsa04514 Cell adhesion molecules (CAMs)	0.065547	-1.20274	0.001788	0.025286	71
hsa05160 Hepatitis C	0.088347	-1.1755	0.002104	0.02841	105
hsa04727 GABAergic synapse	0.055078	-1.14958	0.002666	0.03442	70
hsa05145 Toxoplasmosis	0.093434	-1.14113	0.002782	0.034425	70
hsa05169 Epstein-Barr virus infection	0.074761	-1.12347	0.003128	0.037157	125
hsa05142 Chagas disease (American trypanosomiasis)	0.088941	-1.11071	0.003458	0.038429	70
hsa04742 Taste transduction	0.049971	-1.11725	0.003696	0.038429	37
hsa05152 Tuberculosis	0.112139	-1.09832	0.003715	0.038429	90
hsa04020 Calcium signaling pathway	0.011845	-1.09929	0.003752	0.038429	175
hsa04728 Dopaminergic synapse	0.068908	-1.06643	0.004694	0.046472	95
hsa05321 Inflammatory bowel disease (IBD)	0.097972	-1.07618	0.005131	0.048228	23
hsa04360 Axon guidance	0.007494	-1.05135	0.005254	0.048228	145
hsa04725 Cholinergic synapse	0.071482	-1.0474	0.005434	0.048228	92
hsa04622 RIG-I-like receptor signaling pathway	0.108817	-1.05413	0.005521	0.048228	38

Supplementary Table S18. Summary of statistical analyses of Fisher meta-analysis between pathways analyzed using general applicable gene set enrichment for pathway analysis (GAGE) package [S4] function of human (Supplementary Table S11), rat (Supplementary Table S12) and zebrafish (Supplementary Table S13) vs. control groups using only orthologues 1:1:1 mapped between human, rat and zebrafish (Experiment 3) using the metaRNASeq R package [S5]. P value and false discovery rate were set at 0.05. Pathways in this table are listed as sorted by their combined p-adjusted value and further compared using similarity in log 2-fold change direction between all species.

	**fishcombg.TestStatistic**	**fishcombg.rawpval**	**fishcombg.adjpval**
Bidirected			
hsa04020 Calcium signaling pathway	105.9646	0	0
hsa04080 Neuroactive ligand-receptor interaction	210.1503	0	0
hsa04512 ECM-receptor interaction	80.05214	3.44E-15	3.41E-13
hsa05414 Dilated cardiomyopathy	71.98025	1.6E-13	1.19E-11
hsa04024 cAMP signaling pathway	69.10555	6.24E-13	3.7E-11
hsa05410 Hypertrophic cardiomyopathy (HCM)	63.42645	9.04E-12	4.47E-10
hsa05032 Morphine addiction	58.82909	7.78E-11	3.3E-09
hsa04742 Taste transduction	54.48003	5.9E-10	2.19E-08
hsa05033 Nicotine addiction	51.67058	2.17E-09	7.17E-08
hsa04510 Focal adhesion	48.96895	7.56E-09	2.25E-07
hsa04974 Protein digestion and absorption	47.31149	1.62E-08	4.38E-07
hsa04010 MAPK signaling pathway	46.64594	2.2E-08	5.03E-07
hsa04724 Glutamatergic synapse	46.70773	2.14E-08	5.03E-07
hsa04360 Axon guidance	46.24113	2.65E-08	5.62E-07
hsa05200 Pathways in cancer	45.21253	4.25E-08	8.41E-07
hsa04725 Cholinergic synapse	44.98379	4.72E-08	8.75E-07
hsa04261 Adrenergic signaling in cardiomyocytes	44.63607	5.53E-08	9.66E-07
hsa04022 cGMP-PKG signaling pathway	43.82068	8.02E-08	1.19E-06
hsa04151 PI3K-Akt signaling pathway	44.04572	7.24E-08	1.19E-06
hsa05412 Arrhythmogenic right ventricular cardiomyopathy (ARVC)	43.8251	8.01E-08	1.19E-06
hsa04610 Complement and coagulation cascades	40.46671	3.69E-07	5.22E-06
hsa04060 Cytokine-cytokine receptor interaction	40.13629	4.28E-07	5.78E-06
hsa04713 Circadian entrainment	39.55435	5.57E-07	7.2E-06
hsa04925 Aldosterone synthesis and secretion	38.77609	7.92E-07	9.8E-06
hsa05205 Proteoglycans in cancer	38.33155	9.68E-07	1.15E-05
hsa04726 Serotonergic synapse	36.98682	1.77E-06	2.02E-05
hsa05146 Amoebiasis	36.56575	2.14E-06	2.35E-05
hsa04380 Osteoclast differentiation	34.17577	6.22E-06	6.25E-05
hsa04727 GABAergic synapse	34.24353	6.04E-06	6.25E-05
hsa05150 Staphylococcus aureus infection	34.14331	6.31E-06	6.25E-05
hsa04912 GnRH signaling pathway	33.79614	7.37E-06	7.06E-05
hsa04514 Cell adhesion molecules (CAMs)	32.36212	1.39E-05	0.000125
hsa04913 Ovarian steroidogenesis	32.42519	1.35E-05	0.000125
hsa04917 Prolactin signaling pathway	32.19343	1.5E-05	0.000131

hsa05030 Cocaine addiction	31.98865	1.64E-05	0.000139
hsa04640 Hematopoietic cell lineage	31.29279	2.23E-05	0.000181
hsa04916 Melanogenesis	31.2706	2.25E-05	0.000181
hsa04740 Olfactory transduction	31.17152	2.35E-05	0.000184
hsa05031 Amphetamine addiction	30.84206	2.72E-05	0.000207
hsa04668 TNF signaling pathway	30.5511	3.09E-05	0.000224
hsa04728 Dopaminergic synapse	30.6059	3.01E-05	0.000224
hsa04923 Regulation of lipolysis in adipocytes	29.95212	4.01E-05	0.000284
hsa04310 Wnt signaling pathway	29.45822	4.98E-05	0.000344
hsa04390 Hippo signaling pathway	29.00879	6.06E-05	0.000402
hsa05133 Pertussis	28.99786	6.09E-05	0.000402
hsa04015 Rap1 signaling pathway	28.44488	7.75E-05	0.0005
hsa04723 Retrograde endocannabinoid signaling	28.35024	8.07E-05	0.00051
hsa04933 AGE-RAGE signaling pathway in diabetic complications	28.28011	8.32E-05	0.000515
hsa04976 Bile secretion	27.56954	0.000113	0.000686
hsa04918 Thyroid hormone synthesis	27.29267	0.000128	0.000758
hsa04550 Signaling pathways regulating pluripotency of stem cells	27.10532	0.000138	0.00079
hsa04921 Oxytocin signaling pathway	27.11268	0.000138	0.00079
hsa05217 Basal cell carcinoma	27.00294	0.000145	0.00081
hsa04924 Renin secretion	26.58914	0.000173	0.000951
hsa05144 Malaria	26.22524	0.000202	0.001091
hsa04911 Insulin secretion	24.70662	0.000387	0.002052
hsa00830 Retinol metabolism	24.37948	0.000445	0.002317
hsa04930 Type II diabetes mellitus	23.92512	0.000539	0.00276
hsa04970 Salivary secretion	23.70895	0.000591	0.002974
hsa04659 Th17 cell differentiation	23.26234	0.000713	0.00353
hsa04971 Gastric acid secretion	22.77132	0.000877	0.004269
hsa04270 Vascular smooth muscle contraction	22.66911	0.000915	0.004349
hsa05418 Fluid shear stress and atherosclerosis	22.65042	0.000922	0.004349
hsa05224 Breast cancer	22.25349	0.001089	0.005055
hsa04260 Cardiac muscle contraction	22.20347	0.001112	0.005082
hsa04014 Ras signaling pathway	21.78271	0.001326	0.005877
hsa05202 Transcriptional misregulation in cancer	21.78376	0.001325	0.005877
hsa04658 Th1 and Th2 cell differentiation	21.2982	0.001621	0.007082
hsa04744 Phototransduction	21.22448	0.001672	0.007196
hsa05320 Autoimmune thyroid disease	16.8558	0.002062	0.008747
hsa05204 Chemical carcinogenesis	20.6245	0.002142	0.008962
hsa04750 Inflammatory mediator regulation of TRP channels	20.2076	0.002543	0.010491
hsa00140 Steroid hormone biosynthesis	19.58813	0.003278	0.013154
hsa04371 Apelin signaling pathway	19.59575	0.003267	0.013154
hsa04540 Gap junction	18.5654	0.004964	0.019658
hsa05142 Chagas disease (American trypanosomiasis)	18.24778	0.005642	0.022048
hsa04721 Synaptic vesicle cycle	17.63896	0.007201	0.027774
hsa05321 Inflammatory bowel disease (IBD)	17.57169	0.007397	0.028164
hsa04670 Leukocyte transendothelial migration	17.17255	0.00867	0.032513
hsa02010 ABC transporters	17.1471	0.008758	0.032513

hsa04062 Chemokine signaling pathway	16.93558	0.009523	0.034917
hsa04730 Long-term depression	16.34444	0.01202	0.043535
hsa04630 Jak-STAT signaling pathway	16.12578	0.013095	0.046856
hsa05140 Leishmaniasis	16.08239	0.013319	0.04709
hsa05416 Viral myocarditis	15.90752	0.014259	0.049823
Upregulated			
hsa05020 Prion diseases	40.08984	4.37E-07	0.00013
hsa05010 Alzheimer's disease	37.1653	1.63E-06	0.000243
hsa04380 Osteoclast differentiation	34.05156	6.57E-06	0.000421
hsa05012 Parkinson's disease	34.48451	5.42E-06	0.000421
hsa05016 Huntington's disease	33.88408	7.08E-06	0.000421
hsa05032 Morphine addiction	32.78478	1.15E-05	0.000571
hsa00190 Oxidative phosphorylation	32.13882	1.53E-05	0.000651
hsa04659 Th17 cell differentiation	31.32877	2.19E-05	0.000655
hsa05014 Amyotrophic lateral sclerosis (ALS)	31.76985	1.81E-05	0.000655
hsa03010 Ribosome	31.31553	2.21E-05	0.000655
hsa04724 Glutamatergic synapse	30.01513	3.9E-05	0.001054
hsa05145 Toxoplasmosis	28.7661	6.74E-05	0.001667
hsa04062 Chemokine signaling pathway	27.44244	0.00012	0.002733
hsa05168 Herpes simplex infection	26.8711	0.000153	0.003248
hsa04723 Retrograde endocannabinoid signaling	26.30394	0.000195	0.003869
hsa05140 Leishmaniasis	26.10684	0.000213	0.003948
hsa04010 MAPK signaling pathway	25.54392	0.000271	0.004729
hsa04668 TNF signaling pathway	24.85618	0.000363	0.00599
hsa04727 GABAergic synapse	23.84573	0.000558	0.008404
hsa05340 Primary immunodeficiency	23.81023	0.000566	0.008404
hsa05133 Pertussis	23.6611	0.000603	0.008525
hsa04217 Necroptosis	23.28641	0.000706	0.009531
hsa04713 Circadian entrainment	23.10332	0.000763	0.009848
hsa05142 Chagas disease (American trypanosomiasis)	22.84493	0.00085	0.01052
hsa04621 NOD-like receptor signaling pathway	22.499	0.000983	0.011676
hsa03050 Proteasome	22.03266	0.001194	0.01267
hsa04064 NF-kappa B signaling pathway	22.05104	0.001185	0.01267
hsa04658 Th1 and Th2 cell differentiation	22.09824	0.001162	0.01267
hsa05162 Measles	21.25339	0.001652	0.016917
hsa04932 Non-alcoholic fatty liver disease (NAFLD)	21.09673	0.001763	0.01745
hsa04145 Phagosome	20.89385	0.001917	0.017792
hsa04730 Long-term depression	20.91745	0.001898	0.017792
hsa04660 T cell receptor signaling pathway	20.24601	0.002503	0.021688
hsa04930 Type II diabetes mellitus	20.29055	0.002458	0.021688
hsa05152 Tuberculosis	20.19559	0.002556	0.021688
hsa04014 Ras signaling pathway	19.48371	0.00342	0.028216
hsa04144 Endocytosis	19.21801	0.003811	0.03059
hsa04514 Cell adhesion molecules (CAMs)	19.11158	0.003979	0.031101
hsa04060 Cytokine-cytokine receptor interaction	18.93353	0.004277	0.032574
hsa04725 Cholinergic synapse	18.86304	0.004401	0.03268

hsa04961 Endocrine and other factor-regulated calcium reabsorption	18.60404	0.004887	0.035403
hsa04630 Jak-STAT signaling pathway	18.47489	0.005149	0.035563
hsa04726 Serotonergic synapse	18.52047	0.005055	0.035563
hsa04020 Calcium signaling pathway	18.05807	0.006089	0.041099
hsa05167 Kaposi's sarcoma-associated herpesvirus infection	17.95853	0.006337	0.041822
hsa04022 cGMP-PKG signaling pathway	17.78517	0.006792	0.042026
hsa04620 Toll-like receptor signaling pathway	17.79986	0.006752	0.042026
hsa01521 EGFR tyrosine kinase inhibitor resistance	17.78522	0.006792	0.042026
hsa04662 B cell receptor signaling pathway	17.68912	0.007058	0.042202
hsa04728 Dopaminergic synapse	17.67258	0.007105	0.042202
hsa05169 Epstein-Barr virus infection	17.59996	0.007314	0.042591
hsa05160 Hepatitis C	17.4824	0.007665	0.043777
hsa05200 Pathways in cancer	17.38825	0.007957	0.044592
hsa01200 Carbon metabolism	17.32899	0.008147	0.04481
hsa04612 Antigen processing and presentation	17.08435	0.008978	0.048023
hsa04921 Oxytocin signaling pathway	17.06292	0.009055	0.048023

Supplementary Table S19. Statistical data for maximal cliques identified in PPI of orthologues 1:1:1 mapped between human, rat and zebrafish expression enriched in whole brain of stressed zebrafish vs. control groups (Experiment 4). The enrichment analysis was performed on normalized and log2-transformed counts by general applicable gene set enrichment for pathway analysis (GAGE) package [S4], using two-tailed t-test for group comparison of differential expression of gene sets. False discovery rate cutoff (q.val) was set at 0.05. p.geomean – geometric mean of the individual p-values from multiple single array based gene set tests, stat.mean - mean of the individual statistics from multiple single array based gene set tests. Pathways are listed as sorted by q value.

	**p.geomean**	**stat.mean**	**p.val**	**q.val**	**set.size**
KCNH1 KCNA4 KCNA2 KCNB1 KCNH5 KCNA5	0.0106157840072457	3.03555355179913	3.65324140785854e-08	0.000243425143118951	5
KCNH1 KCNA1 KCNA4 KCNA2 KCNB2 KCNB1 KCNH5	0.010603264724963	2.70373140653806	4.04831437084568e-08	0.000243425143118951	6
KCNH1 KCNA1 KCNA4 KCNA2 KCNA3 KCNB1 KCNH5	0.010603264724963	2.70373140653806	4.04831437084568e-08	0.000243425143118951	6
KCNH1 KCNA1 KCNAB2 KCNA4 KCNA2 KCNA3 KCNH5	0.0145745190830216	2.53839141155662	1.37175791960253e-07	0.000522957188700021	6
KCNH1 KCNAB2 KCNA4 KCNA2 KCNH5 KCNA5	0.0151795036057337	2.76262290262762	1.4495182346583e-07	0.000522957188700021	5
KCNH1 KCNAB1 KCNA4 KCNA2 KCNH5 KCNA5	0.0141317750097448	2.67291639777968	1.84070800620201e-07	0.000553408862064634	5
KCNH1 KCNA1 KCNAB1 KCNA4 KCNA2 KCNA3 KCNH5	0.0148874322347224	2.48037829786819	2.63165818084099e-07	0.000594316375446355	6
STAT5A MAPK1 STAT5B FYN SRC ABL1 STAT3	0.0148741206716595	2.67289876267808	2.75615482925788e-07	0.000594316375446355	6
KCNA4 KCNH3 KCNA2 KCNB1 KCNA5	0.0134486676070157	3.07045983780521	2.96515736959764e-07	0.000594316375446355	4
KCNA4 KCNA2 KCNH7 KCNB1 KCNA5	0.0141261666853662	3.26158999274572	3.39407369419714e-07	0.000612256953696223	4
KCNA1 KCNA4 KCNA2 KCNB2 KCNH7 KCNB1	0.0146494146899095	2.61445980876338	4.08894187165423e-07	0.000614670186856422	5
KCNA1 KCNA4 KCNA2 KCNA3 KCNH7 KCNB1	0.0146494146899095	2.61445980876338	4.08894187165423e-07	0.000614670186856422	5
PIK3CA FYN CDC42 SRC KDR CBL	0.0166197691967684	2.7840885811051	5.80552754127501e-07	0.000805583933208152	5
KCNA1 KCNA4 KCNH3 KCNA2 KCNA3 KCNB1	0.0159613734674627	2.53536194623945	7.13794234021981e-07	0.0008524048868563	5
KCNA1 KCNA4 KCNH3 KCNA2 KCNB2 KCNB1	0.0159613734674627	2.53536194623945	7.13794234021981e-07	0.0008524048868563	5
FYN CDC42 SRC PXN KDR CBL	0.0173010720043824	2.74060905071971	7.560551133489e-07	0.0008524048868563	5
KCNAB2 KCNA4 KCNA2 KCNH7 KCNA5	0.019983560881734	2.8481471970406	1.04489707358456e-06	0.00110875872414069	4
KCNH1 KCNA1 KCNAB3 KCNA4 KCNA2 KCNH5	0.022798448711361	2.35712118081079	1.14063049113595e-06	0.00114310185720008	5
KCNA1 KCNAB2 KCNA4 KCNA2 KCNA3 KCNH7	0.0206958978953667	2.42688821555607	1.32965908106472e-06	0.0012624063243856	5
KCNA1 KCNA4 KCNA2 KCNH8 KCNA3 KCNB1	0.0234920961204341	2.33395676368973	1.676507152196e-06	0.00144011964373636	5
KCNA1 KCNA4 KCNA2 KCNB2 KCNH8 KCNB1	0.0234920961204341	2.33395676368973	1.676507152196e-06	0.00144011964373636	5
KCNAB2 KCNA4 KCNH3 KCNA2 KCNA5	0.0231314865000845	2.67590408102833	2.08443285173109e-06	0.00170914019147169	4
KCNA4 KCNA2 KCNH8 KCNB1 KCNA5	0.0251682210905271	2.71318770605943	2.20413696166026e-06	0.00172871420223432	4
KCNA1 KCNAB2 KCNA4 KCNH3 KCNA2 KCNA3	0.0229443065707345	2.35461864775062	2.37562716839077e-06	0.00174169103995837	5
KCNAB1 KCNA4 KCNH3 KCNA2 KCNA5	0.0191439328545855	2.82988932089346	2.41378546476851e-06	0.00174169103995837	4
KCNA1 KCNAB1 KCNA4 KCNA2 KCNA3 KCNH7	0.0205277136243906	2.37075831788289	2.63720650094015e-06	0.00182971415655613	5

KCNAB1 KCNA4 KCNA2 KCNH7 KCNA5	0.021528894433682	2.85928828304505	2.97965297261232e-06	0.00199073925825755	4
KCNH1 KCNQ2 KCNQ3 KCNH5	0.0263560805163466	2.57254337466567	3.66396757075287e-06	0.00229240860365868	4
KCNA1 KCNAB1 KCNA4 KCNH3 KCNA2 KCNA3	0.0209650164648062	2.33386701272285	3.6853400690782e-06	0.00229240860365868	5
KCNA1 KCNAB2 KCNA4 KCNA2 KCNH8 KCNA3	0.0300536357740991	2.19361614653757	5.01506456231482e-06	0.00295390626303191	5
KCNH1 KCNA1 KCNA4 KCNA2 KCNF1 KCNA3 KCNH5	0.0325272064484206	2.07550585981722	5.07628439237148e-06	0.00295390626303191	6
KCNH1 KCNA4 KCNA2 KCNF1 KCNH5 KCNA5	0.0341792923303888	2.18194912566365	6.5344215410516e-06	0.00368357594309468	5
KCNAB2 KCNA4 KCNA2 KCNH8 KCNA5	0.0334022323377084	2.47304243406485	7.74437963004967e-06	0.00423335951958988	4
SOCS2 STAT5B IGF1R STAT3	0.0292749576574358	2.51208178222335	9.59161854894568e-06	0.0050889178530715	4
KCNA1 KCNAB1 KCNA4 KCNA2 KCNH8 KCNA3	0.034024589496647	2.08187010693346	1.02616834734632e-05	0.00515483001431066	5
KCNAB1 KCNA4 KCNA2 KCNH8 KCNA5	0.0345041961424674	2.22410095680588	1.0287370725383e-05	0.00515483001431066	4
FLT4 NOTCH1 SRC FLT1 KDR	0.0390237560715826	2.28562780708956	1.12792968249653e-05	0.00549911447096076	4
KCNQ2 KCNH7 KCNQ3	0.0268882921531934	3.19475213848241	1.19307472811536e-05	0.00566365132117709	3
KCNA1 KCNAB3 KCNA4 KCNA2 KCNH7	0.033295916194942	2.23558913752213	1.25616981712963e-05	0.00581026854646191	4
KCNH1 KCNQ5 KCNQ3 KCNH5	0.0398935018377893	2.30468897224745	1.64498183554723e-05	0.00741845683285913	4
UBC MAPK1 FYN SRC HSP90AA1 ABL1 STAT3	0.0369214768411045	1.98666874498537	2.07307124663288e-05	0.00912100785805133	6
KCNA1 KCNAB3 KCNA4 KCNH3 KCNA2	0.036221605379724	2.1565478297019	2.14669957333166e-05	0.00922007466745947	4
FYN EPHB2 SRC EFNA5	0.0324928730382845	2.32076907660302	2.20396954750826e-05	0.00924590852732594	4
KCNQ2 KCNH3 KCNQ3	0.0314053095378088	3.13751598367506	2.36254306099602e-05	0.00958620277159428	3
KCNA1 KCNH2 KCNA4 KCNA2 KCNB2 KCNB1	0.0379402491046978	2.00218936661655	2.44451093460467e-05	0.00958620277159428	5
KCNA1 KCNH2 KCNA4 KCNA2 KCNA3 KCNB1	0.0379402491046978	2.00218936661655	2.44451093460467e-05	0.00958620277159428	5
SOCS3 STAT1 SOCS2 STAT5B LCK STAT3	0.0438202620735516	1.895061282025	2.8181130679808e-05	0.0108161577943203	6
MAPK1 FOS MAPK8 FYN SRC ABL1 STAT3	0.0409796588551673	1.84801141910084	3.18668073298364e-05	0.0119759445296441	7
KCNH2 KCNA4 KCNA2 KCNB1 KCNA5	0.0424584933895313	2.18609271562884	3.84372963142148e-05	0.0141504160859616	4
STAT5B SOCS1 ABL1 STAT3	0.0481682296977267	2.08139370780372	4.46136145900948e-05	0.0160956998718144	4
UBC IKBKB RELA PRKCZ SRC	0.0405767476760771	2.14584636252115	4.69878937767172e-05	0.0166198944282	4
KCNA1 KCNH2 KCNAB2 KCNA4 KCNA2 KCNA3	0.0482755193296408	1.86647275989609	5.09228424427164e-05	0.0171950437090939	5
KCNA1 KCNA4 KCNA2 KCNF1 KCNA3 KCNH7	0.0490251259851447	1.86775836988257	5.1284255777365e-05	0.0171950437090939	5
KCNA1 KCNAB3 KCNA4 KCNA2 KCNH8	0.0512255927767679	1.93318019324074	5.14736049831515e-05	0.0171950437090939	4
EFNB1 FYN PTPN13 EPHB2 SRC	0.0485264987338117	1.86173103522595	5.76865856936023e-05	0.018920151260489	5
MAPK10 MAPK9 DUSP10 MAP2K7	0.0481989305634947	2.15463287625437	6.10801193268765e-05	0.0193647227207535	4
RAC1 MAPK1 MAPK8 FYN SRC ABL1 STAT3	0.0449533563882213	1.78049325384647	6.12998431530044e-05	0.0193647227207535	7
SEMA3A NRP1 PLXNA2	0.0527555743014494	2.24084617085048	6.22944109662385e-05	0.0193647227207535	3
KCNH1 KCNB2 KCNG3 KCNB1 KCNH5	0.0495930180358052	2.07547490918402	6.38183869684404e-05	0.0193647227207535	4
KCNA4 KCNA2 KCNF1 KCNH7 KCNA5	0.0514419103081128	1.99178026675665	6.44095217720058e-05	0.0193647227207535	4

MYO9A ITSN1 RHOBTB1	0.0487717759664266	2.42313087536613	6.64829127324249e-05	0.0196604141439379	3
KCNH1 KCNG1 KCNB1 KCNH5	0.0555602238385026	1.93153188889938	7.44127683932862e-05	0.0210281972979009	4
STAT5A STAT5B FYN PDGFRB SRC ABL1 STAT3	0.0473198016122053	1.78633191110284	7.49662128851383e-05	0.0210281972979009	6
UBC MAPK1 FOS FYN SRC ABL1 STAT3	0.0534551658903192	1.75557289618344	7.4996278005998e-05	0.0210281972979009	6
KCNH2 KCNAB2 KCNA4 KCNA2 KCNA5	0.0535691429423241	1.95500462198846	7.57709864384698e-05	0.0210281972979009	4
KCNA1 KCNA4 KCNH3 KCNA2 KCNF1 KCNA3	0.053585613724494	1.79914725921609	8.34394087974703e-05	0.0228055075045086	5
KCNA1 KCNAB1 KCNH2 KCNA4 KCNA2 KCNA3	0.0479291963555386	1.82964736618019	9.15850290485112e-05	0.0246582438657626	5
MAPK1 STAT5B IGF1R PTPN11 SRC STAT3	0.0534009900884075	1.74722194462787	9.73684236868852e-05	0.0256431668059779	6
FYN CDC42 SRC PXN ABL1 BCAR1 CBL	0.0384620951130291	1.8273493825165	9.90520153094047e-05	0.0256431668059779	6
MAPK1 MAPK8 JAK3 LCK STAT3	0.0522601520891555	1.8223582789066	0.000100614541064959	0.0256431668059779	5
JAK1 STAT5A TYK2 SOCS3 STAT5B JAK3 STAT6 STAT3	0.0596336363646649	1.68167949809889	0.000101332483407035	0.0256431668059779	7
STAT5A SOCS3 JAK2 STAT5B JAK3 STAT6 STAT3	0.0584065851019009	1.72267503673599	0.000102583948922002	0.0256431668059779	6
KCNA4 KCNH3 KCNA2 KCNF1 KCNA5	0.0563124850829004	1.88003479169317	0.000103772447299539	0.0256431668059779	4
LCP2 GRAP2 ITK FYN ZAP70 CBL	0.0563297946641182	1.8065861763899	0.000108131618534556	0.0263592738749305	5
UBC NOTCH1 ARRB1 SRC	0.0544937805174363	2.23257532903848	0.000111708928877462	0.0265147022107966	3
AKT1 NOTCH1 ARRB1 SRC	0.0544937805174363	2.23257532903848	0.000111708928877462	0.0265147022107966	3
YES1 FYN EPHB2 EFNA5	0.0514594108374826	2.37257792451044	0.000113778814628378	0.0266552732088481	3
KCNQ5 KCNH7 KCNQ3	0.0537351816321546	2.36990462620463	0.000119301887251892	0.0275908556940625	3
STAT5A SOCS3 JAK2 STAT5B STAT6 SRC STAT3	0.0541187168983059	1.73441759729169	0.000122360308015654	0.027720552952091	6
KCNA1 KCNA4 KCNA2 KCNH8 KCNF1 KCNA3	0.0640769181912282	1.70123701104759	0.000122936096023465	0.027720552952091	5
MAPK8 MAPK9 DUSP10 MAP2K7	0.0579505026423943	1.91587782869129	0.00013679206972591	0.0304641005652555	4
UBC RAC1 MAPK1 FYN SRC ABL1 STAT3	0.0590454196018439	1.68087895306192	0.000144739585759855	0.0318409437502686	6
UBC NOTCH1 ITCH ERBB4	0.0575401239735188	2.25528433632668	0.000148292073007125	0.0319142116401691	3
KCNQ5 KCNH3 KCNQ3	0.0556486606975145	2.14766269905812	0.000148610997160275	0.0319142116401691	3
NCK2 EFNB1 FYN EPHB2 SRC	0.0605535110113515	1.71373661755555	0.000152593691458531	0.0323839717672993	5
KCNH1 KCND3 KCNB2 KCNB1 KCNH5	0.0568655464120165	1.94401444949145	0.000158131560412207	0.0331690141659977	4
KCNAB1 KCNH2 KCNA4 KCNA2 KCNA5	0.0571341285052754	1.96329738731107	0.000177859917615551	0.0355613629597926	4
FLT4 ANGPT2 NOTCH1 FLT1 KDR	0.0720275634681416	1.73761572371542	0.000181884918229436	0.0355613629597926	4
KCNH1 KCNB1 KCNH5 KCNS3	0.0677290639724265	1.75684694568989	0.000182796999958772	0.0355613629597926	4
EFNB1 FYN SRC EPHA5	0.062762113469489	2.03281497732606	0.000184642304462477	0.0355613629597926	3
EFNB1 EPHA6 FYN SRC	0.062762113469489	2.03281497732606	0.000184642304462477	0.0355613629597926	3
KCNH1 KCNQ4 KCNQ3 KCNH5	0.0678427786417247	2.06731044432695	0.000185718593202574	0.0355613629597926	3
KCNA4 KCNA2 KCNH8 KCNF1 KCNA5	0.0691848445387379	1.76271601074376	0.000186787365767867	0.0355613629597926	4
KCNH1 KCNA10 KCNB1 KCNH5	0.0665058700936904	2.13784534460859	0.000191221919568706	0.0355613629597926	3

KCNH1 KCNB1 KCNH5 KCND2	0.0665058700936904	2.13784534460859	0.000191221919568706	0.0355613629597926	3
KCNH1 KCNV1 KCNB1 KCNH5	0.0665058700936904	2.13784534460859	0.000191221919568706	0.0355613629597926	3
KCNH1 KCNB1 KCNH5 KCNG2	0.0665058700936904	2.13784534460859	0.000191221919568706	0.0355613629597926	3
RAC1 MAPK1 MAPK8 FYN SRC PXN ABL1	0.0513992300069138	1.64834196864766	0.000201405811237371	0.0370730553970504	7
MYO9A RHOBTB1 ARHGEF3	0.0624048750293838	2.13827001460911	0.000210964677118741	0.0384403213186361	3
NCAM1 FYN KIT	0.0653934024738645	2.0752113395537	0.000213312708815389	0.038479479543208	3
SOCS3 STAT1 STAT5B PTPN11 JAK3 LCK STAT3	0.0711522064840302	1.56662497153599	0.000224964287651006	0.0394451563086468	7
KCNA1 KCNH4 KCNA4 KCNA2 KCNA3 KCNB1	0.061108557345402	1.67759767311146	0.000225225960407485	0.0394451563086468	5
KCNA1 KCNH4 KCNA4 KCNA2 KCNB2 KCNB1	0.061108557345402	1.67759767311146	0.000225225960407485	0.0394451563086468	5
KCNQ2 KCNH8 KCNQ3	0.0671985536329718	2.26899352378063	0.00023295733482367	0.0404068977200402	3
UBC MAPK1 IGF1R SRC HSP90AA1 STAT3	0.0636736205139766	1.67778318541969	0.0002399201381046	0.0412182797263703	5
KCNA1 KCNA4 KCNA2 KCNA3 KCNB1 KCNH6	0.0682032722810909	1.63392752871746	0.000256914868781882	0.0429363129394485	5
KCNA1 KCNA4 KCNA2 KCNB2 KCNB1 KCNH6	0.0682032722810909	1.63392752871746	0.000256914868781882	0.0429363129394485	5
JAK1 TYK2 STAT5B SOCS1 JAK3 STAT6 STAT3	0.0715569825707676	1.55318386451139	0.000258750933274374	0.0429363129394485	7
YES1 EFNB1 FYN PTPN13 EPHB2	0.0708334641025282	1.7082224388208	0.000259441105959304	0.0429363129394485	4
YES1 ITK FYN ZAP70 CBL	0.0705050439590707	1.87983101160174	0.0002629474445309	0.0431209904717537	4
JAK2 STAT5B SOCS1 JAK3 STAT6 STAT3	0.0689690398763337	1.58694126976284	0.00027884183616516	0.0453155665097596	6
STAT5B SOCS1 FLT3 STAT3	0.0721175088261811	1.70034119979645	0.000296211199945305	0.0477085163911906	4

Supplementary Table S20. Statistical data of Fisher meta-analysis between for maximal cliques identified in orthologues 1:1:1 mapped between human, rat and zebrafish expression analyzed using general applicable gene set enrichment for pathway analysis (GAGE) package [S4] (Experiment 4) using the metaRNASeq R package [S5]. P value and false discovery rate were set at 0.05. Pathways in this table are listed as sorted by their combined p-adjusted value and further compared using similarity in log 2-fold change direction between all species.

	**fishcombg.TestStatistic**	**fishcombg.rawpval**	**fishcombg.adjpval**
WNT7B WNT9A SFRP1 SFRP2	59.6447367516849	5.31458210772939e-11	7.96561679838881e-07
PLCG2 SYK PIK3CG RAC2 PIK3CD	58.243451300239	1.02282959879574e-10	7.96561679838881e-07
NGFR RTN4R MAG LINGO1	57.6892502981525	1.32473254588206e-10	7.96561679838881e-07
AR SMAD3 FOS JUN RUNX2	54.6615955793397	5.42517475388138e-10	2.44661818463165e-06
MYO9B CDC42 RHOC ARHGEF16 RAC2	53.8718863376468	7.82927056519611e-10	2.82464423451145e-06
WNT7B WNT8B SFRP1 SFRP2	53.1209410694568	1.10929954111327e-09	3.33510907035706e-06
COL1A2 ITGA2 COL1A1	52.1095541458008	1.7725763079568e-09	4.56792914560467e-06
CDC42 RHOC RAC2 ARAP3	51.6632542913258	2.17938378455074e-09	4.91423801118884e-06
MYO9B CDC42 RHOC NGEF RAC2	51.2335509995839	2.65869937177143e-09	5.32891977415388e-06
MYO9B ARHGEF16 RHOB RAC2	50.5014421974029	3.72935238246441e-09	6.72737876272755e-06
PLCG1 HCK GAB1 PIK3CG SRC	50.0728209245472	4.54566573271364e-09	7.03411725769265e-06
EGR1 FOS CREBBP JUN RELA SRC	50.0100675076897	4.6792730801215e-09	7.03411725769265e-06
WNT7B WNT9A FZD5 LRP6	48.9691886140849	7.5625704587523e-09	1.04939391158025e-05
GNA15 PLCB3 QRFPR	48.378489464288	9.92724447090865e-09	1.27580680507888e-05
GNA15 PLCB3 HCRTR2	48.234248482658	1.06087377771402e-08	1.27580680507888e-05
ARHGAP36 RHOC ARHGEF16 RAC2	47.6670433568832	1.37715659986881e-08	1.55265799406459e-05
ARHGAP6 RHOC ARHGEF16 RAC2	46.9628629367113	1.90331873595184e-08	2.01964509869619e-05
ARHGAP31 RHOC ARHGEF16 RAC2	46.7819059681436	2.06821235693155e-08	2.07269348370491e-05
ARHGAP11B RHOC ARHGEF16 RAC2	46.4564645967245	2.40137268958662e-08	2.27991378670806e-05
AKT1 EGR1 FOS CREBBP SRC	45.9696859591013	3.00197845470862e-08	2.70763446722444e-05
WNT7B WNT8B FZD5 LRP6	45.7765611803967	3.27979132741518e-08	2.81734075024964e-05
ARHGAP6 RHOC NGEF RAC2	45.1933693502952	4.28386520701096e-08	3.51257474860322e-05
ARHGAP36 RHOC NGEF RAC2	45.0059588803268	4.66747013261326e-08	3.66071711835698e-05
ARHGAP11B RHOC NGEF RAC2	44.7402050610678	5.27076434719831e-08	3.91036419751778e-05
ARHGAP31 RHOC NGEF RAC2	44.6794194659418	5.41931952646735e-08	3.91036419751778e-05
CDC42 RHOC PLEKHG5 RAC2	44.3439989414209	6.31725022293139e-08	4.38295679890228e-05
MYO9B NGEF RHOB RAC2	43.9673705758932	7.50304387509004e-08	5.01286698010183e-05
RARA NR4A1 THRB RXRG NCOR2	43.512229470681	9.23514060779951e-08	5.89779503610583e-05
WNT7B WNT8B FZD10	43.4545190279758	9.48145995049998e-08	5.89779503610583e-05
WNT7B WNT9A FZD10	42.6301386998142	1.38045023412303e-07	8.30064725778179e-05
WNT9A WNT2 WNT2B SFRP1 SFRP2	42.5281744789838	1.44603665930276e-07	8.41453396682663e-05
GNA15 PLCB3 OXTR	42.0033245800622	1.83599371927912e-07	0.000103498408443987
WNT7B WNT8B FZD8 LRP5	41.5107300559899	2.29655657309193e-07	0.000125538634000161
PLCG1 SYK HCK PIK3CG SRC PIK3CD	40.8045590199694	3.16402383626624e-07	0.000167870076418843
RARA THRB NR4A2 RXRG	40.5744413176015	3.5118607411011e-07	0.000181001302596351
CACNG2 GRIA1 PRKACA	40.4037538949973	3.79419806684211e-07	0.000190120941466014
PLCB3 SYNJ1 SYNJ2 PIP5K1C PIK3C2G	40.1838982164188	4.19136938711873e-07	0.000204346249660094
PLCG1 PLCG2 SYK HCK PIK3CG PIK3CD	39.9610584901096	4.63612130463531e-07	0.000220081558458727
WNT7B WNT9B SFRP1 SFRP2	39.7120272500562	5.18886953848607e-07	0.000240005173345514

CDC20 CDK1 CCNF PLK1 CDC6	39.1193713820466	6.78212199622941e-07	0.000305856746724956
RARA CREBBP RXRG NCOR2	38.6363703347645	8.43352891743976e-07	0.000371054702784624
AKT1 RARA ESR1 NR4A1 NCOR2	38.5261816280887	8.8630618599872e-07	0.00038066850688645
RARA EP300 ESR1 NR4A1 NCOR2	38.4698452633776	9.09100132617802e-07	0.000381378076564942
EP300 EGR1 FOS CREBBP JUN RELA	38.3037527279365	9.79749113505513e-07	0.000401674869511953
WNT8B WNT2 WNT2B SFRP1 SFRP2	38.0598816461823	1.09348339427662e-06	0.00043834104331902
ADRA1A GNA15 PLCB3 ADRA1B	37.9617173548793	1.14288510444371e-06	0.000448184878240438
VDR NR4A1 RXRG NCOR2	37.6075036942091	1.34030994947132e-06	0.000514422365500279
GNA15 PLCB3 PIK3C2G	37.1663667782001	1.63415616549756e-06	0.00061413631394605l
PPARG NR4A1 RXRG NCOR2	36.7385411518706	1.98006636531467e-06	0.000728947289059415
MAPK14 FOS SYK MAPK11 JUN SRC LYN	36.4011996931694	2.30332885275963e-06	0.000830994983498621
RARA ESR1 NR4A1 THRB NCOR2	36.2382018780934	2.47779772311052e-06	0.000876411629944915
RARA NR4A2 RXRG NR0B2	35.983654566271	2.77686394345444e-06	0.000963304782230283
ARHGDIB CDC42 RHOC RAC2	35.9335542744925	2.83981790860643e-06	0.000966556136855688
MYO9B RHOG ARHGEF16	35.8872654698183	2.89924136054331e-06	0.000968507683385942
PLCG1 JAK2 GAB1 KIT PIK3CG	35.5903384534648	3.31090214045204e-06	0.00108591570384753
WNT7B WNT8A SFRP1 SFRP2	35.3840120572437	3.63063396002783e-06	0.00116219656503117
RHOC ARHGEF16 RAC2 ARHGAP11A	35.3584437613061	3.67233240239351e-06	0.00116219656503117
LPAR1 GNA15 PLCB3	35.3043614278625	3.7621064743476e-06	0.00117007997742684
THRA NR4A2 NR2F1 RXRG	35.0616972401407	4.19249624383777e-06	0.00128183796173881
EP300 EGR1 FOS NFATC2 CREBBP JUN	34.9834836198275	4.34137117955036e-06	0.00130523324513181
WNT9A WNT2 FZD5 WNT2B LRP6	34.9023606932097	4.50132922247271e-06	0.00133113898105222
WNT10A SFRP1 SFRP2	34.671521424046	4.98921832914334e-06	0.00145162111999059
CGN TJP1 TJP2	34.6217578746682	5.10108483853244e-06	0.00145994483648734
VDR NR4A2 RXRG	34.5874333472483	5.17969230751092e-06	0.00145994483648734
WNT9A WNT16 SFRP1 SFRP2	34.5218380467795	5.33327301566189e-06	0.0014801063373773
SYK SOS1 RAC2 PIK3CD	34.3184104104594	5.83889364069723e-06	0.00159587579370511
CDC20 CDK1 CCNF CDK2 CDC6	34.0765819803637	6.50216082154209e-06	0.00175063401581788
WNT7B SFRP5 WNT8B	33.8230719689251	7.27780623099861e-06	0.00193065215589682
RHOC NGEF RAC2 ARHGAP11A	33.755471209642	7.49970662561328e-06	0.0019606841712962
PLCG1 PLCG2 SYK LYN TEC	33.6948698129674	7.70433036734186e-06	0.001985405935664
PLCG1 PLCG2 SYK PIK3CG LYN PIK3CD	33.6224330839694	7.95619502957923e-06	0.00202143383293774
WNT7B WNT9A FZD2 FZD1	33.5765518366586	8.11993356653051e-06	0.00203438168898117
TRHR GNA15 PLCB3	33.4616887894798	8.5446577060555e-06	0.00211146685424021
SYNJ1 PLCD1 PIP5K1C PIK3C2G	33.3642036265086	8.92236207428176e-06	0.00217500661429687
SYNJ1 PIP5K1C PIK3C2G PLCZ1	33.2793756503639	9.26447229399052e-06	0.0022282908761506
PLCB2 SYNJ1 PIP5K1C PIK3C2G	33.2268896816649	9.48263067768185e-06	0.0022507522999303
PLCB1 SYNJ1 PIP5K1C PIK3C2G	33.175705817089	9.70028239066778e-06	0.00227251161097735
PLCB3 SYNJ1 PIK3CG SYNJ2 PIP5K1C PIK3CD	33.0495911814933	1.02579406958681e-05	0.00237234605400979
MYO9B RHOG NGEF	32.922502810152	1.08520593187222e-05	0.00247797845633456
OCLN TJP1 TJP2	32.8830345296683	1.10434201551213e-05	0.00249015320222791
CACNB3 CACNA1C CACNA2D1	32.6071057927806	1.24780173648142e-05	0.00277890068202326
SMAD3 HDAC1 ATF3 JUN	32.4885861450693	1.31495827895289e-05	0.00289274785293064
GNA15 PLCB3 AVPR1A	32.3888890478782	1.37421563475293e-05	0.00297181999437293
PLCB2 ADRA1A GNA15 ADRA1B	32.3730820911471	1.38385098690241e-05	0.00297181999437293
ADRA1A GNA15 PLCB1 ADRA1B	32.3088464542448	1.42370068278996e-05	0.00302142783727625

GNA15 PLCB3 GRPR	32.2255117932349	1.47709839364296e-05	0.0030982997584797
EGR1 MAPK1 FOS MAPK8 JUN SRC	31.9416566788886	1.67432119215771e-05	0.00347161838911873
APC2 JUP CDH1	31.8316872832719	1.75755673432754e-05	0.00360279158301528
CD36 SRC LYN	31.7384324454894	1.8313453144847e-05	0.00371186945258309
CARD11 PDPK1 PRKCQ MAP3K7	31.6898180104563	1.8710209617967e-05	0.00374637536007764
WNT10A FZD5 LRP6	31.66703591263	1.88990607997708e-05	0.00374637536007764
ADRA1A GNA15 PLCB3 ADRA1D	31.6348804376654	1.91688309921778e-05	0.00375854937247713
UBC FOS RB1 JUN RUNX2	31.5666818957685	1.97536805744969e-05	0.00383157681594999
EP300 FOS RB1 JUN RUNX2	31.4987257705381	2.03540342460506e-05	0.00389002299315658
AR FOS RB1 JUN RUNX2	31.4840224499839	2.0486289946775e-05	0.00389002299315658
WNT8B WNT2 FZD5 WNT2B LRP6	31.4341482440488	2.09412812453014e-05	0.00392579539284893
AKT1 PIK3CG RAC2 PIK3CD	31.4159332451564	2.11099369757939e-05	0.00392579539284893
CDC42 PIK3CG RAC2 PIK3CD	31.3109958652498	2.21081104274656e-05	0.0040694714693985
UBC FOS ESR1 JUN RELA SRC	31.1944330846868	2.32717565278318e-05	0.00424039612126825
PTPN6 GAB1 EGFR SRC	31.1309213870114	2.39311241133633e-05	0.0043169354788096
RARA ESR1 THRB NR4A2	30.9882579265557	2.54804726270708e-05	0.00450908968244151
UBC FOS SYK JUN SRC LYN STAT3	30.9803135037291	2.55696127859917e-05	0.00450908968244151
SRF EGR1 FOS CREBBP	30.96465361418	2.57462296852085e-05	0.00450908968244151
KCNH4 KCNQ2 KCNQ1	30.8887290727117	2.66198102600868e-05	0.00461725728155485
GNA15 PLCB3 GHSR	30.8008516636922	2.76676284870492e-05	0.00475329857407505
WNT8B WNT16 SFRP1 SFRP2	30.6470371853405	2.96008938877046e-05	0.00499484520755704
UBC VCL ACTN1 SRC CDH1	30.6449991769593	2.96273871727148e-05	0.00499484520755704
PTPN11 HCK GAB1 PIK3CG SRC	30.6164587968858	3.00008771065841e-05	0.00501097983449694
ARHGDIA CDC42 RHOC RAC2	30.594128560534	3.02963468697959e-05	0.00501390643288301
WNT9A WNT5B SFRP1 SFRP2	30.5082992469776	3.14590950707405e-05	0.00515900559982807
AKT1 RARA ESR1 CREBBP NCOR2	30.3820478557908	3.32502017046377e-05	0.00540360710405368
EP300 PPARG ESR1 NR4A1 NCOR2	30.2376515774028	3.54227707692889e-05	0.00570528001702859
CACNG2 GRIA1 DLG1	30.0528172043179	3.84099446424369e-05	0.00613165479119397
NR4A1 THRA RXRG NCOR2	29.9919081658471	3.94479288149396e-05	0.00624211568326926
PLCD4 SYNJ1 SYNJ2 PIP5K1C PIK3C2G	29.8713832549122	4.15843775797287e-05	0.00652296162748457
GNA15 PLCB3 PROKR2	29.7432709341114	4.39809642140965e-05	0.00683941908153523
FYB SKAP1 LCK	29.3625585293389	5.19394030484843e-05	0.00800799052642401
WNT2 WNT9B WNT2B SFRP1 SFRP2	29.3420171659538	5.2407166497348e-05	0.00801163454614967
WNT7B FZD10 WNT8A	29.2770944891824	5.39131484529332e-05	0.00817259903312993
ARHGEF11 MYO9B RHOC RAC2	29.2130756837912	5.54400726100468e-05	0.00833402891510528
AKAP13 MYO9B RHOC RAC2	29.1450784988904	5.71087537135639e-05	0.00851205231589593
WNT9A WNT5A SFRP1 SFRP2	29.1194012638207	5.7751726671551e-05	0.00851205231589593
WNT7B WNT8B FZD2 FZD1	29.1079831754161	5.80399376270968e-05	0.00851205231589593
PSEN1 CTNND2 CDH1	29.0112510115076	6.05393970932733e-05	0.00880701761423836
ATF2 FOS DUSP10 MAPK13	28.9787295381615	6.14034046386713e-05	0.00886124813021594
PLCG1 SYK HCK PIK3CG SRC CBL	28.9039214363404	6.34374239359969e-05	0.00908212452683689
WNT7B WNT9A FZD8 LRP6	28.8016052239887	6.63276780630273e-05	0.00942114161085787
RARA ESR1 NRIP1 NCOR2	28.6965779415893	6.94299579645508e-05	0.00978474227908227
WNT7B WNT9A SFRP1 FZD6	28.6760626262778	7.00524789388757e-05	0.00979594315952231
SRGAP3 CDC42 RHOC RAC2	28.6404711577778	7.11455952410178e-05	0.00981616644043802
JAK1 PLCG1 INPP5D GAB1 PIK3CG	28.6359573747511	7.1285426226364e-05	0.00981616644043802

PLCG2 RAC2 ERBB2	28.4969001187867	7.57289573065645e-05	0.0103490504610085
ITGA2 ITGB1 COL1A1	28.3358726804886	8.12175769320245e-05	0.0110156681975698
RARA NR4A1 RXRG NR0B2	28.272947363157	8.34676785458344e-05	0.0112112362753675
FN1 ITGA5 ITGB1 COL1A1	28.2609813679027	8.3902483351328e-05	0.0112112362753675
WNT7B FZD5 WNT8A LRP6	28.2059889685213	8.59296908479745e-05	0.0113976889206369
GRAP2 KIT LYN TEC	28.1669648675896	8.73975481037181e-05	0.0115077691258611
VDR CREBBP RXRG NCOR2	28.0520143594651	9.18668746556772e-05	0.0120085982022736
PLCG1 PLCG2 DAPP1 LCK	27.9715650736977	9.51284115033868e-05	0.0123454778065438
GNA15 PLCB3 CCKBR CCKAR	27.9427772607904	9.63231016192223e-05	0.0124112316436368
MYO9B CDC42 ARHGEF16 RHOA RAC2	27.9090397727876	9.77420843949472e-05	0.0125047479461025
PLCG1 INPP5D PIK3C2G	27.8838748780116	9.8813940723641e-05	0.0125528498360124
WNT9A WNT2 SFRP1 WNT3A SFRP2	27.7914902131575	0.000102849446886544	0.0128917167361238
WNT9A WNT2 FZD10 WNT2B	27.778951425619	0.000103409565157975	0.0128917167361238
ITGB7 ITGAE CDH1	27.77413724378	0.000103625418633957	0.0128917167361238
CDC6 MYC KAT2A	27.7115968285336	0.000106470333020581	0.0131549201188922
EGR1 SUMO1 FOS CREBBP JUN	27.6240961397605	0.000110580605941268	0.0135698200719356
PPARG CREBBP RXRG NCOR2	27.5509430976001	0.00011413693052631	0.0139115952011088
EGR1 ATF3 MAPK8 JUN	27.5054371080834	0.000116405959871058	0.0140929336249262
RARA ESR1 NR4A2 NR0B2	27.4411569947162	0.000119687222619458	0.014393585392216
LPAR1 PLCB3 GNAI1 GNAO1	27.4166263737513	0.000120963364271809	0.0144507160801269
MYO9B CDC42 RHOC ARHGEF3 RAC2	27.3900743285181	0.000122359815246997	0.0145213730739512
COL7A1 COL4A1 THBS1	27.3388553271592	0.000125098711169724	0.0147152888466737
PLCG1 PLCG2 SYK BLNK VAV1	27.3291351802324	0.000125625283130315	0.0147152888466737
NR4A1 THRA NR2F1 RXRG	27.3028784522932	0.000127058664552426	0.0147871693539433
AR SMAD3 NR3C1 FOS CREBBP JUN	27.2689282769885	0.000128936008387925	0.0149094657391653
CDC42 RHOC RAC2 TRIO	27.1961811994592	0.000133051439908605	0.0152873562070785
MYO9B RHOBTB1 ARHGEF16	27.1459848996085	0.000135966503748697	0.0155221790995511
WNT7B WNT8B FZD8 LRP6	27.1315542931186	0.000136816147060737	0.0155221790995511
EGR1 MAPK1 FOS MAPK3 JUN	27.0310626219519	0.000142880027751424	0.0161088301287996
KCNH4 KCNV2 KCNC1	26.9956362967551	0.0001450803827312	0.0162553107086218
NCOA6 RARA EP300 ESR1 CREBBP NCOR2	26.9213492425526	0.000149803621229627	0.0166809106380324
CCNF CDK2 FBXW7 CDC6	26.8485672909132	0.000154578309638165	0.0171006247892552
WNT9A WNT4 SFRP1 SFRP2	26.835238574078	0.000155468843363704	0.0171006247892552
EGR2 EP300 CREBBP JUN	26.7755598471288	0.000159518676488779	0.0174397418495823
EP300 FOS JUN RUNX2 STAT3	26.7150994681548	0.000163727725907115	0.0177920749857737
AR FOS JUN RUNX2 STAT3	26.6303641630664	0.000169811770570738	0.0183427217324883
UBC FOS JUN RUNX2 STAT3	26.4445199025761	0.000183947749246682	0.0197513895753625
PLCG1 DAPP1 SRC LYN	26.3699213638104	0.000189943153357852	0.0201806666064622
MAPK14 FOS SYK JUN RELA SRC	26.3669841169678	0.000190183121187348	0.0201806666064622
WNT9A FZD5 WNT16 LRP6	26.3429132291878	0.000192160973871203	0.0202712971208341
UBC FOS SYK JUN RELA SRC STAT3	26.301736681909	0.000195591522860528	0.0205132295400061
MAPK7 FOS ETS1 JUN	26.2617324198458	0.000198982253610147	0.0207482131379968
WNT2 WNT8A WNT2B SFRP1 SFRP2	26.2474281351811	0.000200208696432558	0.020756118821534
CDK1 FZR1 CCNB1 CCNA1 CDC25A PLK1 CDC6 CCNB2	26.2094319940982	0.000203502801849331	0.0209770688146291
FN1 SPARC COL1A1	26.1628111735907	0.000207617757702616	0.0212796405181676
WNT9A SFRP1 WNT7A SFRP2	26.1239122999116	0.000211113873979762	0.0215157241396663

PPARG NR4A2 RXRG NR0B2	26.0821768787099	0.000214929524524177	0.0217815376005147
PLCG1 PLCG2 SYK TEC VAV1	26.004611917084	0.000222202442909603	0.0223927925566834
FOS SP1 JUN MAPK13	25.928212942528	0.000229603184066507	0.0229027534745208
UBC IQGAP1 EGFR CTNNB1 SRC CDH1	25.9190758915583	0.00023050440356942	0.0229027534745208
IQGAP1 CDC42 EGFR CTNNB1 SRC CDH1	25.913343092634	0.000231071629933077	0.0229027534745208
KCNH4 KCNQ2 KCNQ3	25.8721400373913	0.000235189129086932	0.0231834792327824
WNT8B WNT5A SFRP1 SFRP2	25.8107401883403	0.000241459529904176	0.023672219890986
PLCB3 GNA11 QRFPR	25.7667095654351	0.000246057332121463	0.0239925849412923
MAPK14 FOS SYK NFKB1 SRC LYN	25.7480340462725	0.000248033458339014	0.0240552449192338
WNT7B WNT9A WLS	25.713963654031	0.000251678971885938	0.0242650410606448
RARA NR4A2 RXRG RARB	25.7027813265905	0.000252886951571663	0.0242650410606448
CACNA1C RYR2 PRKACA	25.6851479699831	0.000254803433399564	0.0243195721433584
VDR SMAD3 RUNX2	25.5745673198054	0.000267152592773434	0.0253640295844209
VAV2 SYK RHOG SOS1	25.5570806429516	0.000269158725755725	0.0254207029000394
PLCB2 SYNJ1 PIK3CG PIP5K1C PIK3CD	25.5321474360699	0.000272044858848464	0.0255594646289971
SYNJ1 PLCD1 PIK3CG PIP5K1C PIK3CD	25.4983461585815	0.000276006278447483	0.0257972914866018
SYNJ1 PIK3CG PIP5K1C PLCZ1 PIK3CD	25.4366116794924	0.00028338870224176	0.0263507670089645
PLCB1 SYNJ1 PIK3CG PIP5K1C PIK3CD	25.4235578211305	0.000284974480012967	0.0263623315125842
UBC EZR ACTN1 CDH1	25.3770945066954	0.000290690119877035	0.0267538728186828
PLCG1 PLCG2 DAPP1 LYN	25.3460226927976	0.000294575281335918	0.0269267862581376
FN1 THBS1 HRG PLG	25.3382567962664	0.000295554281230181	0.0269267862581376
SOS1 RHOC RAC2 ERBB2	25.2941531545496	0.000301175283263011	0.0273010097225199
PLCG1 PLCG2 PIK3C2G	25.2300995461407	0.000309526991264186	0.0279177869770733
FOS SYK NFKB1 SRC LYN STAT3	25.2018453466345	0.000313283099805939	0.0281159892407927
UBC SMAD2 SMAD3 FOS JUN RUNX2	25.1859102513802	0.000315421280794648	0.0281677449715577
EP300 SMAD2 SMAD3 FOS JUN RUNX2	25.1400598344666	0.000321654055890574	0.0285828448975866
WNT7B FZD8 LRP5 WNT8A	25.0830991270988	0.000329566368529655	0.029142390793659
WNT8B WNT5B SFRP1 SFRP2	25.0046765248861	0.00034077449212655	0.0299864929925407
PTEN AKT1 EGR1 FOS SRC	24.9522295970682	0.000348478870449087	0.0305155841943256
GNA15 PLCB3 LPAR3	24.9140034168003	0.000354202100068091	0.0308669163436149
MTNR1A RGS20 MTNR1B GNAI1 GNAI3	24.8822594583836	0.000359025099553989	0.0310250157379448
MYO9B CDC42 RHOC NET1 RAC2	24.8794432891595	0.000359456083443122	0.0310250157379448
UBC CDK1 JUN SRC LYN	24.8514131831575	0.000363773659174726	0.031248157323109
DOK1 GRB2 KIT LYN TEC	24.7788837113858	0.000375184133624251	0.0320755762390894
WNT9A WNT1 SFRP1 WNT3A SFRP2	24.7371364563319	0.000381910976319899	0.0324966608577107
MAPK14 FOS ESR1 MAPK11 JUN SRC	24.7217792483034	0.000384415289667261	0.0325561850249189
SYK PIK3CG RHOA RAC2	24.6900590709469	0.000389639234875783	0.0328444026071226
WNT7B WNT6 SFRP1 SFRP2	24.6517666841553	0.000396038746026783	0.0332285718119867
UBC MAPK1 FOS ESR1 JUN EGFR SRC	24.5915974228974	0.000406304032684157	0.0338931655776084
MYO9B CDC42 NGEF RHOA RAC2	24.5786669292467	0.000408544028038027	0.0338931655776084
APC2 CDC27 CDH1	24.5726159355246	0.000409596435274606	0.0338931655776084
UBC SMAD3 NR3C1 FOS JUN	24.5525927516604	0.000413097994377387	0.034026825208099
CDK4 CDK1 CCNF CDK2 CDC6	24.5087966796729	0.00042085989647056	0.0345085985110565
PLCD4 SYNJ1 PIK3CG SYNJ2 PIP5K1C PIK3CD	24.4563172830103	0.000430350006616731	0.0351270758794534
UBC FYN PIP5K1C CDH1	24.4336517764708	0.000434513574300355	0.035171274895751
AKT1 RARA ESR1 CREBBP SRC	24.4228141475949	0.000436518394243079	0.035171274895751

WNT8B WNT2 FZD10 WNT2B	24.4166605361965	0.000437660782131366	0.035171274895751
WNT8B FZD5 WNT16 LRP6	24.4111276903893	0.000438690440243028	0.035171274895751
MAPK1 FOS AGT SRC	24.4004600176703	0.000440682420969951	0.0351746468667122
PSEN1 JUP CDH1	24.3711167614825	0.000446207747223704	0.03545877335757
PLCB2 ADRA1A GNA15 ADRA1D	24.3257251984927	0.000454889565787986	0.0359901441984626
ADRA1A GNA15 ADRA1D PLCB1	24.2281851134257	0.000474111947185274	0.0373471852195422
MEF2D MEF2C MAPK13	24.2143415122133	0.000476904123065891	0.0374037977216766
PLCG1 INPP5D PIK3CG LYN PIK3CD	24.1973208541334	0.000480359280207066	0.0375063901213522
WNT7B WNT8B SFRP1 FZD6	24.1874683661122	0.00048237055868694	0.0375063901213522
WNT8B WNT2 SFRP1 WNT3A SFRP2	24.1446413321166	0.000491209964211037	0.0380297705768365
WNT9A WNT2 FZD2 FZD1 WNT2B	24.111838933834	0.000498087921181556	0.0383974701290346
PLCG2 SYK RAC2 PIK3CB	24.0290637856459	0.000515868155232524	0.0395989176691043
ESR1 THRA NR4A2 NR2F1	24.0032285839404	0.00052154439437313	0.039771942552891
PLXNA4 RRAS RND1	23.9987628937434	0.000522531758137101	0.039771942552891
MYO9B ECT2 CDC42 RHOC RAC2	23.955082519155	0.000532286965767459	0.0403442209053748
CDC42 EGFR CTNNB1 SRC MET CDH1	23.9198051059592	0.000540296112324201	0.0407799228879342
UBC EGFR CTNNB1 SRC MET CDH1	23.9081915699752	0.000542958617937628	0.0408101271207369
MYO9B ARHGEF3 RHOB RAC2	23.8776703043115	0.000550017570168371	0.0411691574616898
CDC42 RHOC ARHGAP15 RAC2	23.867750979606	0.000552331086899782	0.041171489572666
LCP2 PLCG1 PLCG2 SYK LYN	23.827695469066	0.000561771125249133	0.0417028367422597
SOCS3 MAPK11 SRC	23.8084877808187	0.000566353992628499	0.0418707363648586
NMUR2 GNA15 PLCB3	23.711408409532	0.000590085190311451	0.0434471295837889
WNT8B WNT4 SFRP1 SFRP2	23.63497545745	0.00060945493298592	0.0446908842932236
ESR2 FOS ESR1 MAPK11 SRC	23.5924406984273	0.000620503509955461	0.0453168535064233
PRKD1 PKD1 CDH1	23.5807092499191	0.00062358529369777	0.0453367057387823
PLCG1 SYK PIK3CG SRC LYN CBL	23.5723066259536	0.000625801858692654	0.0453367057387823
MED1 RARA ESR1 NR4A1 THRB	23.5386900468358	0.000634747471650265	0.0458008385643966
PLCG1 JAK2 GAB1 PIK3CG SRC	23.5199902318251	0.000639777918882145	0.0459798959311355
CDK1 FZR1 CCNB1 CCNA1 CDC25A CDK2 CDC6 CCNB2	23.5080997030554	0.000642996958465925	0.0460278656101858
MYO9B RHOBTB1 NGEF	23.4951082998976	0.000646532232910335	0.0460460230405902
PRKACG ADCY9 GNAO1 PRKACA	23.4884344289825	0.000648355776501464	0.0460460230405902
PLCB3 GNA14 QRFPR	23.4279803202563	0.000665106038667873	0.0470503836530579
NCOA6 RARA ESR1 CREBBP SRC	23.4106027445947	0.000669999127376419	0.0472113838232157
WNT9A FZD10 WNT16	23.2917394153463	0.000704429824918473	0.0494443953762815

Supplementary Table S21. Statistical data for GAGE analysis of suggested gene set, identified in PPI network cliques of orthologues 1:1:1 mapped between human, rat and zebrafish expression enriched in whole brain of stressed zebrafish vs. control groups (Experiment 4). Analysis was performed on normalized and log2-transformed counts by general applicable gene set enrichment for pathway analysis (GAGE) package [S4], using two-tailed t-test for group comparison of bidirected differential expression of gene set. False discovery rate cutoff (q.val) was set at 0.05. p.geomean – geometric mean of the individual p-values from multiple single array based gene set tests, stat.mean - mean of the individual statistics from multiple single array based gene set tests.

	**p.geomean**	**stat.mean**	**p.val**	**q.val**	**set.size**
**Zebrafish Whole Brain**					
**Stress vs. Control**	0.0158350100655709	2.11001541435221	1.30103998855426e-07	1.30103998855426e-07	225
**Fluoxetine vs. Control**	0.0289336645708696	1.85760441987371	2.8526272304413e-06	2.8526272304413e-06	231
**Fluoxetine vs. Stress**	0.198908481814815	0.803149224907988	0.0247120154155718	0.0247120154155718	225
**Human MDD vs. Control**					
**Hippocampus**	1.75184095561863e-10	6.41840434724109	1.16588704029555e-27	1.16588704029555e-27	224
**PFC**	0.000506647782182357	3.23210250919084	6.86491091131674e-11	6.86491091131674e-11	245
**Rat Hippocampus**					
**Stress vs. Control**	0.000100320850914349	3.71032421694908	9.77483087800069e-11	9.77483087800069e-11	220
**Fluoxetine vs. Control**	0.00248702361905806	2.70663534545235	1.57116830988579e-06	1.57116830988579e-06	242
**Fluoxetine vs. Stress**	0.000376851964266212	3.28687154021419	8.38127501663008e-09	8.38127501663008e-09	220

Cited references:

S1 Szklarczyk, D. *et al.* STRING v11: protein–protein association networks with increased coverage, supporting functional discovery in genome-wide experimental datasets. *Nucleic acids research* **47**, D607-D613 (2019).

S2 McKenzie, A. T. *et al.* Brain cell type specific gene expression and co-expression network architectures. *Scientific reports* **8**, 1-19 (2018).

S3 Love, M. I., Anders, S. & Huber, W. (2019).

S4 Gearing, L. J. *et al.* CiiiDER: A tool for predicting and analysing transcription factor binding sites. *PloS one* **14**, e0215495 (2019).

S5 Marot, G., Jaffrézic, F. & Rau, A. metaRNASeq: Differential meta-analysis of RNA-seq data. *dim (param)* **1**, 3 (2020).
